# Efficacy of a compulsory homework programme for increasing physical activity and improving nutrition in children: a cluster randomised controlled trial

**DOI:** 10.1186/s12966-019-0840-3

**Published:** 2019-09-05

**Authors:** Scott Duncan, Tom Stewart, Julia McPhee, Robert Borotkanics, Kate Prendergast, Caryn Zinn, Kim Meredith-Jones, Rachael Taylor, Claire McLachlan, Grant Schofield

**Affiliations:** 1 School of Sport and Recreation, Auckland University of Technology, Private Bag 92006, Auckland, New Zealand; 2 Edgar Diabetes and Obesity Research Centre, University of Otago, PO Box 56, Dunedin, New Zealand; 3 School of Education, Federation University Australia, PO Box 663, Ballarat, VIC 3353 Victoria Australia

**Keywords:** Child health, Intervention, Education, Curriculum, Pedometers, Dietary assessment, Body size, Child obesity

## Abstract

**Background:**

Most physical activity interventions in children focus on the school setting; however, children typically engage in more sedentary activities and spend more time eating when at home. The primary aim of this cluster randomised controlled trial was to investigate the effects of a compulsory, health-related homework programme on physical activity, dietary patterns, and body size in primary school-aged children.

**Methods:**

A total of 675 children aged 7–10 years from 16 New Zealand primary schools participated in the Healthy Homework study. Schools were randomised into intervention and control groups (1:1 allocation). Intervention schools implemented an 8-week applied homework and in-class teaching module designed to increase physical activity and improve dietary patterns. Physical activity was the primary outcome measure, and was assessed using two sealed pedometers that monitored school- and home-based activity separately. Secondary outcome measures included screen-based sedentary time and selected dietary patterns assessed via parental proxy questionnaire. In addition, height, weight, and waist circumference were measured to obtain body mass index (BMI) and waist-to-height ratio (WHtR). All measurements were taken at baseline (T_0_), immediately post-intervention (T_1_), and 6-months post-intervention (T_2_). Changes in outcome measures over time were estimated using generalised linear mixed models (GLMMs) that adjusted for fixed (group, age, sex, group x time) and random (subjects nested within schools) effects. Intervention effects were also quantified using GLMMs adjusted for baseline values.

**Results:**

Significant intervention effects were observed for weekday physical activity at home (T_1_ [*P* < 0.001] and T_2_ [*P* = 0.019]), weekend physical activity (T_1_ [P < 0.001] and T_2_ [P < 0.001]), BMI (T_2_ only [*P* = 0.020]) and fruit consumption (T_1_ only [*P* = 0.036]). Additional analyses revealed that the greatest improvements in physical activity occurred in children from the most socioeconomically deprived schools. No consistent effects on sedentary time, WHtR, or other dietary patterns were observed.

**Conclusions:**

A compulsory health-related homework programme resulted in substantial and consistent increases in children’s physical activity – particularly outside of school and on weekends – with limited effects on body size and fruit consumption. Overall, our findings support the integration of compulsory home-focused strategies for improving health behaviours into primary education curricula.

**Trial registration:**

Australian New Zealand Clinical Trials Registry, ACTRN12618000590268. Registered 17 April 2018.

**Electronic supplementary material:**

The online version of this article (10.1186/s12966-019-0840-3) contains supplementary material, which is available to authorized users.

## Background

Physical activity and healthy nutrition practices are essential for many aspects of child health and development [1], including the prevention of chronic health conditions in adolescence and adulthood [2, 3]. However, evidence indicates that many children do not meet international physical activity and dietary guidelines [4–7], contributing to a rise in obesity and related comorbidities in later life [8]. The development, implementation, and evaluation of effective and sustainable initiatives that equip children to lead healthy and active lives has become a key public health priority in many countries [1]. Despite parents having a significant influence on children’s activity and eating patterns, exclusively home-based interventions are logistically impractical. The school setting may provide a unique opportunity for intervention delivery: intervention material can be wide-reaching, especially if made compulsory as part of the curriculum or integrated into school policy [9–11].

Several systematic reviews indicate that delivering interventions within school can improve several physical activity and diet behaviours in children [12–14]; however, these effects are often modest at best. Quality of evidence from past randomised controlled trials (RCTs) was criticised in a 2013 Cochrane Review and judged at moderate risk of bias—a reliance on self-reported outcomes, absence of longer term follow-up, lack of blinding, and failing to account for clustering during analyses being common problems [12]. Additional reviews have concluded that physical activity interventions have had only small to negligible measured effects on total and moderate-to-vigorous physical activity, whether assessed immediately post intervention or at six-month follow-up [15, 16]. Nonetheless, evidence indicates that school-based physical activity and dietary interventions can be successful in improving indicators related to obesity in children [17].

To date, the majority of school-based physical activity/nutrition interventions have focused solely on the school environment [12, 18], despite reviews indicating that multicomponent interventions involving the family or community in addition to the school are likely to be most effective [17, 19, 20]. Family support and parental restrictions can influence out-of-school physical activity [21], and the majority of food that children consume originates from the home [22, 23]. Therefore, it is unlikely that long-term behaviour change is possible when the influences of the home environment are not addressed. The concept of curriculum-based physical activity or nutrition ‘homework’ is relatively under-utilised. Active for Life Year 5 (AFLY5) aimed to improve in-school and out-of-school physical activity and diet behaviours through professional learning and development for teachers coupled with in-class lessons and homework plans [24]. While positive effects on screen time and high energy drinks and snacks were observed, there were no significant effects on physical activity, fruit and vegetable consumption, or body size [25, 26].

This paper presents the main findings from the Healthy Homework programme, an eight-week (approximately one school term) intervention designed to improve the physical activity and dietary behaviours of primary-aged children through a compulsory health-related homework schedule, supplemented with curriculum resources for teachers. This approach differs from many previous school-based interventions because the homework component was designed to maximise family engagement, theoretically improving the likelihood of success. Healthy Homework was developed in 2008 with oversight from New Zealand education and health professionals, and underwent extensive piloting in two primary schools [27]. Despite a small sample size, significant benefits were observed in physical activity and some dietary behaviours. Perhaps most importantly, focus groups revealed that the programme was highly valued by the children, their parents, and participating teachers [27]. We found that the level of family engagement exceeded expectations, and that the usability and utility of Healthy Homework for teachers was high [27].

Despite these encouraging results, several limitations were noted: the sample size was small limiting generalisability, the programme required updating to align with the New Zealand Curriculum, body size was not assessed, and the cluster unit was classroom (raising the possibility of between-group contamination). Therefore, the aim of this study was to determine the effects of an updated Healthy Homework programme on physical activity, nutrition, and body size in a large sample of New Zealand children randomised at the school level.

## Methods

### Participants

Due to the nature of the intervention, individual participants were accessed via primary (elementary) schools, which acted as the cluster unit in all subsequent analyses. Eligibility criteria for the schools were as follows: enrolment of over 100 students, location within Auckland or Dunedin cities, and a contributing, full primary, or composite structure that included at least one class each of students in school years 3–5. A total of 16 primary schools from Auckland (*n* = 10) and Dunedin (*n* = 6) were randomly selected to participate in the study from a sampling frame of all eligible schools. Socioeconomic decile ratings of participating schools (determined by the NZ Ministry of Education) ranged from 1 to 10 (where 1 indicates ‘low’ and 10 indicates ‘high’; median [IQR] = 8 [6–9]). After stratification by decile group (1–7 and 8–10), each school was randomly assigned into the intervention group (*n* = 8) or the waitlisted control group (n = 8) by the lead researcher using a random number generator (concealed from participants; 1:1 allocation ratio). This stratification was implemented as only one Decile 1 school was recruited. Schools assigned to the control group were offered the intervention (including all resources) following the final assessment period. One Year 3, Year 4, and Year 5 class from each school were then selected to participate; simple random sampling was used in instances where there were two or more classes per year. Year 6 classes were excluded to permit final follow-up measurements. Enrolment occurred between 01/07/11 and 24/07/12, with measurements staggered across every calendar month (excluding January and December) and school term (1–4); the last follow-up measure occurred at 07/04/13. At the intervention schools, all children in the selected classes received the Healthy Homework programme as part of the schools’ curricula; however, parental consent was required before children were included in the intervention evaluation. All children in each participating class were invited to take part in the evaluation (i.e., no formal inclusion or exclusion criteria aside from consent/assent), which took place in school grounds. Parent information and consent forms were sent home with the children for completion and return. Ethical approval was obtained from the Auckland University of Technology (10/159) and University of Otago (11/084) Ethics Committees.

Based on reference data from the HH feasibility phase [27], we calculated that a target sample size of 800 children would enable us to detect the following differences in our outcome measures (α = 0.05, 1-β = 0.80): 1000 pedometer steps/day, 30 min.day^− 1^ of TV/computer time, 0.5 daily servings of fruit and vegetables, 0.5 daily servings of takeaway food, and a BMI z-score of 0.2. These calculations allow for school clustering effects (cluster size = 16; ICC = 0.04), correlation between repeated measures (0.4 for all behavioural variables and 0.8 for BMI z-score), and 25% dropout at each follow-up point.

### Intervention design

The design and theoretical basis of the Healthy Homework programme is described in detail elsewhere [27] and only a brief summary is outlined here. Healthy Homework was an eight-week curriculum-based homework schedule, complemented by an in-class teaching resource, designed to promote physical activity and healthy eating in children (curriculum resources may be provided upon request). The programme was developed under the guidance of an advisory committee comprising experienced health and education professionals (including classroom teachers) with regular input from children and parents. Healthy Homework was based on several established behaviour change theories (including the theory of reasoned action, the theory of planned behaviour, and social-cognitive theory [28]), and was designed to support the achievement objectives associated with the Health and Physical Education strand of the New Zealand Curriculum [29]. The research team provided professional learning for the teachers of the three intervention classes at each intervention school, and to one lead teacher in each of the control schools (who were permitted to implement the programme at the conclusion of the final follow-up point). The professional learning protocol was standardised across all schools, and necessitated one half-day release per teacher. Approximately 90 min was spent providing information about the benefits of physical activity and a healthy diet for children’s overall health and development, and the results of previous strategies to integrate these topics into the primary school curriculum. An additional 90 min was spent introducing the teachers to the in-class and homework modules, discussing examples of how to complete tasks, and fielding questions about the programme delivery and evaluation.

At the start of the intervention, all children in participating classes received a homework booklet organised into weekly topics that each contained one physical activity and one nutrition component (e.g., walking and fruit/vegetables, screen time and breakfast, fitness and cooking). Three practical homework options were provided for each topic, and the children were directed by their teacher to complete at least one physical activity and one nutrition task per topic each week (e.g., organising family walks, walking to and from school, limiting screen time, testing the fitness of the family, eating 5+ fruit and vegetables each day, comparing food labels at the supermarket, helping with dinner, preparing a healthy lunch box). Blue or purple rubber wristbands were provided each week for children who completed their homework obligations, with a black colour reserved for those who completed all six tasks on a given week. The intention of the wristbands were to encourage the children to complete more than the minimum number of required tasks. The Healthy Homework classroom curriculum resource was designed to provide the teachers with sufficient educational content and in-class exercises for three 1.5-h sessions delivered on different days throughout each week (including one session reviewing the previous week’s homework). Furthermore, an online portal was developed and monitored to allow teachers to access resources and record student compliance, and to enable children to share homework-related updates with other participants (including those in other schools). Student and teacher modules can be downloaded as Additional files 2 and 3.

### Measures

Baseline measurements were taken prior to intervention delivery (T_0_), and repeated immediately post-intervention (T_1_) and six-months post-intervention (T_2_), between August 2011 and April 2013. The team of research assistants responsible for data collection were not blinded to group allocation. Each research assistant was provided with an appropriate level of anthropometric training by experienced researchers prior to data collection.

#### Physical activity

Weekday physical activity at school, weekday physical activity at home, and weekend physical activity – treated as the primary outcome measures – were assessed using sealed NL-1000 pedometers (New Lifestyles Inc., Lee’s Summit, MO) over five consecutive days (three weekdays, two weekend days). These pedometers have a multiday memory function that automatically stores step counts by day of week for up to 7 days. Our previous research has established the validity of these pedometers for measuring steps in children, with mean percent bias less than 5% for typical walking speeds [30]. Two pedometers were assigned to each child: one clearly labelled ‘School’ and the other ‘Home’. The ‘School’ pedometer was worn during school hours, while the ‘Home’ pedometer was left inside a collection tray in the classroom. At the conclusion of the school day (approx. 3 pm), each child placed their ‘School’ pedometer in the tray and attached their ‘Home’ pedometer. At the beginning of the next weekday (approximately 9 am), the teacher reminded the children to switch over their pedometers again. Thus, before school and after school activities that were outside of the classroom were captured on the ‘Home’ pedometer. This approach was taken as it removed the need for teachers to coordinate a lengthy collection and handout process, and allowed the differentiation of in-school and out-of-school physical activity, while using a cost-effective but objective measurement device.

#### Dietary patterns

Key dietary patterns were estimated as secondary outcome measures using items extracted from the Children’s Dietary Questionnaire (CDQ), a parental proxy report that has been validated in children aged 4–16 years [31]. The CDQ focuses on patterns of food intake over the previous 24 h and/or 7 days rather than actual amounts and types of foods consumed. The questionnaire contained 33 items, 30 of which utilised categorical response options, and three of which utilised open-ended response options. A total of 20 questions referred to dietary patterns over the previous seven-days, 11 questions referred to dietary patterns over the previous 24-h, and two referred to specific meal questions (breakfast and lunch). The four dietary items selected for analyses in this study were: (1) daily fruit consumption, (2) daily vegetable consumption, (3) weekly fast food consumption, and (4) weekly soft drink consumption. These items were selected given their alignment with the New Zealand Food and Nutrition Guidelines, and the high prevalence of soft drink consumption in New Zealand youth [32]. A copy of the questionnaire may be provided upon request.

#### Television and computer usage

Two questions pertaining to the frequency and duration of television, computer and gaming console use were also amended to the questionnaire as additional secondary outcome measures. These questions asked parents to recall the amount of time each day (Monday to Sunday) their child spent (1) watching TV, DVDs, or videos, or (2) on the computer or games console (not including school-related work) in the previous week. The total time provided for Monday to Friday was averaged to provide a weekday TV/computer estimate, whereas Saturday and Sunday totals were averaged to provide a weekend estimate.

#### Body size

Standing height was measured to the nearest millimetre with a portable stadiometer (SECA 213, Hamberg, Germany) and weight was measured to the nearest 0.1 kg on a digital scale (SECA 813, Hamberg, Germany). Waist circumference was measured midway between the iliac crest (highest point of the pelvis at the side) and the lowest rib margin. Participants were asked to remove their shoes and any bulky external clothing (i.e. jackets, coats) prior to assessment. For all anthropometric measures, estimates were taken three times, with the average value used in subsequent analysis. Body mass index (BMI; secondary outcome measure) was calculated as weight (kg) divided by squared height (m^2^), and waist-to-height ratio (WHtR; secondary outcome measure) was calculated as waist circumference (cm) divided by height (cm).

### Statistical analysis

In the first instance, baseline characteristics of the sample were calculated and presented as mean ± standard deviation (SD), or median and interquartile range (IQR) in cases where the data were not normally distributed. Intention-to-treat analyses were used to test the efficacy of the intervention, regardless of adherence to homework tasks. To simplify analysis and interpretation of the dietary data, categorical CDQ subscales were converted into binary variables using thresholds that approximately balanced the number of responses in each category.

Changes in outcome variables were compared between intervention and control groups using generalised linear mixed models (GLMMs) that adjusted for fixed and random (subjects nested in schools) effects. This approach was taken to account for the intra-school correlations and intra-person correlations between the repeated measures. Outcomes were controlled for baseline measures and age. A gamma probability distribution and an log link function was applied for most outcome variables, the exception being weekday TV, which were evaluated using a Gaussian distribution and identity link function. Models predicted outcomes at completion of the intervention (T_1_). Additional models predicted outcomes at 6 months post intervention (T_2_). Marginal effects were evaluated on select variables. Descriptive analyses were conducted in IBM SPSS Statistics v23 (IBM Cooperation, USA) and GLMM’s were carried out using Stata V14 (Stata Corporation, USA).

## Results

**Fig. 1:**
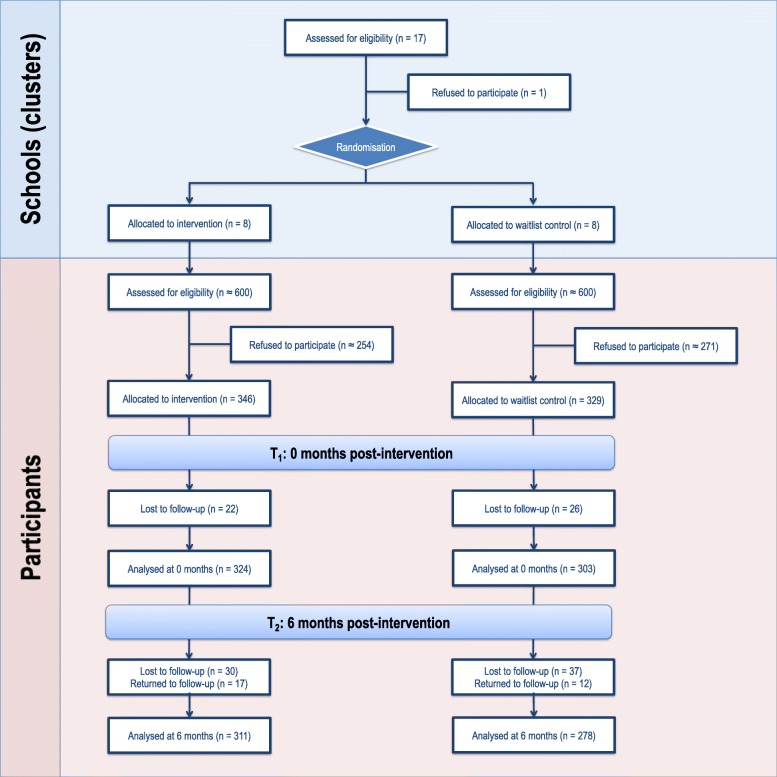
Participant allocation and retention

The Healthy Homework intervention was conducted between August 2011 and August 2012. Figure 1 depicts participant allocation and retention across each phase of the study in the form of a CONSORT flow diagram. A total of 675 children (intervention = 171 boys and 175 girls; control = 155 boys and 174 girls) aged 7–10 years provided written parental consent and assent to participate in the evaluation (consent rate = 56.3%). Cluster size (i.e., the number of children from each school) ranged from 14 to 53 (median [IQR] = 47.5 [36.5, 50]). Absent days, changing school, and withdrawals resulted in 46 children that did not participate in the first follow up (T_1_), and 67 children that did not participate in the second follow-up 6-months post intervention (T_2_). However, 29 of the 46 children absent from T_1_ returned for T_2_. The completed CONSORT checklist can be accessed as an Additional file 1.

Table 1 shows the descriptive characteristics of the study sample. There were no meaningful differences in age, gender distribution, or physical activity between the intervention and control groups at baseline. However, the intervention group contained notably more participants from schools in lower SES areas, had a higher proportion of children from Maori and Pacific backgrounds, as well as slightly higher BMI, WHtR, and daily servings of vegetables. Overall, the sample averaged 5090 ± 2200 steps/day (mean ± SD) at home on weekdays, 5410 ± 2200 steps/day at school, and 7440 ± 4100 steps/day on weekends, with boys more active than girls. No systematic pattern emerged with regard to the missingness of the data. For outcomes reported by proxy questionnaire, the response rate at T3 averaged between 54 and 60%. We removed weekend activity from our secondary analyses because the response rates were below 50%. All other outcomes evaluated – BMI, weekday activity – had a response rate in excess of 70%.

**Table 1:** Descriptive characteristics of intervention and control groups at baseline

Variable	Intervention group (*n* = 346)	Control group (*n* = 329)
Demographics		
Age (y)	8.71 ± 0.987	8.74 ± 1.04
Sex		
Males	171 (49.4%)	155 (47.1%)
Females	175 (50.6%)	174 (52.9%)
Ethnicity		
European	228 (65.9%)	232 (70.5%)
Maori	36 (10.4%)	18 (5.5%)
Pacific Island	22 (6.4%)	6 (1.8%)
Asian	46 (13.3%)	63 (19.1%)
Other	14 (4.0%)	10 (3.0%)
School SES decile		
1–3	50 (14.5%)	0 (0%)
4–7	116 (33.5%)	162 (49.2%)
8–10	180 (52.0%)	167 (50.8%)
Body composition		
Height (cm)	132 ± 7.17	132 ± 8.00
Weight (kg)	30.8 ± 7.45	30.1 ± 6.44
BMI (kg.m^−2^)	17.4 ± 2.82	17.0 ± 2.39
Waist circumference (cm)	61.5 ± 8.34	60.3 ± 7.40
Waist-to-height ratio	0.465 ± 0.052	0.455 ± 0.049
Physical (in)activity		
Weekday PA: School (steps.day^−1^)	5360 ± 2100	5450 ± 2310
Weekday PA: Home (steps.day^− 1^)	5000 ± 2060	5150 ± 2330
Weekend PA (steps.day^− 1^)	7570 ± 4320	7240 ± 3800
Weekday TV (hours.day^− 1^)	1.00 (0.480, 1.66)	1.00 (0.440, 1.49)
Weekend TV (hours.day^− 1^)	2.00 (1.00, 2.65)	1.65 (1.00, 2.50)
Weekday computer (hours.day^− 1^)	0.200 (0, 0.600)	0.200 (0, 0.440)
Weekend computer (hours.day^− 1^)	0.513 (0, 1.15)	0.425 (0.100, 1.00)
Dietary patterns		
Daily fruit consumption		
< 2 servings	64 (21.6%)	72 (24.7%)
2–3 servings	166 (56.1%)	169 (57.9%)
4+ servings	66 (22.3%)	51 (17.5%)
Daily vegetable consumption		
< 2 servings	109 (36.7%)	129 (44.3%)
2–3 servings	149 (50.2%)	138 (47.4%)
4+ servings	39 (13.1%)	24 (8.2%)
Weekly fast food consumption		
None	129 (43.3%)	130 (44.8%)
Once	125 (41.9%)	126 (43.4%)
More than once	44 (14.8%)	34 (11.7%)
Weekly soft drink consumption		
None	109 (36.6%)	119 (40.6%)
Once	56 (18.8%)	46 (15.7%)
More than once	133 (44.6%)	128 (43.7%)

Categorical data are presented as n (%), and continuous data are presented as mean ± SD or median (IQR) where appropriate. School socioeconomic decile was sourced from NZ Ministry of Education records

**Fig. 2:**
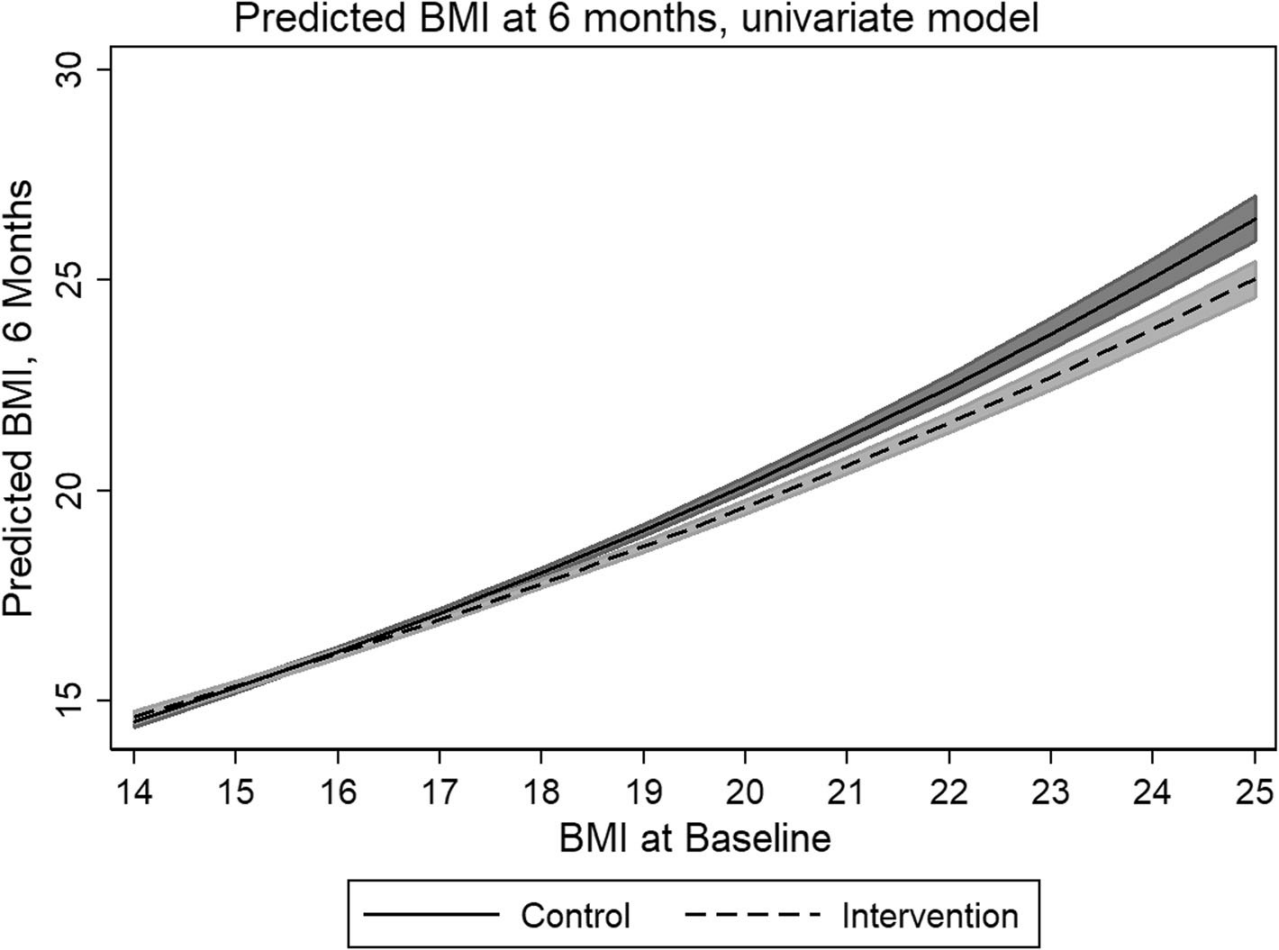
Predicted difference in BMI between study groups (univariate model)

Table 2 presents the results for the GLMMs. In general, the intervention group did not experience a statistically significant reduction in BMI immediately after the completion of the intervention (p: 0.755), but did demonstrate a statistically significant reduction in BMI at 6 months post intervention (p: 0.020). The predicted marginal mean of BMI in the control group at 6 months was 17.451; the intervention group, 17.274. However, marginal analyses revealed that those subjects that are likely to benefit most are students that have a baseline BMI of 17 or higher (Fig. 2), with a statistically significant difference between the intervention and control groups emerging at this point (β: − 0.267, z: − 2.19, p: 0.028, CI: − 0.017 − 0.001).

**Table 2:** Generalized linear mixed model results of between group differences, T_1_ and T_2_, primary and secondary outcome variables

Outcome^a^	β	SE	Z	*P*	95% CI
Body size					
BMI - T_1_	-0.001	0.004	-0.31	0.755	-0.007, 0.007
BMI - T_2_	-0.010	0.004	-2.32	0.020*	-0.019, 0.002
WtHR - T_1_	-0.030	0.021	-1.41	0.159	-0.072, 0.012
WtHR - T_2_	0.001	0.011	0.10	0.921	-0.020, 0.022
Physical Activity					
Weekday steps, school - T_1_	0.124	0.070	1.77	0.077	-0.014, 0.262
Weekday steps, school - T_2_	0.106	0.071	1.48	0.139	-0.034, 0.246
Weekday steps, home - T_1_^b^	0.260	0.070	3.62	< 0.001*	0.078, 0.395
Weekday steps, home - T_2_^b^	0.194	0.083	2.35	0.019*	0.032, 0.356
Weekend steps - T_1_	0.304	0.065	4.65	< 0.001*	0.176, 0.431
Weekend steps - T_2_	0.420	0.119	3.53	< 0.001*	0.187, 0.653
Nutrition and Television					
Fruit consumption - T_1_	0.207	0.099	2.10	0.036*	0.014, 0.401
Fruit consumption - T_2_	0.017	0.134	0.13	0.900	-0.247, 0.281
Vegetable consumption - T_1_	0.042	0.112	0.38	0.707	-0.179, 0.263
Vegetable consumption - T_2_	0.153	0.136	1.12	0.263	-0.115, 0.420
Fast food consumption - T_1_	0.003	0.064	0.05	0.958	-0.121, 0.128
Fast food consumption - T_2_	0.127	0.100	1.27	0.203	-0.068, 0.323
Soft drink consumption - T_1_	0.037	0.131	0.28	0.780	-0.220, 0.294
Soft drink consumption - T_2_	-0.007	0.174	-0.04	0.969	-0.347, 0.334
Weekday TV- T_1_	0.048	0.077	0.62	0.536	-0.104, 0.200
Weekday TV- T_2_	0.009	0.099	0.09	0.925	-0.184, 0.203
Weekend TV- T_1_	-0.032	0.128	-0.25	0.802	-0.284, 0.220
Weekend TV- T_2_^b^	-0.005	0.173	-0.03	0.978	-0.343, 0.334

*P < 0.05

^a^Random effects statistics excluded for brevity, but available upon request. All models were adjusted for age and sex

^b^Crude results presented in this table. Adjusted models, controlling for school socioeconomic decile presented in Table 3

Overall activity increased at T_1_ and T_2_ time points. This increase was statistically significant for weekday home and weekend activity (Table 2). School decile had exhibited a statistically significant impact on weekday home activity at T_1_, with the greatest improvements in weekday home activity occurring in the most socioeconomically deprived schools (Table 3). This increase persisted into T_2_, albeit attenuating to just outside of statistical significance (Table 3). Weekend television watching increased relative to baseline in those subjects from the most socioeconomically deprived schools at T_2_, and this difference was statistically significant (Table 3).

**Table 3:** Outcome measures effected by school socioeconomic decile

	β	SE	Z	*P*	95% CI
Weekday steps, home - T_1_	0.211	0.633	3.33	0.001	0.087, 0.335
Reference: school decile 8–10	-	-	-	-	-
Deciles 4–7	0.161	0.064	2.52	0.012	0.036, 0.286
Decile 3	0.326	0.136	2.39	0.017	0.059, 0.593
Weekday steps, home - T_2_	0.168	0.075	2.24	0.025	0.012, 0.314
Reference: school decile 8–10	-	-	-	-	-
Deciles 4–7	0.089	0.075	1.18	0.239	-0.059, 0.237
Decile 3	0.298	0.159	1.88	0.060	-0.013, 0.609
Weekend TV - T_2_	-0.098	0.124	-0.79	0.431	-0.341, 0.145
Reference: school decile 8–10	-	-	-	-	-
Deciles 4–7	0.144	0.149	0.097	0.334	-0.148, 0.436
Decile 3	1.303	0.149	18.11	< 0.001	1.162, 1.444

**Fig. 3:**
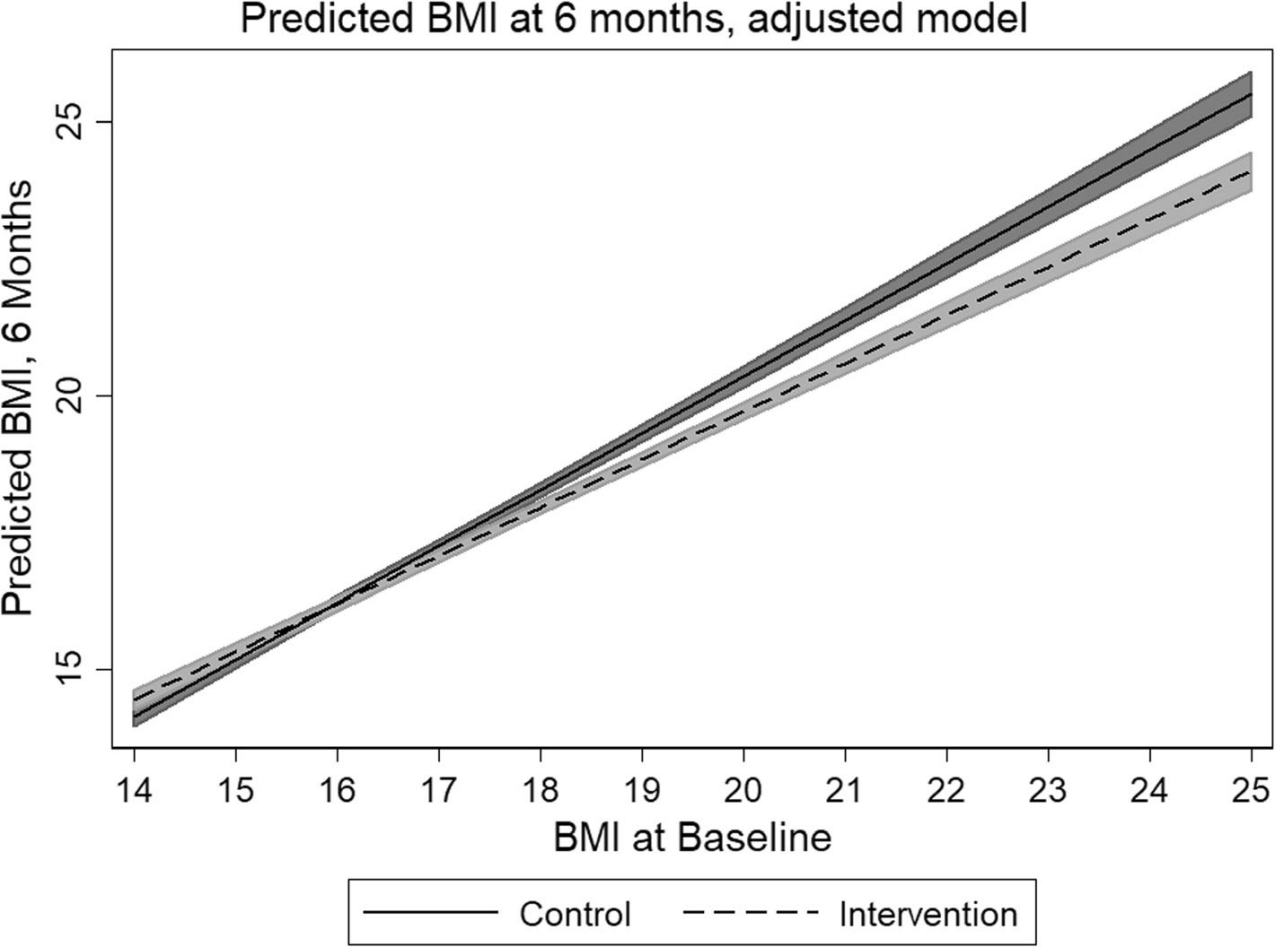
Predicted difference in BMI between study groups (adjusted model)

The adjusted model evaluating covariate effect on BMI at T_2_ revealed that weekday home activity was the most significant covariate (Table 4 and Fig. 3). For each increase in 1000 steps, an almost 0.6 point decrease in BMI was realized. This model was adjusted for school decile. These decreases were most pronounced in the socioeconomically most deprived schools, where subject BMI decreased 0.476 points per 1000 steps, when controlling for other covariates. Proxy-reported vegetable consumption at T_2_ was the second most important predictor of BMI reduction at T_2_, where each meal of vegetable consumed resulted in a 0.036 point decrease in BMI. It should be noted that weekend steps could not be evaluated with respect to school decile or BMI at T_2_, because only 39 and 47% matching observations were available (respectively).

**Table 4:** Adjusted model, BMI-T_2_

	β	SE	Z	*P*	95% CI
Intervention	-0.110	0.085	-1.29	0.198	-0.277, 0.057
Weekday steps, home - T_2_	-0.059^a^	0.017	-3.57	< 0.001	-0.277, -0.026
Decile					
Reference: school decile 8–10	-	-	-	-	-
Deciles 4–7	0.100	0.087	1.16	0.246	-0.070, 0.271
Decile 3	-0.477	0.204	-2.33	0.020	-0.877, -0.271
Vegetables consumed - T_2_	0.036	0.033	-1.09	0.275	-0.099, 0.028

^a^Reflects slope per 1000 steps taken

## Discussion

In this study, we evaluated the efficacy of the Healthy Homework programme for improving physical activity, dietary patterns, and body size in primary-aged children. Despite being a school-based intervention, a key strength of this study was the design of a homework syllabus that maximised family participation and engagement, thereby targeting out-of-school behaviours. Our results demonstrate significant and sustained increases in physical activity 6-months post-intervention. Of particular note were the large effects on out-of-school physical activity, approximate to hypothetical increases of 15.6 and 29.7% (i.e., intervention effects compared to baseline values) each weekday and weekend day, respectively. This degree of improvement, should it persist over the long term, would likely have a meaningful impact on children’s health and wellbeing: reviews have identified favourable effects on a wide range of physical, psychosocial, and cognitive outcomes of even modest increases in physical activity [33, 34]. Furthermore, the relatively larger effects observed in children from the most socioeconomically-deprived schools suggest that this intervention approach may be an effective way of engaging at-risk populations.

Significant intervention effects on BMI and fruit consumption were also observed, although the effects were smaller and inconsistent across time points. Intervention effects on BMI, in particular, were limited. No difference was observed at the end of the intervention, and although a statistically significant difference was observed between groups at T2, such a difference would not be deemed clinically relevant. It is possible that the follow-up time was insufficient to allow any improvements in physical activity to influence body size. Nonetheless, our findings provide some support for the development and implementation of resources that enhance family involvement in health-related curriculum areas.

Despite the positive effects for physical activity, we did not observe any meaningful changes in screen time or dietary patterns. The lack of effect on screen time may be explained by the relatively minor emphasis placed on this topic (only one session); however, half the module featured advice and activities relating to nutrition and diet. We did note via anecdotal feedback that the children appeared to prefer the physical activity topics over the nutrition topics, and perhaps this played a role in the lack of consistent dietary effects. It is possible that physical activity is a more malleable and less complex behaviour than diet in children [35], or that our questionnaire-based assessment of dietary intake was not sensitive enough to quantify meaningful changes in this sample. In any case, this study suggests that nutrition initiatives in primary schools may not achieve the same level of benefits as similar physical activity programmes.

One of the distinctive aspects of the intervention was its compulsory nature, with a number of key health messages grounded in the national school curriculum [29]. Although there is promising evidence that purely family-based intervention approaches can improve children’s physical activity and diet [36, 37], teaching nutrition and physical activity within the primary-school curriculum provides a cost-effective method of reaching large numbers of children without the requirement of children or families to ‘opt-in’. Given the significant effects on physical activity, body size, and fruit consumption in this study, it appears that curriculum-based, health-related programmes in primary schools can have an impact on the behaviours of children, at least over the short-medium term.

The significant improvements observed in the present study are in contrast to the lack of effects reported by the AFLY5 study [25, 26], which featured an intervention design very similar to the present study (both studies targeted similar lifestyle behaviours via compulsory in-class lesson plans and homework tasks). The AFLY5 authors offered several explanations as to why AFLY5 may not have had a significant impact, such as the delay in time between intervention development and implementation, and the potential need for more intensive behavioural change and/or upstream societal or environmental approaches. None of these possibilities, however, explain the difference in outcomes between AFLY5 and Healthy Homework. One clear distinction was that Healthy Homework included a minimum of 14 and a maximum of 42 homework tasks over the 8-week period, whereas AFLY5 provided 10 parent-child interaction activities. Furthermore, AFLY5 researchers reported difficulties motivating some teachers, who felt the need to prioritise literacy and numeracy above health-promoting lessons [26]. Another similar intervention – the Eat Well and Keep Moving Program – was conducted over a two-year period, and integrated materials designed to increase physical activity, improve dietary patterns, and reduce television viewing within the grade 4 and 5 school curriculum (including homework components) [11]. The evaluation of the intervention revealed significant improvements in dietary patterns and a marginal decrease in television viewing via 24-h recall questionnaires. The present findings add to this body of evidence by demonstrating the potential for positive physical activity outcomes when using objective measurement techniques; however, the effects of embedding health-related lessons and homework within school curricula appear to be variable, and may depend on the specific population and/or context.

There were several limitations present in this study that should be acknowledged. While we were able to collect data about the overall amount of physical activity, we were not able to clarify with any detail the within-day patterns and intensity of activity. Pedometers were primarily chosen for financial reasons; however, the use of omnidirectional accelerometers would have enabled another level of analysis. Related to this point, we were not able to determine the effects on sedentary behaviour, which cannot be effectively isolated via pedometry. As mentioned earlier, a more comprehensive dietary assessment may have revealed some effect on nutrition, although the substantial response burden may have resulted in participant bias. The four items from the CDQ, selected due to their alignment with NZ policy, may not exhibit the same levels of validity and reliability as the full scales. In addition, generalisability of conclusions based on the data in this study is limited by the selection of participants from only two regions of New Zealand; the study design clearly requires replication in larger and more diverse populations. Socioeconomic decile ratings were not evenly matched by intervention and control group; there was one Decile 1 intervention school, whereas the lowest decile rating in the control group was Decile 3. Another limitation was that the research assistants collecting data were not blinded to the school allocation, which may have introduced accidental or deliberate bias during anthropometric assessment. Finally, intervention fidelity, which would have enabled per protocol analysis, was not assessed.

## Conclusions

In summary, we have shown that a curriculum-based health programme can result in consistent improvements to physical activity levels in primary school-aged children, with significant but limited effects on body size and fruit consumption. The next step is to see if such programmes are sustainable within the school environment over a long term, and without input from researchers. The effects of such programmes on academic and neurocognitive outcomes development is also of interest and should be explored in future.

## Additional file

Additional file 1: CONSORT 2010 checklist of information to include when reporting a randomised trial. (DOC 218 kb)

Additional file 2: Teachers Manual. (PDF 11980 kb)

Additional file 3: Students Manual. (PDF 10554 kb)

## Data Availability

The datasets generated and/or analysed during the current study are not publicly available due to a confidentiality agreement with participating schools. Enquiries should be addressed to the principal author.

## Abbreviations

AFLY5: Active for life year 5
BMI: Body mass index
CDQ: Children’s dietary questionnaire
GLMM: Generalised linear mixed model
RCT: Randomised controlled trial
T_0_, T_1_, T_2_: Baseline, first follow-up, and second follow-up time points
WHtR: Waist-to-height ratio
