# Supplementary material for: Efficacy of a compulsory homework programme for increasing physical activity and improving nutrition in children: a cluster randomised controlled trial
Source: Int J Behav Nutr Phys Act. 2019 Sep 5;16:80. doi: 10.1186/s12966-019-0840-3 (PMC6729097; doi:10.1186/s12966-019-0840-3)
Supplement: Supplementary file 2 — Teachers Manual. (PDF 11980 kb) [file 12966_2019_840_MOESM2_ESM.pdf]

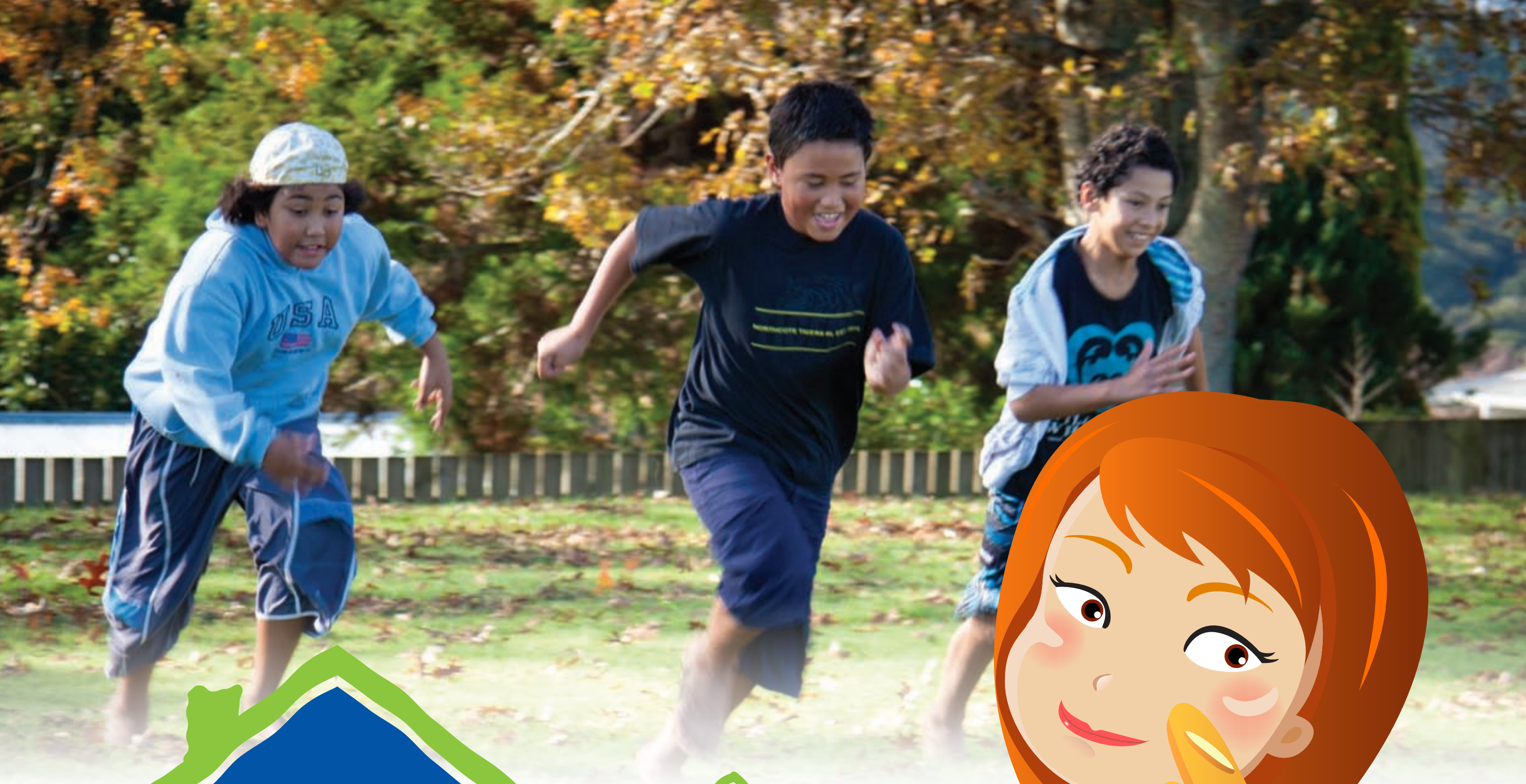

Healthy Homework

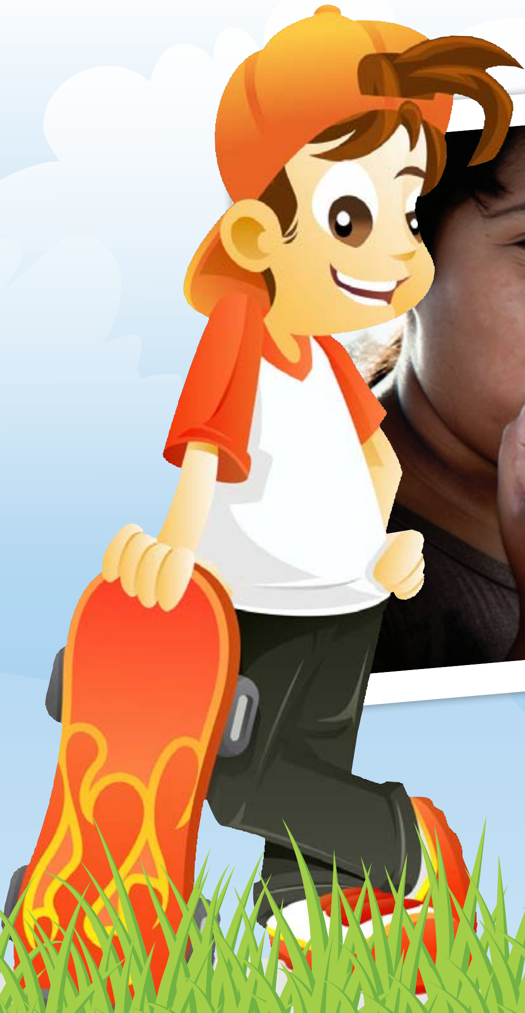

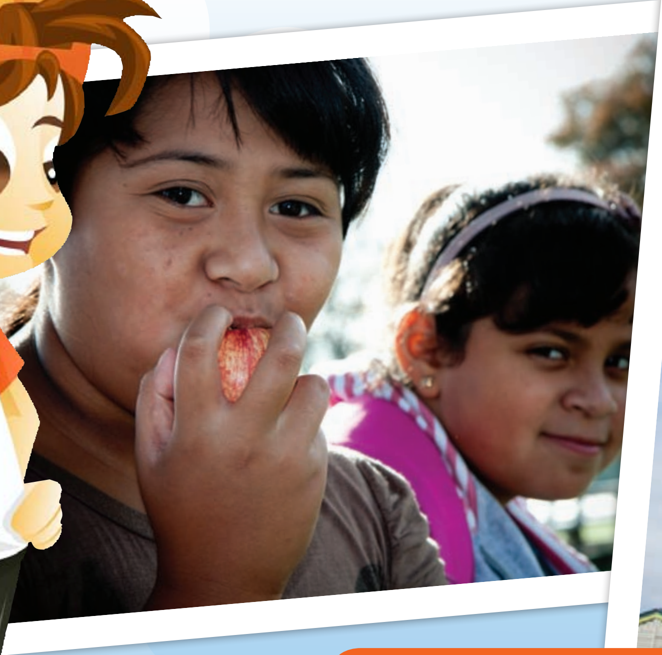

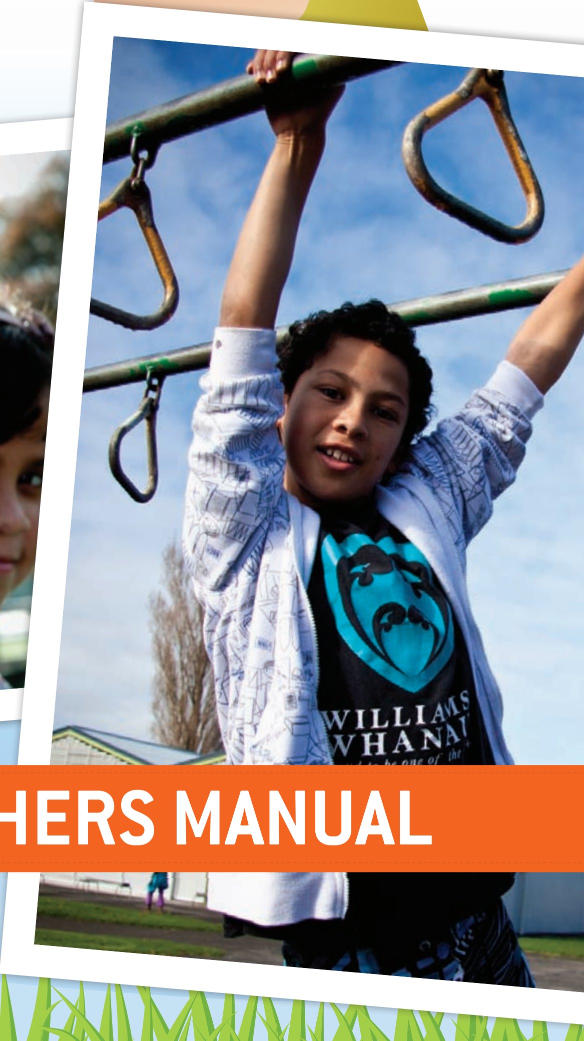

TEACHERS MANUAL

Proudly supported by AUT UNIVERSITY

CONTENTS

Background **3**
Acknowledgements **3**
Structure of the Resource **4**
- In-Class Teaching Unit 4
- Homework Module 4
- Website 5
- Student Profiles 5
- Incentives 5
- Final Assessment Task 6
- Links to the New Zealand School Curriculum 6
- Guideline for Learning 7

Week One **10**
- Session 1: Let's Learn About Healthy Homework 11
- Session 2: Walking 12
- Session 3: Foods We Eat 14

Week Two **17**
- Session 1: Homework Review 18
- Session 2: Screen Time 18
- Session 3: Energy Foods 22

Week Three **24**
- Session 1: Homework Review 25
- Session 2: Sports 25
- Session 3: General Nutrition 27

Week Four **30**
- Session 1: Homework Review 31
- Session 2: Games 31
- Session 3: Fruit and Vegetables 33

Week Five **35**
- Session 1: Homework Review 36
- Session 2: Fitness 36
- Session 3: Drinks 38

Week Six **41**
- Session 1: Homework Review 42
- Session 2: Dance and Movement 42
- Session 3: Breakfast, Lunch, and Dinner 44

Week Seven **47**
- Session 1: Homework Review 48
- Session 2: Confidence and the Outdoors 48
- Session 3: Snacks and Takeaways 50

Week Eight **54**
- Session 1: Homework Review 55
- Session 2: Healthy Homework Presentations 55

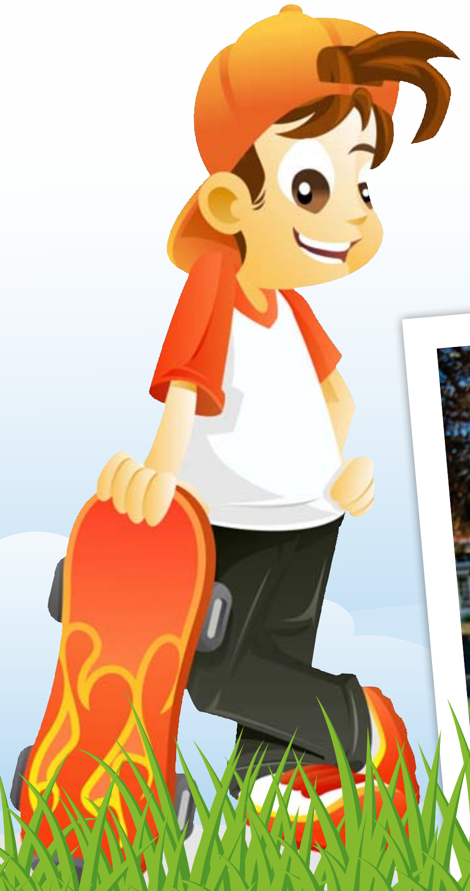

BACKGROUND

Physical activity and good nutrition in children are key priorities for New Zealand's health and education sectors. While most health promotion interventions for young people focus on the school setting, evidence suggests that children are less active and have greater access to unhealthy foods outside of school. Healthy Homework is an initiative that aims to bridge this gap by promoting and encouraging children to be active and eat well in their home environment.

ACKNOWLEDGEMENTS

Healthy Homework was developed by a multi-disciplinary team at AUT University. The project team has also received advice from a steering group representing the following organisations:

- Edgar National Centre for Diabetes Research Otago University
- NZ Principals Association
- Physical Education New Zealand (PENZ)
- Centre for Physical Activity and Nutrition, AUT University
- School of Arts, Development, and Health Education, Massey University
- Te Whare Wananga o Awanuiarangi

The Healthy Homework Team would like to acknowledge the ongoing support of senior management from all participating primary schools in both the Auckland and Otago regions.

The Healthy Homework team would like to acknowledge the Health Research Council (10/207) for their funding and support.

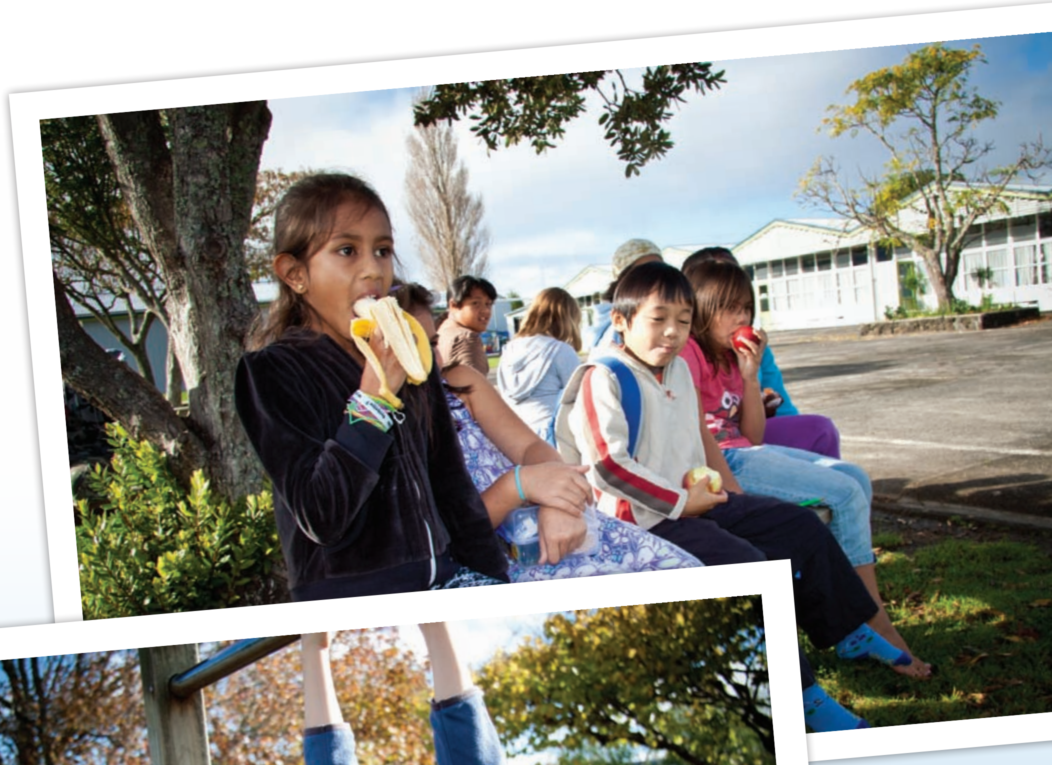

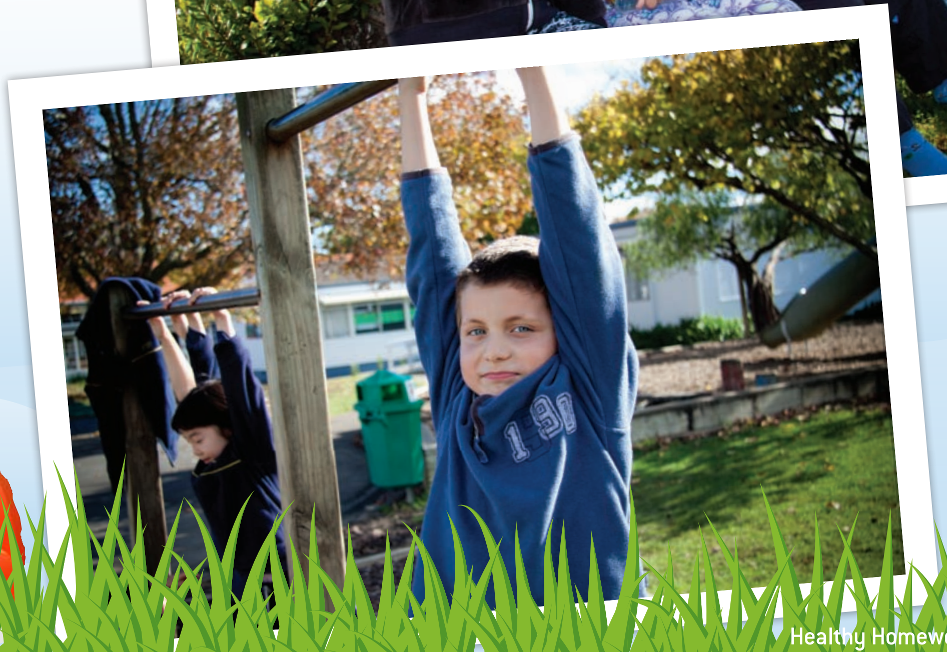

STRUCTURE OF THE RESOURCE

Healthy Homework comprises of an eight-week teaching/homework programme. Aligning with the New Zealand Curriculum, Healthy Homework promotes student learning through effective inquiry based pedagogy, encouraging reflective thought and action around the concepts of healthy eating and physical activity.

This resource includes a teaching guide, a homework booklet, and materials to support individual sessions.

IN-CLASS TEACHING UNIT

The Healthy Homework teaching unit is designed to encompass three 1½-hour sessions per week for seven weeks plus one final wrap-up session. The content and suggested learning activities for each session are provided in the teaching guide for teachers to select from.

In line with the New Zealand Curriculum, it is proposed that teachers will adapt this programme:

- To meet the specific learning needs of the children in their class (children's current physical activity levels and knowledge and practice of healthy eating)
- By drawing on children's prior learning and experiences
- By encouraging conversations with other people (including family members and people in the wider community)
- By making connections across other learning areas

The teaching guide is formatted as follows:

- Links to the curriculum
- Session 1: Ideas for reviewing the prior week's homework
- Session 2: Physical activity learning activities
- Session 3: Food and nutrition learning activities.

PLEASE NOTE: If more support information for specific topics is required, refer to the links on the Healthy Homework website.

HOMEWORK MODULE

This resource asks that children complete at least one of three physical activity options and one of three nutrition options from their booklets that reflects the learning topic/focus for that week. The homework component of this programme is integral to reinforcing learning and meeting the aims of this unit.

PLEASE NOTE: Teachers are encouraged to adhere to their schools homework policy whilst keeping in mind the aims of this programme.

Incentives will be provided based on the number of weekly tasks completed (see Incentives page).

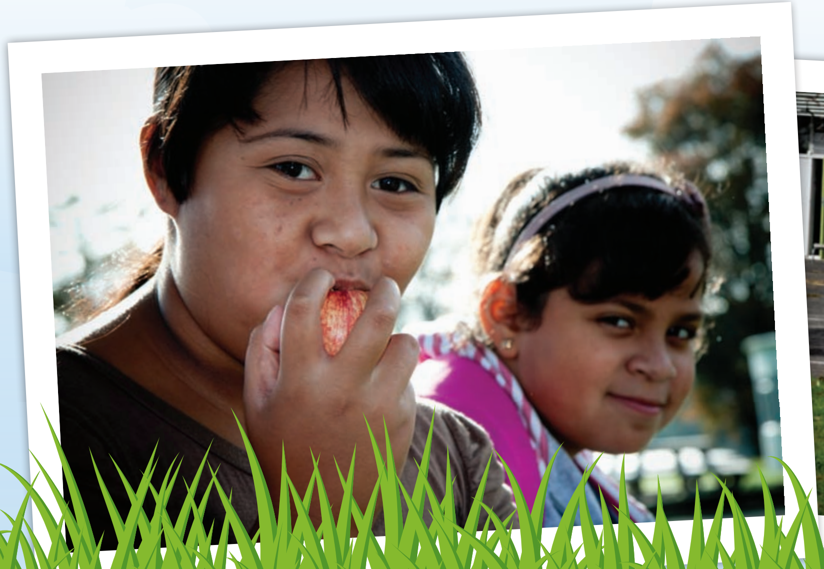

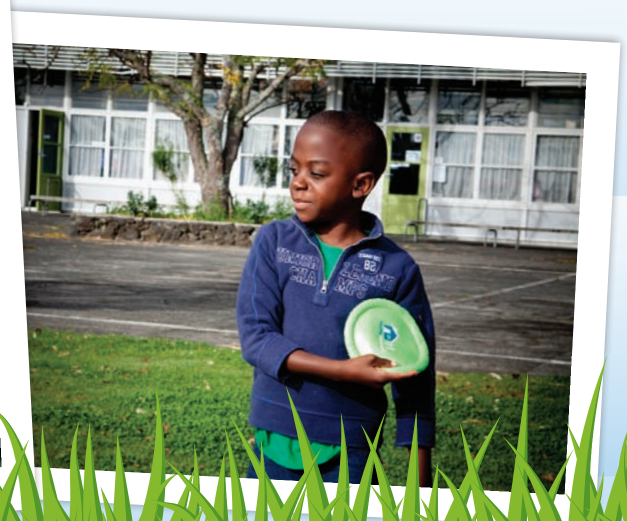

WEBSITE

A website has been developed to complement the Healthy Homework teaching unit. The website allows children:

- To set up their own profiles
- To create blogs for communicating with children in their own school and other participating primary schools
- To post photographs and reflections throughout the eight weeks of the Healthy Homework programme.

PLEASE NOTE:

- The Healthy Homework website is not a public website and can only be accessed by the children and teachers who are involved with the Healthy Homework programme.
- Teachers and children will be issued with a handout with instructions on how to enter student data and access information and resources on weekly topics.

STUDENT PROFILES

Each student will need to log on to the Healthy Homework website to create a profile for themselves. This will allow the teacher access to student postings that align with homework tasks and to complete the marking schedule.

PLEASE NOTE:

- Teachers will have administrative rights to edit blogs if necessary.
- The AUT research team will be monitoring the site daily for inappropriate content.

INCENTIVES

Each class will be allocated Healthy Homework wristbands in four different colours. After the first week's homework has been reviewed, the wristbands can be distributed to the children as they attain a certain level of homework achievement. Wristbands can be distributed at the beginning of the week when all homework from the previous week has been marked.

Wristband colours are as follows:

- White (lowest level)
- Bronze
- Silver
- Gold (highest level)

The more homework a student completes, the higher the level of colour wristband they will qualify for. Children are encouraged throughout the Healthy Homework programme to post blogs and photos, gaining points by doing so and helping to achieve a higher colour of wristband. To achieve the gold wristband, children must have completed all homework tasks as well as posted a certain number of blogs and photos.

Children are encouraged to finish off tasks from previous weeks, even if you have moved forward several topics. Once you mark a student's completed activities from previous weeks, the website will automatically add this to the child's overall score and indicate which band they have qualified for.

PLEASE NOTE:

- Many children who are already active with existing sporting commitments may find it difficult to complete all activities. Teachers are encouraged to use their discretion in deciding when some homework activities could be covered by children's other sporting activities.
- Consideration should be given for children who cannot meet all homework tasks, e.g. do not have access to a television or whose family members are unable to support them in completing tasks. In these cases, homework tasks will need to be modified.

Children will also receive a tick in their homework booklet for each homework task they complete. As you mark a child's homework, tick the space provided to indicate that you have marked this task on the Healthy Homework website.

FINAL ASSESSMENT TASK

In small groups, children choose an aspect of the Healthy Homework programme. Following the inquiry learning model sequence outlined below, groups report their learning from the Healthy Homework programme back to the class.

PLEASE NOTE:

- Assessment activity to be introduced at the start of the programme.
- Session 1 will focus on ascertaining children's prior knowledge and experience on the topics of physical activity and healthy eating.

1. What did we already know prior to starting this unit about physical activity and healthy eating?
2. What did we want to know and practice to further our learning?
3. What did we learn, practice, or take action on in the teaching programme?
4. What aspects of this learning or practice will we continue within our daily lives?

Groups decide on how they want to present their learning to the rest of the class, suggestions may include; PowerPoint presentations, plays, posters, documentaries, news reports, videos, gardens, advertisements, or any other fun way the children come up with to convey their information and learning to the rest of the class.

Suggested trigger questions to encourage thinking:

- What activities did they learn most from?
- Was there anything that they think could have been included to improve the Healthy Homework programme?
- Reflect on what Healthy Homework meant to you, your family, and your friends.

LINKS TO THE NEW ZEALAND SCHOOL CURRICULUM

This resource has been designed to support teaching and learning in "Health and Physical Education (HPE): in the New Zealand Curriculum". It focuses on the key areas of learning of Physical Activity and Food and Nutrition. The Healthy Homework teaching/learning unit is designed to support children's learning about the lifelong benefits of regular physical activity and healthy eating.

- The Healthy Homework activities and teaching unit are designed to complement each other and help children to identify factors that influence their personal health, the health of their whanau, community, and environment and promote interaction between these areas.
- The Healthy Homework resource acknowledges the social and cultural differences in communities and is also planned with the intention that schools will adapt activities and ideas to work alongside existing school and community events.
- The Healthy Homework programme encourages children to research and describe current and traditional health trends and to attempt to share relevant findings and transfer positive practices to their home and family life.

➲ KEY COMPETENCIES

- **Participating and contributing:** to participate and contribute actively both individually as well as with peers, with family, or community members to promote the benefits and practice of physical activity and healthy eating.
- **Managing self:** to engage in a range of learning experiences which encourage children to make informed choices and act independently for healthy lifestyle practices.
- **Thinking:** to construct knowledge, reflect on and evaluate influences that affect food choices and participation in regular physical activity.
- **Relating to others:** to share learning, value diversity, and negotiate to achieve desired outcomes.
- **Using language, symbols, and texts:** to access and interpret new knowledge and to communicate ideas.

➲ HEALTH AND PHYSICAL EDUCATION – INTERDEPENDENT CONCEPTS

- **Hauora:** develop an understanding of the relationship between regular physical activity and healthy eating on personal wellbeing.
- **Socio-ecological:** explore factors that influence our choice and enjoyment of differing foods and physical activities.
- **Attitudes and values:** developing a responsible attitude to their own wellbeing.
- **Health promotion:** develop knowledge and implement plans to foster sustainable healthy lifestyle practices.

Four strands with the curriculum

Health and Physical Education Curriculum

Strand A - Personal Health & Physical Development	Strand B - Movement Concepts & Motor Skills	Strand C - Relationships with Other People	Strand D - Healthy Communities and Environments
Personal growth & development, A1	Movement skills, B1	Relationships, C1	Societal attitudes and beliefs D1
Regular physical activity, A2	Positive attitudes & challenge, B2	Identity, sensitivity & respect, C2	Community resources, D2
Safety risk management, A3	Science & technology, B3	International skills, C3	Rights, responsibilities, laws, D3
Personal identity & self-worth, A4	Social cultural factors, B4		People and the environment, D4

PLEASE NOTE: Learning in the contexts of physical activity and healthy eating may occur over many of the achievement objectives (AO's) outlined in the learning area of Health and Physical Education. In this resource, possible learning objectives and intended outcomes that relate to physical activity and healthy eating are outlined at the beginning of each session. The suggested success criteria could be used to guide discussions between teachers and students about what the learning may look like. In this resource, learning is directed to level 3 of the curriculum; however to ensure effective and focused learning, teachers are encouraged to adapt achievement objectives to appropriate levels in line with student learning needs.

Key messages:

1. Physical activity and healthy food choices contribute to wellbeing.
2. Active living (e.g., walking, raking leaves, carrying groceries), active recreation (e.g., play, skateboarding, bike riding, dance), and active organised sport (e.g., school sport, club) are forms of physical activity that contribute to wellbeing.
3. To be active and healthy, food is needed to provide energy for the body.
4. A healthy diet is made up from a variety and balance of different foods and drinks.
5. A wide range of factors influences physical activity and healthy eating choices.
6. Being physically active and eating with others can strengthen self-worth, friendships, families, and communities.

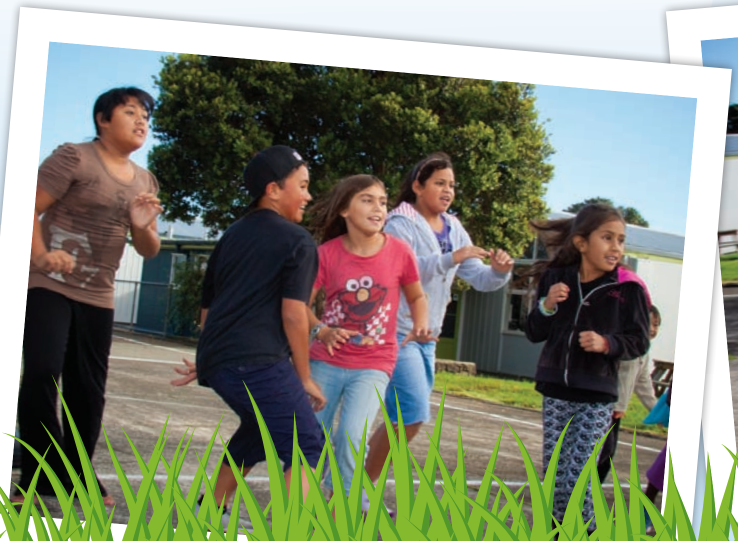

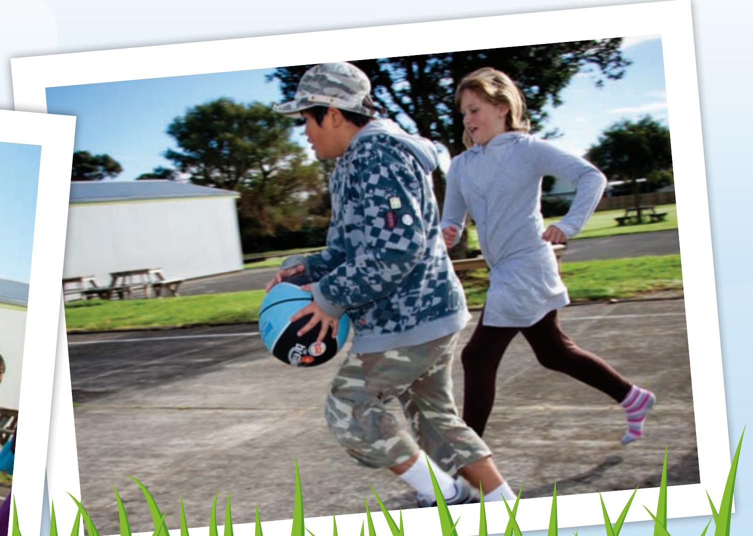

Week	Session	Topic	Strand and Achievement Objectives (AO's)	Intended Learning Outcomes<br>We are learning to:	Resources Provided
One	Session 1	Introduction/ identifying prior knowledge	Personal Health and Development<br>3A1	Share ideas and learn more about how healthy eating and physical activity helps us to be healthy.	KWL worksheet
	Session 2	Walking	Personal Health and Development<br>3A2<br>Healthy Communities and Environments<br>4A3	Experience and describe the health benefits of walking.<br>Find out what makes it easy or difficult to go walking.<br>Identify hazards in different walking environments and think of ways we can keep others and ourselves safe.	Character cards<br>Group worksheet
	Session 3	Foods we eat	Healthy Communities and Environments<br>3D1<br>Personal Growth and Development<br>3A1	Identify factors that influence what people eat across the world.<br>Understand the different ways healthy food keeps us well.	Meal pictures<br>Interview sheet<br>Nutrient food cards<br>Seeds<br>Instructions for planting
Two	Session 1	Review			Bingo sheets
	Session 2	Screen time	Healthy Communities and Environments<br>3D1<br>Personal Health and Development<br>3A2	Explore and describe how screen time can influence what we eat and how active we are.<br>Set realistic and achievable physical activity goals to look after ourselves.	Weekly timetable<br>Advertising DVD<br>Advertising worksheet
	Session 3	Energy foods	Personal Health and Physical Development<br>3A2	Understand that the food we eat provides us with the energy we need to take part in physical activity.	Food energy cards<br>Activity energy cards
Three	Session 1	Review			
	Session 2	Sports	Movement Concepts and Motor Skills<br>3B4<br>Healthy Communities and Environments<br>3D1	Play an unfamiliar sport and describe how competition can affect how people play.<br>Identify how sport is influenced by culture and the environment.<br>Find and locate different sporting clubs in our local area.	Ki o rahi DVD<br>Ki o rahi instructions<br>Make the links worksheet
	Session 3	General nutrition	Personal Growth and Development<br>3A1	Understand the qualities of different foods so that we can make healthy balanced food choices.<br>Understand how healthy eating contributes to our wellbeing.	Pictures of food<br>Food diary<br>Shop for Your Life DVD
Four	Session 1	Review			Traffic light games
	Session 2	Games	Movement Concepts and Motor Skills<br>3B2<br>Relationships with Other People<br>3C1<br>Healthy Communities and Environments<br>3D1	Develop throwing and catching skills that give us confidence to make up and play games.<br>Be aware of people's differences and make sure anyone can join in and enjoy playing our games.<br>Identify how and why games change over time and differ between cultures.	Frisbees
	Session 3	Fruit and vegetables	Healthy Communities and Environments<br>3D1<br>Personal Health and Physical Development<br>3A1	Talk about food with others to create an environment that supports healthy food choices.<br>Ask questions about how much and what kinds of fruit and vegetables help make us healthy.	
Five	Session 1	Review			
	Session 2	Fitness	Personal Health and Physical Development<br>3A2<br>Movement Concepts and Motor Skills<br>3B3	Understand how fitness supports our health and our involvement in games and activities.<br>Describe our experiences in different physical activities.	Couple tag instructions<br>Activity cards<br>Group worksheet
	Session 3	Drinks	Personal Health and Physical Development<br>3A1	Understand why we need to drink regularly throughout the day.<br>Identify different kinds of drinks and how to make healthy choices.<br>Plan and take action to promote the drinking of water by children at school.	Drink labels<br>Traffic light worksheet
Six	Session 1	Review			Fizz Pop game instructions
	Session 2	Dance & Movement	Personal Growth and Development<br>3A2<br>Movement Concepts and Motor Skills<br>3B2	Participate in dance activities and understand how it contributes to our wellbeing.<br>Put a range of dance movements together and show others what we have learned.	Music/dance CD<br>Hauora worksheet
	Session 3	Breakfast, lunch, and dinner	Personal Growth and Development<br>3A1<br>Healthy Communities and Environments<br>3D2	Identify reasons why it is important to eat a healthy breakfast and lunch.<br>Help with planning and preparing meals and to describe how these activities contribute to family wellbeing.	Fibre cards
Seven	Session 1	Review			
	Session 2	Confidence and the Outdoors	Healthy Communities and Environments<br>3D1<br>Movement Concepts and Motor Skills<br>3B2	Find out what opportunities our local environment provides for outdoor physical activity.<br>Be confident and cooperative in challenging group adventure activities.	Problem solving activity cards
	Session 3	Snacks and takeaways	Personal Health and Physical Development<br>3A1	Be aware of different feelings in our bodies and make healthy choices to meet our physical needs.	Food labels<br>How to read labels information
Eight	Session 1	Review			
	Assessment		Personal Growth and Physical Development<br>3A1<br>Healthy Communities and Environments<br>3D1	Share experiences, understandings, and ideas about the effect of healthy eating and physical activity on wellbeing.<br>Identify and promote ways of sustaining or improving our wellbeing in these areas.<br>Demonstrate awareness of the things that influence our physical activity and what we eat.	

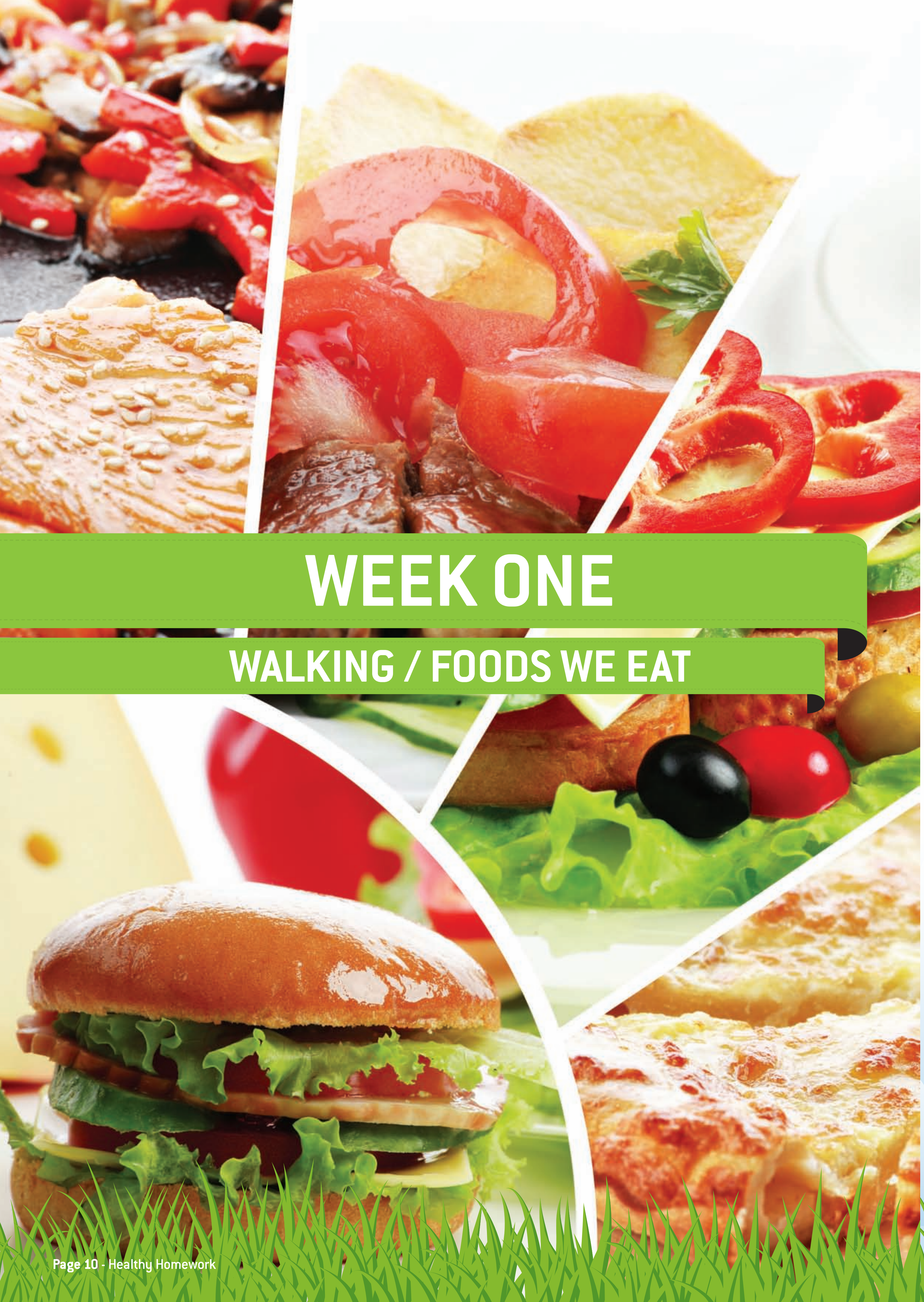

WEEK ONE

WALKING / FOODS WE EAT

WEEK ONE

SESSION 1: LET'S LEARN ABOUT HEALTHY HOMEWORK

CURRICULUM LINKS

Achievement Objective	Intended Outcome<br>We are learning to:	Success Criteria<br>We will know we have succeeded when we:
3A1<br>Personal Growth and Development	Share ideas and learn more about how healthy eating and physical activity helps us to be healthy.<br>Understand what the Healthy Homework unit is about.	Have created a mind-map showing our current understanding about the effect of healthy eating and regular physical activity on our wellbeing.<br><br>Can share three important points about the Healthy Homework programme with our parent/s or caregivers.

RESOURCES

What is Healthy Homework: Big sheets of paper
Assorted pens
KWL Worksheet: Worksheet

CLASSROOM ACTIVITIES

1. WHAT IS HEALTHY HOMEWORK?

Children talk to a partner about what they think is meant by "Healthy Homework". Possible questions to stimulate discussion:

- What does 'healthy' mean?
- What does it include?
- How do you know if you are healthy?
- Is healthy only when you are not sick?
- What affects someone's ability to be healthy?
- Is medicine the only solution if you are sick?
- What is homework?
- What is the point of homework?

Partners join with another pair and share their ideas.
On a big piece of paper, mind-map both 'physical activity' and 'healthy eating'. Include what they know, how they feel towards each of these things, factors that influence participation in physical activity, and factors that influence food choices.

LEARNING NOTES

- Homework supports learning in the classroom; as such it is a necessary component of this unit.
- Food and drinks = energy in. Sports, walking, jumping, etc. = energy out.
- Have children consider what they know about the physical, social, emotional, mental, and spiritual benefits of physical activity and healthy eating.
- Influences: television, sport, advertising, role models, seasonal foods, money, transport, etc.
- Physical activity and healthy eating contribute to a healthy lifestyle.

2. KWL WORKSHEET

Children fill in the first two columns from the KWL worksheet provided. This can then be completed at the end of the teaching/learning unit.

What I KNOW	What I WANT to know	What I have LEARNED

3. INTRODUCTION TO THE TEACHING/LEARNING UNIT

Share the structure of the teaching unit:

- Sessions
- Homework booklet (for explanation and recording of homework tasks)
- Final assessment task (gathering evidence throughout the weeks, e.g. photos, pictures, cuttings, notes, etc.).

(Keep small group brainstorming sheets to assist with final assessment task.)

LEARNING NOTES

- Reiterate focus on homework as a helpful tool.
- Discuss use of website and the setting up of their individual profile; emphasise the appropriate use of the online website.

Children write a letter home to their parents explaining the Healthy Homework unit and what they are looking forward to learning.

SESSION 2: WALKING

CURRICULUM LINKS

Achievement Objective	Intended Learning<br>We are learning to:	Success Criteria<br>We will know we are successful when we:
3A2<br>Regular Physical Activity	Experience and describe the benefits of walking.	We can say how walking and health are related.<br><br>We have taken part in at least three walking activities and explain which was the most fun and why.
D1<br>Societal Attitudes and Beliefs	Find out what makes it easy or difficult to go walking.	We can name common barriers to walking and develop possible solutions.<br><br>We can take part in walking activities with other family members and say how this felt different to walking on our own.<br><br>We compare and contrast our own experiences of walking with that of an elder and can think of things that influence people's physical activity levels.
4A3<br>Safety and Risk Management	Identify hazards in different walking environments and think of ways we can keep others and ourselves safe.	We can name dangers in different environments and make up rules and suggestions to keep people safe so they can enjoy walking.

RESOURCES

Step It Out: Map of school
Measuring tools
The Great Walk: Access to Google maps
Busting Barriers: Character cards
Group worksheet

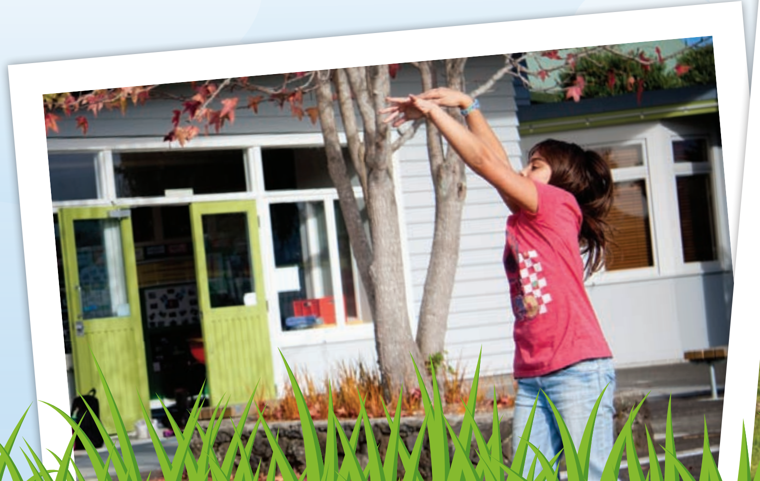

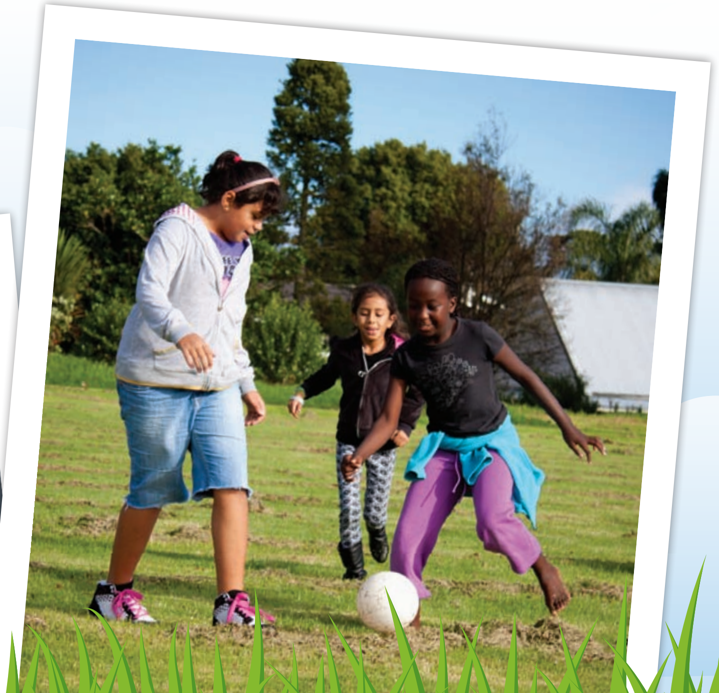

LEARNING NOTES

- Walking is the most common form of physical activity.
- Walking is a mode of transport.
- Health benefits of walking include brain development, bone strength, lower risk of diseases.
- Smaller bouts of walking have an accumulative health affect.
- A range of factors influences a person's motivation to walk, including the physical environment, personal and social barriers, and technology.
- Benefits of walking have a range of effects on social, mental/emotional, spiritual, and physical wellbeing, including family relationships, friendships, and participating within the neighbourhood environment.
- Children who walk independently become familiar with their surroundings and will feel more connected with their community.
- Walking is a simple and effective form of physical activity.

CLASSROOM ACTIVITIES

1. WHAT/WHY/HOW

As a class or in small groups, children think about and record ideas for the following:

- The reasons people may choose to walk, e.g. competition race-walking, to get somewhere, for exercise, when the car breaks down, for fun, or to be in the environment, tramping/hiking, exploring, to look for something, shopping.
- The potential benefits of walking. With the class list these ideas under the headings of hauora – social, mental/emotional, spiritual, and physical wellbeing.
- How to keep safe when walking in different environments, e.g. home, local community, city, and the great outdoors, e.g. bush, mountains, etc.

Key safety rules: (1) never walk alone at night or in areas that might be unsafe; (2) never talk to or get into a car with a stranger; (3) stay away from strange dogs; (4) always use proper road crossings on busy streets; and (5) never run when crossing the road.

2. STEP IT OUT

As recreation officers, children have an important job to do. Their task is to increase the amount of walking children and teachers are doing during the school day by working out an interesting walkway in and around the school and then promoting this walkway to get everyone moving.

1. In groups of three or four, children develop an interesting walkway in and around the school grounds. Mark the walkway out on a map of the school and then walk the pathway noticing any safety features that need highlighting, e.g. collision corners, obstacles (including plants and outdoor furniture), and other hazards such as open windows, doors, drains, driveways, etc. Mark these on the map.
2. Children estimate the distance of their chosen pathway and then walk the pathway again using a measuring device, e.g. tape, specific length of string, measuring wheel, etc. Record total distance of the walkway on the map.
3. Children promote their walkway to others in a creative way, highlighting features that would encourage others to walk their path.
4. Each group displays their created walkway on the wall for others to view. Include distance, safety warnings and distinguishing features. Include a space on the display for people to initial each time they complete walking the walkway during the school day. At the end of each week, children can tally up the distance they have walked around school and post it on their website profile.

3. THE GREAT WALK

As a class, ask the students to decide on a place in New Zealand where the children would (metaphorically) like to walk to, e.g. Eketahuna. Using Google maps find out the fastest route and the number of kilometres it is from school to the destination.

At the end of each week, tally up the distances that each individual has walked from the previous activity or any other walking activity and plot the equivalent distance out on a map. How close to the destination can the class get over the next seven weeks?

4. BUSTING BARRIERS

Briefly brainstorm and discuss barriers to walking as defined under the following categories:

- Physical environment, e.g. partial or non-existent footpaths, heavy traffic, non-existent or inappropriate crossings, poor quality walking surfaces, unpleasant surroundings (graffiti, lack of trees, etc.), geographic restrictions.
- Personal barriers e.g. lack of time, boredom, perceived safety issues, lack of fitness, disability, confidence, unwellness, and culture.
- Technology and how it has replaced walking, e.g. cars, lifts, escalators, remote controls, etc.

Each group receives a character card. (Character cards are provided in the resource box.)
Ask groups to read their card and then to imagine the character. What might they look like? Sound like? How old are they?

- Using the worksheet provided, identify and list all the barriers to walking the character describes.
- Give suggestions to how these issues may be solved and by whom?
- Groups' act/read out the character card to the rest of the class. Class members try to guess the character, e.g. elderly person. From the description, can the class identify two barriers to walking that this person faces?
- The group can then share two barriers and two solutions they thought of and ask the class for help with a barrier they identified but found difficult to solve.

Some examples with model answers are provided in the table below:

Type	Barrier	Solution(s)
Personal	Not enough time	Develop a weekly routine that includes time for walking.
	Can't be bothered	Schedule regular walks with a friend for motivation.<br>Walk to or from school every day so it becomes a routine.
	Too boring	Walk and talk with friends or family.<br>Walk a dog (or run it at a park).
	Too tiring	Start slowly by doing short walks, and then gradually increase as your fitness improves.
	No one to walk with	Trying asking your parents to walk with you to help you be healthier.<br>Look for other people at your school who live near you and walk to or from school with them (even if it's just part of the way).<br>Join a Walking School Bus.
Environmental	Dangerous people around	Ask your parents to walk with you or to take you to an area that is safer.
	Too many cars	Walk to an area with less traffic; try walking in a park (if it's safe) or at your school.
	No footpaths	Ask your parents to take you to an area where you can walk around.
	Unpleasant surroundings	Walk or ask your parents to take you to an area that is nicer to walk in. Perhaps a park or around your school.
Technological	Lifts and escalators	Try and beat the lift or escalator by walking up the stairs.
	Remote controls	Put them away and get up to change the TV channel.

SESSION 3: FOODS WE EAT

CURRICULUM LINKS

Achievement Objective	Intended Learning Outcome<br>We are learning to:	Success Criteria<br>We will know we have been successful when we can:
3D1<br>Societal Attitudes and Beliefs	Identify factors that influence what people eat across the world.	Identify similarities and differences in foods eaten throughout the world.<br><br>Identify factors that influence what foods are chosen, liked, and eaten by individuals.
3A1<br>Personal Growth and Physical Development	Understand the different ways healthy food keeps us well.	Describe the functions of different nutrients for our body.<br><br>List foods under the different food groups.

RESOURCES

Delicious Diversity: Meal pictures
Worksheet

Top of the Pops: Interview sheet
Fab Foods: Nutrient food cards
Herb Gardens: Seeds, containers, soil, water
Instruction sheet for planting

NUTRITION

Please note:
Talking about food can be a sensitive topic for some children.
Consideration and sensitivity for diverse home environments, body shapes, and feelings of self-worth will be required to support all class members.

- This resource is not about dieting and eating disorders. The aim of this teaching/learning unit is for children to develop awareness and understanding so they are able to make food choices for a healthy lifestyle, and to share this knowledge with their friends and families.

LEARNING NOTES

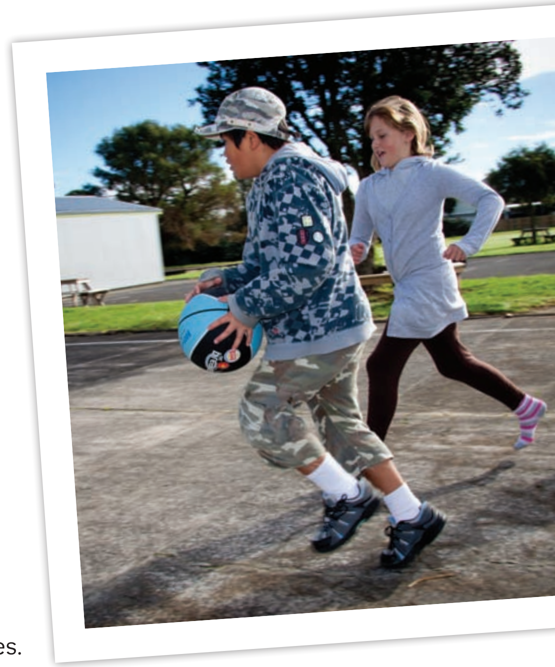

- Food is our source of sustenance, health, energy, and enjoyment.
- People need to eat a variety of different food to stay healthy and grow.
- The more active children are, the more energy they need from food.
- Food and eating are intertwined with and influenced by many aspects of our lives.
- Different diets may comprise of similar foods prepared or combined in different ways.

Physical benefits:
- Provides energy and nutrients.
- Helps maintain a healthy weight.
- Boosts the immune system.
- Builds strong healthy bones.
- Enhances the ability to concentrate.
- Provides warmth.

Social benefits:
- Learning about beliefs, traditions, and values of food within one's own and other cultures.
- How to join with others to prepare, serve, and share a meal.
- Understanding the significance of food in welcoming, farewelling, celebrating, and as a medium to draw people together.
- Every culture's interactions with food tell young people that food is more than just a fuel.
- Mental/emotional benefits.
- Can provide comfort, satisfaction, and pleasure.

CLASSROOM ACTIVITIES

Why do we eat different foods?

1. DELICIOUS DIVERSITY

Collect pictures of meals from different countries. Can children identify the country of origin? Place on a map of the world. What is the same and what is different about these meals? What things influence what people eat and how they prepare food? Are children able to name one traditional meal from their own culture? Can they name some of the ingredients of this meal?

2. TOP OF THE POPS

Have children brainstorm all the occasions they can think of where food is served and shared. (Birthdays, weddings, funerals, welcomes, farewells, special family occasions, playing at a friend's house, etc.)

Using the worksheet provided, children interview 10 people in the class to find out their most popular and unpopular food and the reasons for liking some foods but not others?

Foods I Like	Why	Foods I Dislike	Why

- What are the most common reasons given for liking or not liking certain foods?
- Culture, environment, and personal preference influence what people eat. What other factors can they think of that may influence food choices? (E.g. time of the day, climate, occasion, lifestyle, religion, money, etc.)

3. FAB FOODS

Find out what children know about food groups. Why do they think foods are grouped this way (share similar nutritional properties)? The food plate can be used to show how foods are grouped, and the recommended number of serves from each food group.

FOOD PLATE

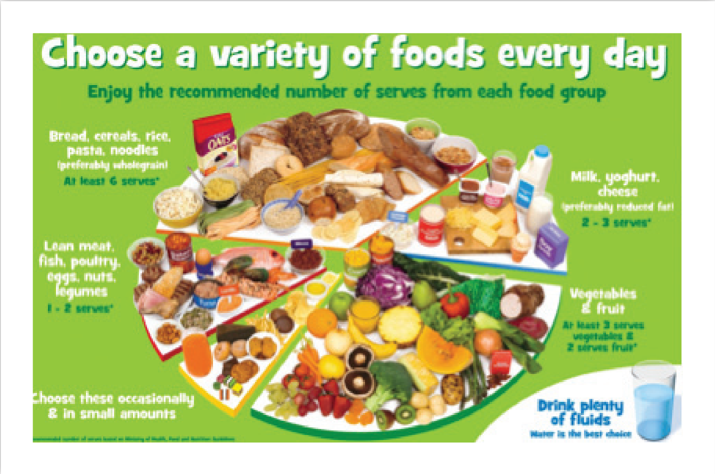

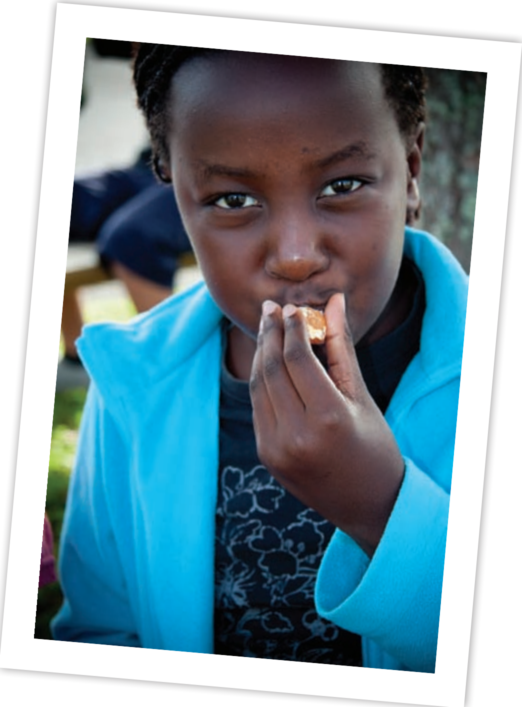

Food contains different substances (nutrients, water, and fibre) that are needed for health.

Discuss the following nutrients (list on board):

- Protein is needed for growth and repair (examples: beef, fish).
- Carbohydrate provides the main source of energy for the body (examples: potato, pasta).
- Fat is needed for health, but in small amounts (examples: oil, butter).
- Vitamin A is needed for night vision (examples: liver, pumpkin).
- Vitamin C is needed for the maintenance of healthy skin (examples: kiwifruit, blackcurrants).
- Iron is a mineral that is needed for healthy blood (examples: beans, beef).
- Calcium is a mineral that is needed for the growth and maintenance of strong bones and teeth (examples: milk, cheese).
- Fibre is needed to help keep the gut healthy. It speeds up movement of waste through the colon (examples: wholegrain bread, grain-based cereals, fruit and vegetables).

Ensure that children understand measurement, e.g. gram, milligram, and kilojoule.

Group children and provide each group with a set of food cards that list the nutrient values of different kinds of foods.

- Which foods are rich in fat? Calcium? Iron? Vitamin C?
- Do all the foods that are high in each of these nutrients belong to the same food group?
- What else do they notice?

4. HERB GARDENS

Plant herb seeds with the children in either the school garden or in a pot in the classroom. Seeds have been provided in the Healthy Homework resource box.

With water and care, it should grow big enough to use in the children's meals in the final week of Healthy Homework.

Are herbs considered to be food? Ask children what food group they would put herbs under and why?

WEEK TWO

SCREEN TIME / ENERGY FOODS

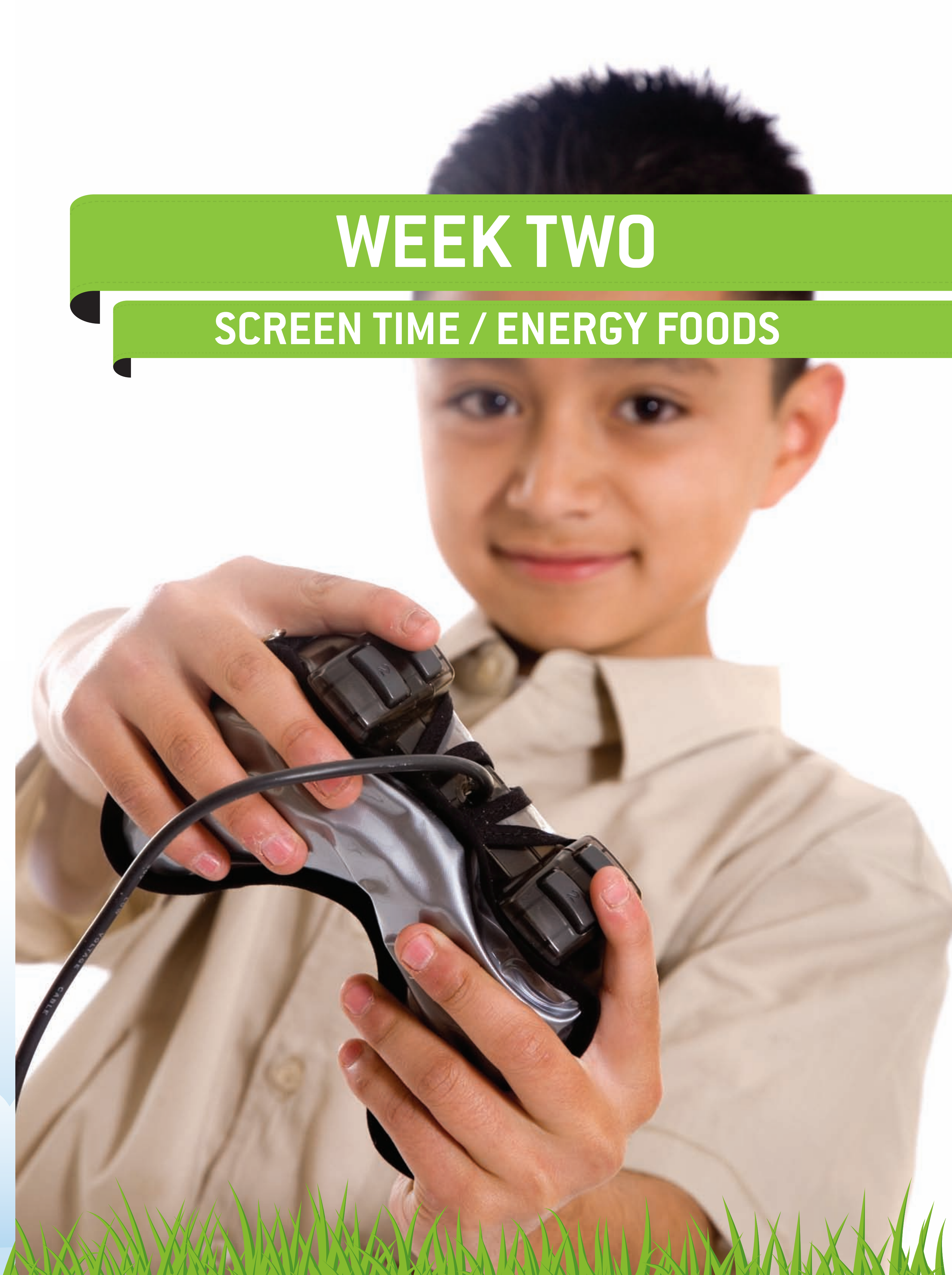

WEEK TWO

SESSION 1: HOMEWORK REVIEW

WALKING

Go through all three homework options:

Option A: Discuss with class whether they were able to complete two family walks. If they were not able to, discuss what the barriers to walking with the family were and how these could be overcome. If the family were able to walk together, what types of things did they see and do while walking that they may not have experienced if they were inside watching television? Encourage children to continue with this exercise, emphasising the benefits to the whole family and relationships.

Option B: In groups children share what they learned in their discussion with an older person. Ask them to compare the generations. Are there similarities and/or differences in how, where, when, or why people walked back then as compared to now? Do they think that attitudes towards walking were the same or different? Ask the children to give reasons for their answers.

Option C: Ask three children who were able to walk to school or part of the way to school to discuss with the class any difficulties they overcame to complete the task, whether they felt safe, and if they will continue walking to school when the Healthy Homework programme is finished. Were there any enjoyable things about walking?

OR What car trips were replaced by walking? What thoughts and feelings did children have before, during, and after doing this task? Discuss with the class the benefits of walking, e.g. physical, social, and mental/emotional, versus driving. Get the children to brainstorm trips that can easily be replaced by walking. Encourage them to continue replacing some of their trips with walking.

FOODS WE EAT

Go through all three homework options:

Option A: In groups looking at their recipes, children discuss what makes recipes different from each other. (E.g. ingredients, time for cooking, style of cooking, presentation, etc.)

Children place food recipes around a world map indicating which country it comes from.

Which area of the world are most dishes the class has gathered from? What are the similarities of the dishes from this area?

Option B: Compare the popularity of foods at home with popularity of foods in the class and the reasons for personal preferences.

Option C: Play bingo, call out different foods from each food group and if the student has that food listed they give it a tick. If children end up with ticks against all their listed foods, they call out bingo!

Ask each group to choose a recipe that one member brought to school. Ask them to place the ingredients of the recipe into the different food groups and name the nutrients they think each ingredient contains.

SESSION 2: SCREEN TIME

CURRICULUM LINKS

Achievement Objectives	Intended Learning Outcomes<br>We are learning to:	Success Criteria<br>We will know we have succeeded when we:
3D1<br>Societal Attitudes and Beliefs	Explore and describe how screen time can influence what we eat and how active we are.	We have created a timetable showing how much time we spend doing different activities and can draw conclusions.<br><br>We can describe some of the advantages and disadvantages of screen time.<br><br>We can plan and take action to help ourselves or others minimise screen time.<br><br>We can describe the aims of advertisements and what strategies they use to make products attractive to young people.
3A2<br>Regular Physical Activity	Set realistic and achievable physical activity goals to look after ourselves.	We have created and practiced fun physical activities to do with family members while we are watching television.

RESOURCES

Super Sleuth: Weekly timetable
Wooing You: Advertising DVD
Television Advertising Worksheet

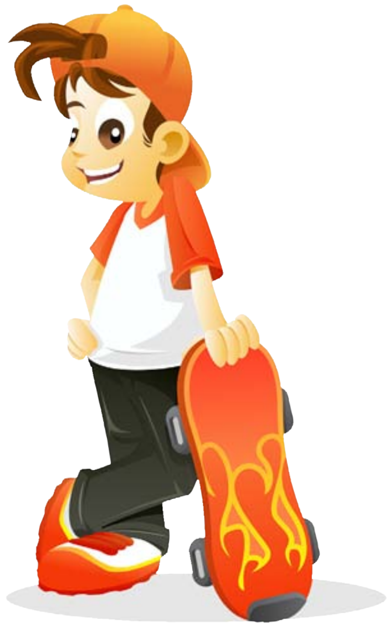

LEARNING NOTES

Xbox, PlayStation, computer games, and playing on cell phones all contribute to on screen hours.

Too much television or screen time may:

- Distract people from communicating.
- Gobble up potential physical activity time.
- Provide more opportunity for snacking.
- Promote unhealthy food options through advertising.
- Impact on children's behaviour and educational outcomes.

The media influences physical activity and healthy eating choices.

TEACHER NOTES

- NZ health agencies currently recommend that children should watch no more than two hours of television per day, preferably less.
- Not all television watching has a negative influence; it may be watched for educational and relaxation purposes.
- Learning focus is to encourage children to create or maintain a balance, watching television in moderation.

CLASSROOM ACTIVITIES

1. SUPER SLEUTH

Explain to the children that they are about to embark on a private investigation to help you with finding answers to an interesting trend that has come to your attention. Tell them that you have heard a fictitious rumour that some young people are at risk of developing the disease "square eyes".

1. Discuss with children their understanding of the words: trend, fictitious, and rumour.
2. Ask children to play along and wonder with you using the following questions:

   (Be the naïve inquirer who is interviewing the experts . Gently prompt children to be curious and to think creatively and critically in order to construct knowledge.)

   - What do they think this disease may be about?
   - How would someone know if they had caught this disease?
   - How might people catch this disease? (Too much time watching television, playing Xbox, PlayStation, and time spent on the computer, iPod, iPad, screen phone.)
   - What other symptoms might a sufferer of this disease have? (Think social, emotional, mental, and physical, e.g. unable to hear parents calling them, grumpy when told to stop watching 'a screen' or when the screen is turned off, not knowing what to do with themselves if they cannot watch 'a screen', sneaky behaviour to ensure screen time, unmotivated to play outside, etc.)
   - When might young people be most at risk of catching "square eyes" (after school, when they get a new game, when other people are busy in the house, when friends cannot play with them)?
   - What are the possible long-term effects on someone, if this disease cannot be cured? (Explore possible behaviour, physical, social, and education issues.)
   - What do they think may be the cure for this disease?

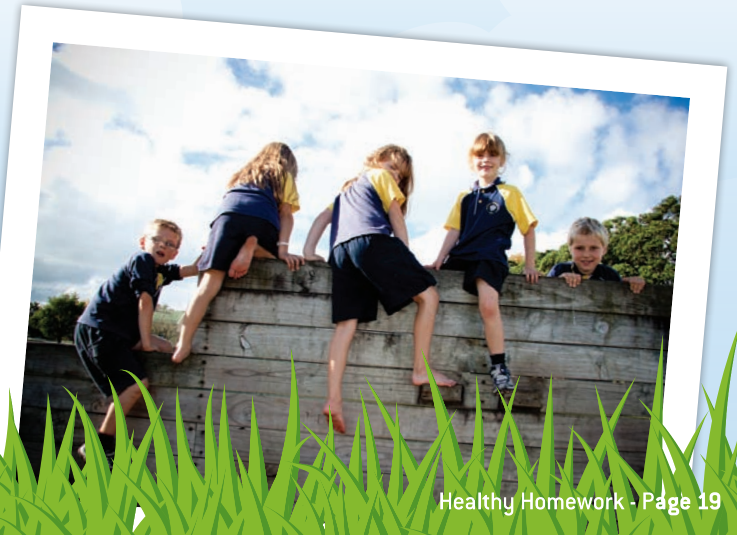

3. The job of this class of private investigators is to see if there is any truth in the rumour. The first priority is to ensure they are not at risk of catching the "square eye" disease themselves.
   - Ask children how they might go about this investigation to ascertain their own screen watching time. Utilise their ideas or follow the process below.
   - Ask them to make an estimate of how many hours a day and week they may spend watching 'a screen', sleeping, and participating in physical activity.
   - Provide children with a weekly timetable. Ask the children to reflect on the last week and fill in their timetable naming the activities they participated in. Use different colours for each of the following:
     a. Television
     b. Other screen time (except when screens are not used for entertainment, e.g. homework, finding information, etc.)
     c. Sleep time
     d. Participation in physical activities
     e. All other activities that do not involve watching 'a screen', sleeping, or physical activity, e.g. eating, reading, talking with friends, etc.
   - At the bottom of each day add up the total for each activity. Calculate the weekly total for each colour. How close to their estimate did they get?

Please note:
Emphasise that this is an investigation to create awareness and that there is no right or wrong information to gather, but that for the investigation it is helpful to be as accurate as possible.

To avoid any potential for shaming, it is suggested that timetables are not shared or displayed but that the children anonymously write the total amount of hours spent on watching screens, sleeping, physical activity, and other activities for the week on a piece of paper to be collated as part of the class investigation.

4. Write totals on the board under each heading or have a couple of children collate and add numbers together so there is a class total. Work out the percentages and have the children create a pie graph of how people in Room xxxx spend their time. What conclusions can they draw?

5. Ask children to think about the following statements, and give reasons for why they think the statements are true or false.
   - Television is 2nd to sleep as the activity most engaged in.
   - The average child spends one whole day per week in front of the screen.

   Look at the collated class television viewing hours. Is there any truth to these statements?

6. With your naïve inquirer hat on, lead a discussion using the following suggested questions:
   - How much do they think is too much television? Have them give their reasons.
   - Why do they think young people are so drawn to screen activities?
   - What might make it difficult at times to stop the screen activity or to choose to do something different?
   - What else do people do while they are watching the screen (interact with controls, snack, fidget, keep silent, nothing)? What do they think of these activities? Do they think there might be any long-term effects of any of these activities?
   - What are people not doing or doing very little of while they are watching 'a screen' (moving, talking, making eye contact, thinking).
   - What things might they do if they were not watching 'the screen'? Make a list on the board and have children jot down activities they may enjoy doing.
   - Were they surprised at how much time or how little time they spend in front of a screen?

Ask children to turn to a partner and think of suggestions for helping those who may benefit from minimising their screen time, e.g. schedule time, plan screen time and activities for non-screen time, involve parents to support, find healthy ways to reward self for efforts, create a plan for when you are tempted to watch television, e.g. distraction skills, ring a friend, listen to music, etc.

- Now that they have investigated their own and the class' screen time, wonder with them what would be if the fictitious rumour of the disease "square eyes" for young people was true. Have them identify ways in which screen time may impact on participation in physical activity

2. WOOING YOU – TELEVISION ADVERTISING

TEACHERS NOTES

- Although there are laws restricting the types of food and drink that can be advertised during children's viewing times, the food industry continues to spend billions of dollars each year devising innovative marketing strategies to make their products more appealing to children.
- Junk food advertising targets children in three ways. By aiming to:
  - Instil brand loyalty.
  - Create the 'Pester Factor'; hoping children will influence their parents to buy the product.
  - Create the message that children's food is fundamentally different to adult's food (i.e. more colourful, fun, etc.).
- It is hoped that by increasing children's awareness of the aims and strategies of advertising, individuals will become increasingly more informed and skilled in making health-enhancing choices regarding food choices throughout life.

1. Ask children the following:
   - How do you feel towards advertisements in general? E.g. do they get annoyed that the programme is interrupted, do they look forward to what products are being advertised, etc.?
   - What do you do when advertisements come on television? E.g. turn the television down, watch the advertisements, do something else (bathroom, eat, talk, leave the room).
   - What do you think makes a good or not so good advertisement?
   - What do you think is the role of television advertising? E.g. to inform, to entertain, to persuade, to raise the profile of celebrities, to promote a product, to compete against similar products, to develop brand loyalty, etc.
2. Ask the children to share with a partner:
   - A favourite advertisement and why it is a favourite.
   - Any advertisements that after they have watched them, they attempt to persuade their parents/caregivers to watch, in hope that they will buy the product being advertised?
3. Can they think of ways advertising is made interesting for children? E.g. bright colours, smiling faces, funny characters, slogans, music, jingles, cartoons, etc.
4. "All is not as it seems". What do the children think this statement may mean. Could we and how might we apply this saying to advertising on television?

The DVD provided in the resource pack has examples of television advertisements.

5. Show the DVD and have children identify the intended audience of each advertisement and any strategies that have been used to make the advertisements more appealing to this target audience.
6. Hand out the "Advertising on Television" worksheet that asks children to answer key questions on each of the five lessons on the DVD. Give the children time to answer the questions for a particular lesson and discuss their answers in class before you move on to the next lesson. Some general questions you could ask during class discussion that could apply to most of the advertisements shown include:
   - How would you rate those advertisements and why? (Boring, funny, sad, average, good, great.)
   - How do you expect to feel if you bought the product?
   - Advertisements often tell us just the good things about a product. What is intentionally not mentioned and why?
   - What did the advertisers do to make the product look so appealing?

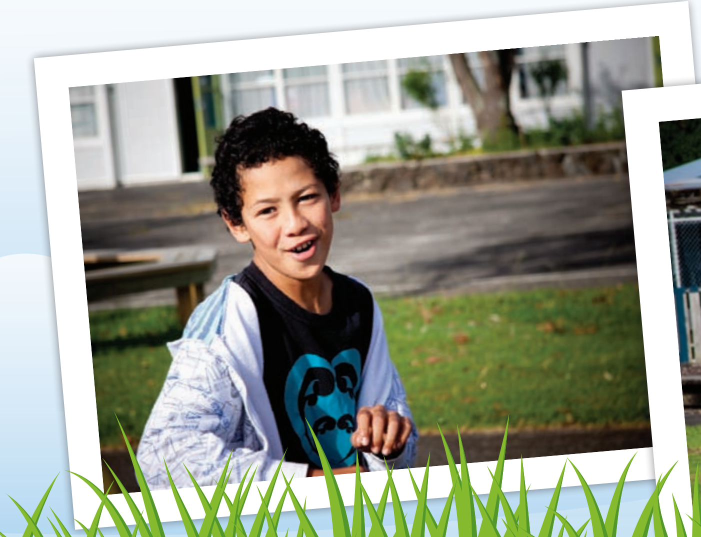
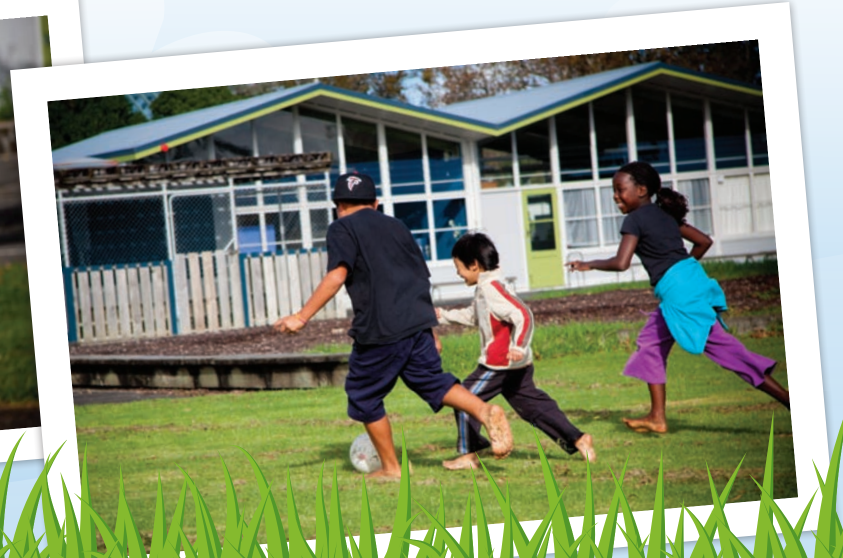

SESSION 3: ENERGY FOODS

CURRICULUM LINKS

Achievement Objectives	Intended Learning Outcomes<br>We are learning to:	Success Criteria<br>We will know we have succeeded when we:
3A1<br>Regular Physical Activity	Understand that the food we eat provides us with the energy we need to take part in physical activity.	Describe the relationship between food and our ability to participate in different activities.<br><br>Work out nutritional needs for different physical activities.

RESOURCES

Rocket Fuel: Food energy cards
Outputs: Activity energy cards

LEARNING NOTES

- Energy comes from a range of sources, e.g. sun, petrol, food, fire, etc.
- Food enters the body and is used by the body in different ways. One way the body uses food is for energy.
- Food is the body's source of energy.
- We need energy to grow, maintain health, and be active.
- Different types of food provide different amounts of energy.
- Different types of activities use up different amounts of energy.
- What you eat and drink is ENERGY IN. What you burn through your metabolism, food processing, and physical activity is ENERGY OUT.
- Energy used through physical activity can be broken down into exercise and non-exercise physical activity. For most people, non-exercise activity uses up more energy than exercise activity.

ENERGY WE USE

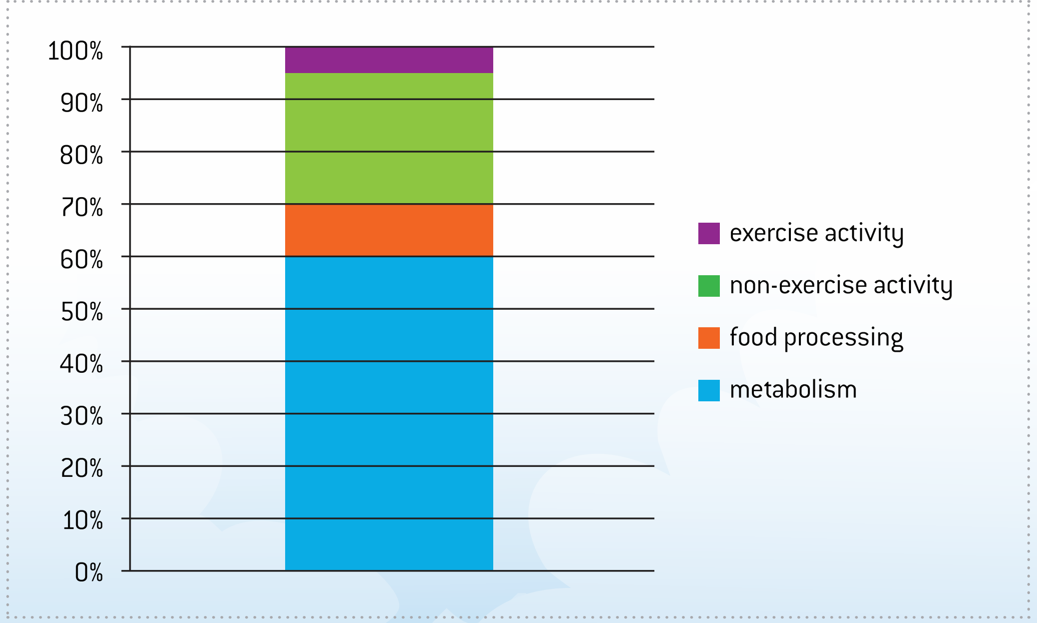

- Becoming aware of when we may need to increase or decrease our food intake ensures we have enough energy to partake in the activities of the day.
- Energy in food is measured in kilojoules (kj) or calories (Cal).
- 1 Cal = 4.18 kj

CLASSROOM ACTIVITIES

1. ROCKET FUEL

Using the list or the cards provided, place these different foods on a continuum with those containing the most energy at one end of the continuum and those providing the least amount of energy at the other end.

Per serving of these foods:			
Bread (1 slice)	224kj	Apples (1 medium)	315kj
Milk (1 glass)	363kj	Kumara (1 medium)	471kj
Margarine (1 tsp.)	152kj	Cheese (2 slices)	531kj
Tomatoes (1 medium)	86kj	Kiwifruit (1 medium)	190kj
Rice (1 cup)	487kj	Eggs (1 medium)	64kj
Steak (1 medium)	1316kj	Banana (1 medium)	552kj

Discuss the differences between these amounts. Did these results surprise you? Why?

2. OUTPUTS

Different activities use different amounts of energy.

The cards provided give approximate energy usage for each activity.

> Please note: the energy used is an approximate for children of this age, but does not take into consideration a particular child's level of intensity while engaged in the physical activity or the weight/size of different children. The focus here is on creating an awareness of ensuring we have the right balance of energy in and energy out for optimal health and weight control.

Children are to place these activities on a continuum with those using up the most energy at one end of the continuum and those using up the least amount of energy at the other end of the continuum.

Kilojoules (energy) burned per hour of exercise	
Cycling	586 kj
Running	1172 kj
Football	1025 kj
Swimming	879 kj
Walking at a moderate pace	586 kj

Ask them to talk to a partner about why some of these activities use up a lot more energy than others. Did this surprise them? Why?

Engage children in a discussion on ways that we can increase our non-exercise activity energy expenditure, e.g. standing instead of sitting, walking and talking on the phone, helping around the house etc.

3. FILLING THE TANK

With their partner use the cards from both Activity 1 and Activity 2. Ask them to try to work out an interesting combination of food they could eat in order to have enough energy to:

- Play football this week in an hour-long match?
- Walk to school? Your walk will take you 15 minutes.
- Participate in a swimming lesson that lasts for 30 minutes?
- Riding your bike for three quarters of an hour?

What do they notice about the amount of food they need to eat? Is it more or less quantity of food that they originally thought was needed?

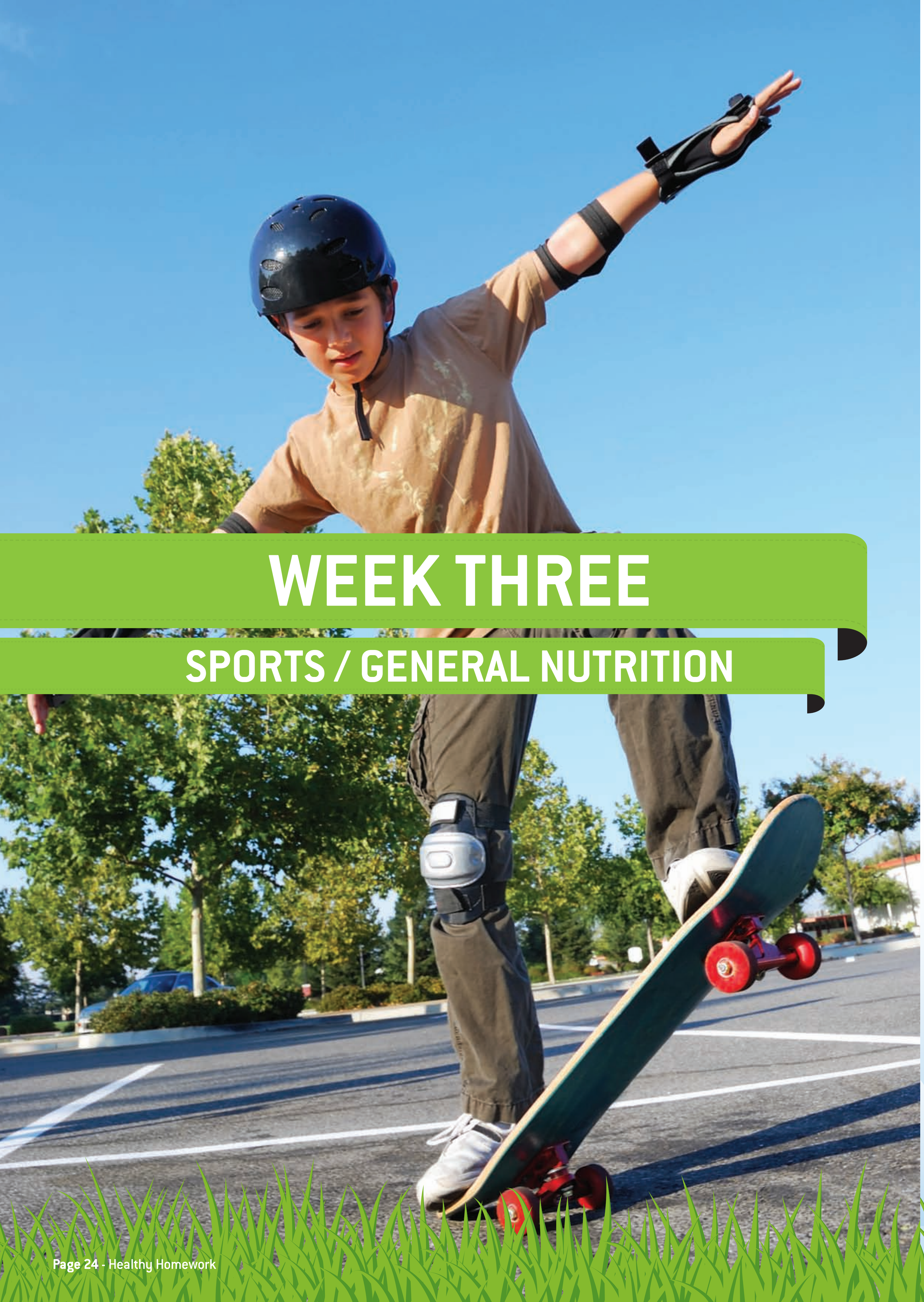

WEEK THREE

SPORTS / GENERAL NUTRITION

WEEK THREE

SESSION 1: HOMEWORK REVIEW

SCREEN TIME

Go through all three homework options:

Option A: Discuss with the class the activities they did as an alternative to watching television this week. Did they miss watching television? Could they continue to watch less television on a daily basis?

Option B: In small groups let the children share something about their “ad action” experience (could be what it was like to get up and move or maybe some funny action movements that they came up with). What were some of the other physical activity ideas that they thought of with their family to do during the advertisements?

Option C: Collate data and discuss the outcomes of tally charts and draw conclusions. How do children think that advertisements may be different during the adult viewing hours? Although many children do not have money to spend on the products being advertised, advertisements are still shown during children’s programmes. What reasons can children come up with for why this happens?

ENERGY FOODS

Go through all three homework options:

Option A: In groups, ask children to discuss how they felt during the three different activities that they completed. How did their perceived exertion relate to the amount of kilojoules that they were using?

Option B: In the same groups children can discuss the differences in the kilojoules per serving of the healthy and unhealthy foods that they selected. How much walking/running do they need to do to burn off each of the foods? How might their results affect the foods that they choose to eat when they are very active or when they are not doing much activity?

Option C: Children can present their posters to the class. Why do they think non-exercise activity is important? Did anyone in the class try to increase their non-exercise activity during the week? Remind the children that it is important to find ways to increase our non-exercise activity every day.

SESSION 2: SPORTS

CURRICULUM LINKS

Achievement Objective	Learning Outcome<br>We are learning to:	Success Criteria<br>We will know we have succeeded when we:
B4<br>Social and Cultural factors	Play an unfamiliar sport and describe how competition can affect how people play.	Have learned about and played ki o rahi.<br><br>Have participated in a continuum of activities to explore our points of view about cooperation and competition.
D1<br>Societal Attitudes and Beliefs	Identify how sport is influenced by culture and the environment.<br><br>Find out and locate different sporting clubs in our local area.	Have researched and recorded three facts about how a sport developed in another country.<br><br>Describe the importance of sports clubs in supporting people’s participation in sport.

RESOURCES:

Ki o Rahi: Ki o rahi DVD
www.kiorahi.co.nz for legend
Ki o rahi instructions

Culture Counts: “Make the links” worksheet

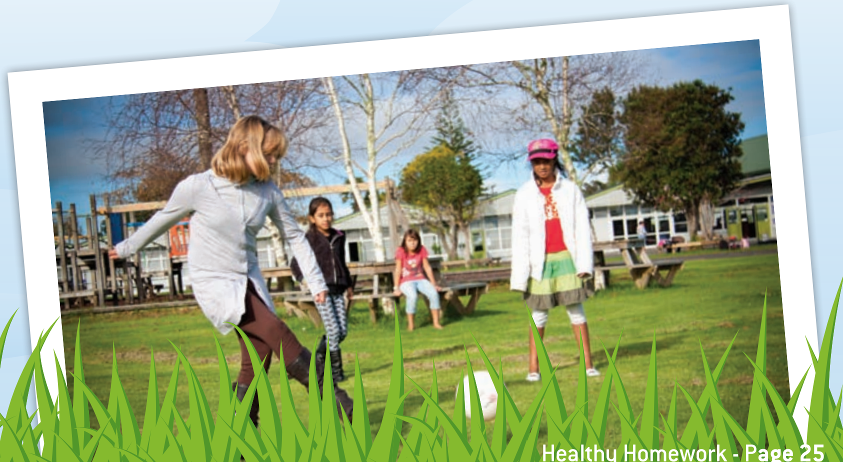

TEACHER NOTES

- Sport has the potential to contribute both positively and negatively to wellbeing.
- The effective promotion of sport ensures all children have the opportunity to:
  - Participate to the highest level of their interest and ability.
  - Experience enjoyment and achievement.
  - Become competent and enthusiastic participants.
  - Practice fair play in all situations.
  - Experience and manage competition.

(1999, Ministry of Education. p.44)

It is also important to recognise that not all children wish to or are able to engage in competitive sports. It is hoped that schools also provide non-competitive options to support these children to engage in physical activity.

Competition: Team sports provide opportunities for children to develop teamwork and cooperation skills, manage success and disappointment, and to respect officials, teammates, and the opposition.

Movement skills: It is hoped that parents and teachers encourage children to experience a range of sporting activities over their developmental years. The larger a child's fundamental movement skill set, the easier they will find it to involve themselves in sport throughout their development and into adulthood.

Leadership: Opportunities to acquire skills of coaching, officiating, and administrating are thought to support a child's understanding and knowledge of game play and help to develop leadership skills.

Culture: Children who experience sporting activities, in which cultural practices are expressed through movement, develop skills to identify and discuss the social and cultural significance that sport has for individuals and for society.

CLASSROOM ACTIVITIES

1. KI O RAHI

Ki o rahi is a fun, fast paced, traditional Maori ball game. The sport incorporates skills similar to those in other ball games such as rugby, netball, and touch.

- Introduce children to the legend that led to the development of the Maori sport ki o rahi. Discuss.
- Either explain and play ki o rahi, or challenge the children to work out how it is played and set the game up for themselves.
- Once they are familiar with the rules, encourage them to have a ki o rahi competition.
- Children reflect on their experience of playing ki o rahi. What did they enjoy? Is there anything they would like to change about the game that might make it more enjoyable? What is meant by sportsmanship? What is the meaning of "teamwork"? In what ways might participation in sport be good for you, the community, and the nation?
- Suggest that the children may like to continue playing ki o rahi at lunchtime.

2. CULTURE COUNTS

- Ki o rahi originated in Aotearoa/New Zealand. Reflect on the factors that influenced the development of ki o rahi.
- Ask children to make a list of sports they know and write the country of origin beside it if it is known.
- Using the worksheet provided, children match the sports with the country they think it may have originated in. Below is a list of the answers.

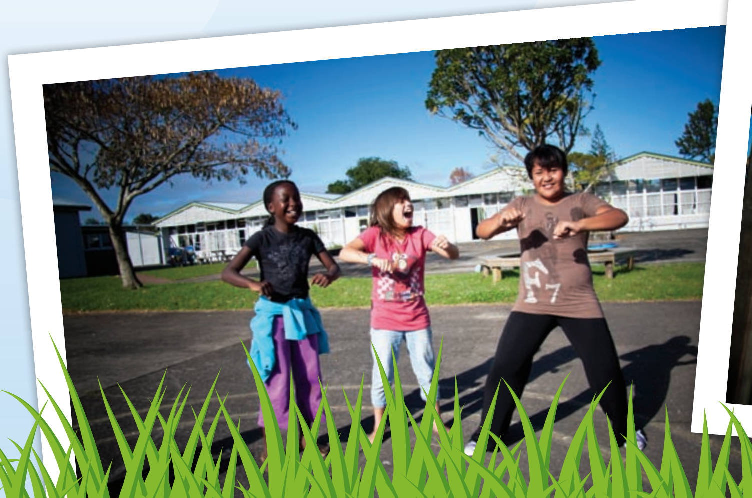

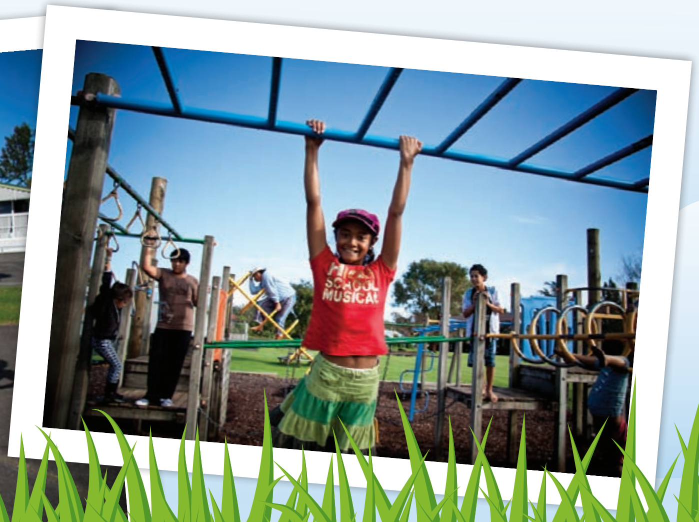

Sport	Country
Sumo Wrestling	Japan
Dragon Boat Racing	China
Kilikiti	Samoa
Australian Rules	Australia
Baseball	United States of America
Ice Hockey	Canada
Lacrosse	North America
Netball	England
Hurling	Ireland

Which of these sports do children think may have originated to tell a traditional cultural story?

3. COMPETITION CONTINUUM

Set up a continuum with disagree at one end and agree at the other.

Reading out the following questions, ask the children to stand on the continuum where they feel their answer lies. Then act as a reporter and ask children from each viewpoint to explain their opinion. See if anyone wishes to change their mind and their position as they hear different perspectives.

Teacher note: There is no right or wrong place to stand. This is a chance for them to explore their thoughts and to listen to those of others.

- Winning is the most important thing when you play sport.
- Fair play is boring.
- Close scoring games are more fun to play.
- Competitive sports are not as enjoyable as just playing for fun.
- To be competitive you have to be aggressive.
- Team stands for "Together Everyone Achieves More".

SESSION 3: GENERAL NUTRITION

CURRICULUM LINKS

Achievement Objective	Intended Outcomes<br>We are learning to:	Success Criteria<br>We will know we have been successful when we:
3A1<br>Personal Growth and Development	Categorise foods so that we can make healthy balanced food choices.<br><br>Understand how healthy eating contributes to our wellbeing.	Can use the traffic light system to list different foods.<br><br>Know how the foods we eat make us feel.

RESOURCES:

Ready Steady Go: Pictures of a variety of foods (3 per child)
Food diary
Fed Up: Shop for Your Life DVD

TEACHER NOTES

The Traffic Light Food Labelling system categorises foods within the red, amber, or green colours. These colours correspond to 'eat only occasionally', 'eat in moderation', and 'eat freely', based on the nutritional value, energy density, and levels of saturated fat, added sugar, salt, and fibre of foods.

RED FOODS AND DRINKS

Foods and drinks classified as Red are energy dense and nutrient poor that are typically high in saturated fat, sugar, and/or salt. They can contribute to excess energy intake if consumed in large amounts or on a frequent basis.

Red foods also include deep fried foods, confectionary, and chocolate (energy size limit), crisps, corn chips, and similar salty snacks (energy size limit), sugar sweetened soft drinks, as well as energy and sports drinks (energy size limit).

These foods and fluids should only be eaten occasionally.

Examples include: Fried foods, savoury commercial products such as pies and sausage rolls, snack bars, sweet biscuits, cakes and sweet pastries, small size confectionary and packets of crisps, some sweetened drinks and processed meats such as salamis.

AMBER FOODS AND DRINKS

Foods and drinks classified as Amber are mainly processed foods. They have some nutritional value but may contain moderate levels of fat (particularly saturated fat), added sugar, and/or salt and can, in large serve sizes, contribute to excess energy intake.

These foods and fluids should be carefully selected and eaten in moderation.

Examples include: Full fat milk and dairy products, some breakfast and cereal bars, some un-iced, plain, lower fat cakes and muffins, some processed meats (e.g. ham, pastrami), poly- or mono-unsaturated spreads, breakfast cereals with no added sugar or fat.

GREEN FOODS AND DRINKS

Foods and drinks classified as Green are the healthiest choices. They are excellent sources of important nutrients needed for health and wellbeing, and low in saturated fat, added sugar, and salt.

These foods/fluids should be eaten often, i.e. every day or at every meal.

Examples include: Plain or whole grain breads and cereals, vegetables and salads, fruit, low fat milks and dairy products, lean meats, fish and poultry, eggs, and nuts and legumes.

CLASSROOM ACTIVITIES

1. READY STEADY GO

- What are traffic lights used for? What does each of the colours represent?
- What do the children think might happen if we didn't have traffic lights?
- Have the children discuss food in relation to the traffic light system outlined above. (Categorising food and emphasising the benefits of having a balanced food intake. Introduce the concepts of occasional, moderate, and frequent consumption in relation to different foods.)
- Randomly distribute three food pictures cut from magazines to each student.
- Children tack their pictures to the appropriate traffic light colour that are placed on the walls around the room.
- Children walk around and see if they agree with all the placements and discuss any changes they would like to make.
- Display traffic light in order in a prominent place in the classroom.

2. FED UP

This activity relates to Homework Option B

Ask children to think about everything that they ate and drank in the last day (i.e. 24-hours) and write it down in detail on the food diaries provided. They should write down the amount of the item that they ate and if possible the brand.

An example for breakfast:

- 1 bowl cornflakes (Kellogg's), tsp. sugar, half a cup of milk (Trim)
- 2 pieces white toast, butter, and jam

Divide the class into groups of two or three children and ask them to design their own traffic light poster categorising the foods from their diaries into red, amber and green categories.

Ask groups to report back to the class on the foods they have categorised. Engage in discussion about why each food fits within each category.

TEACHER NOTES

Children can keep their food diary to help with homework option B.

Play the "Shop for Your Life" DVD to the class. This DVD is a fun, educational approach to healthy shopping and food choices. Afterwards engage the children in a class discussion about what they learned and what they already knew from the DVD.

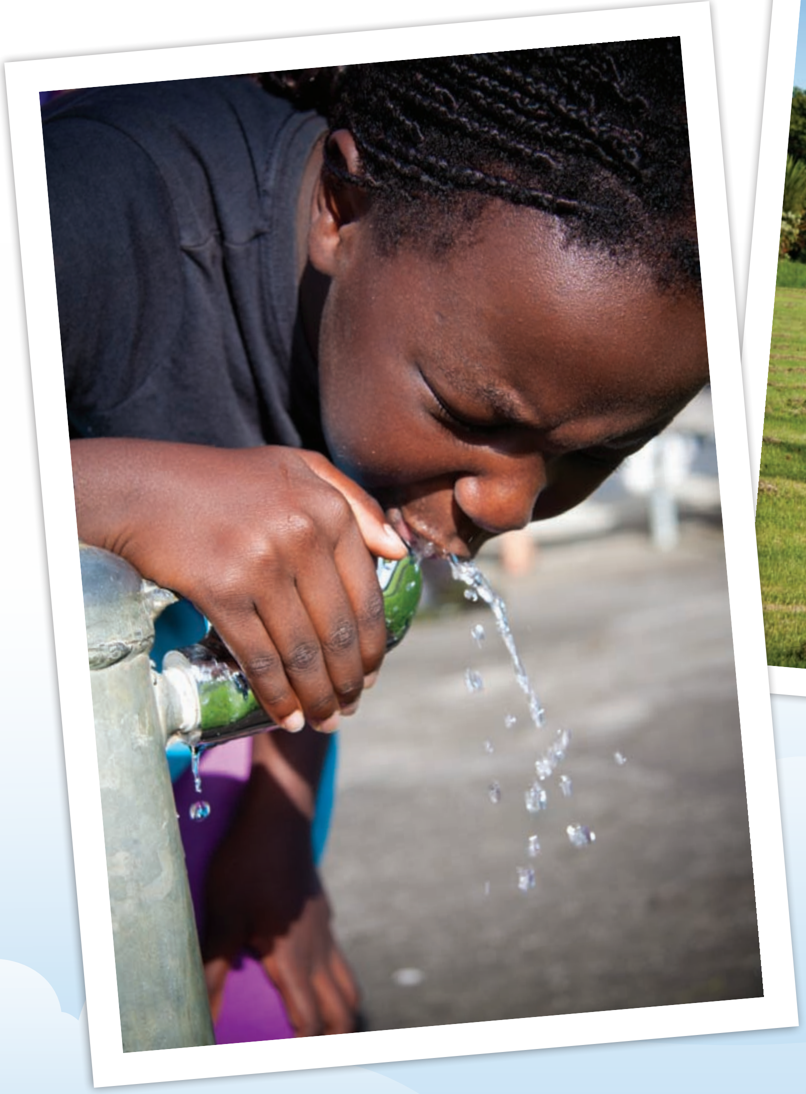

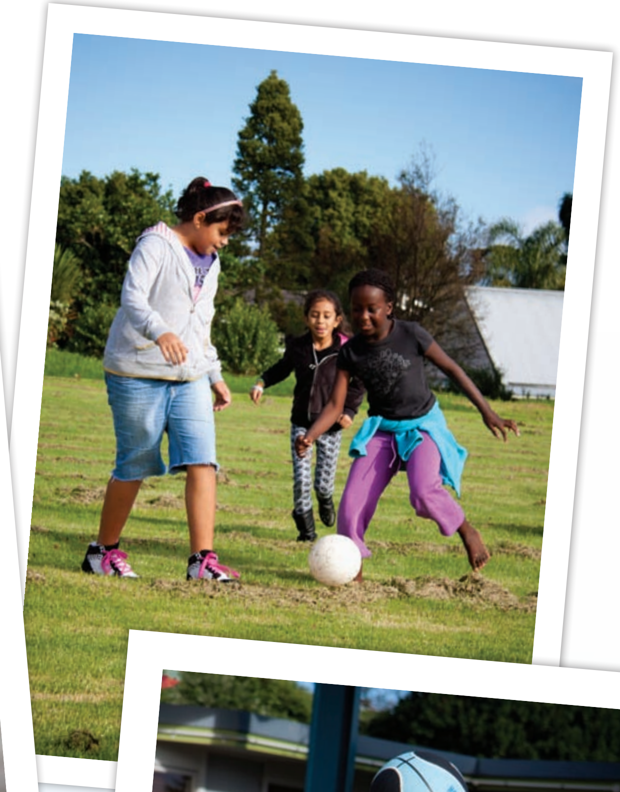

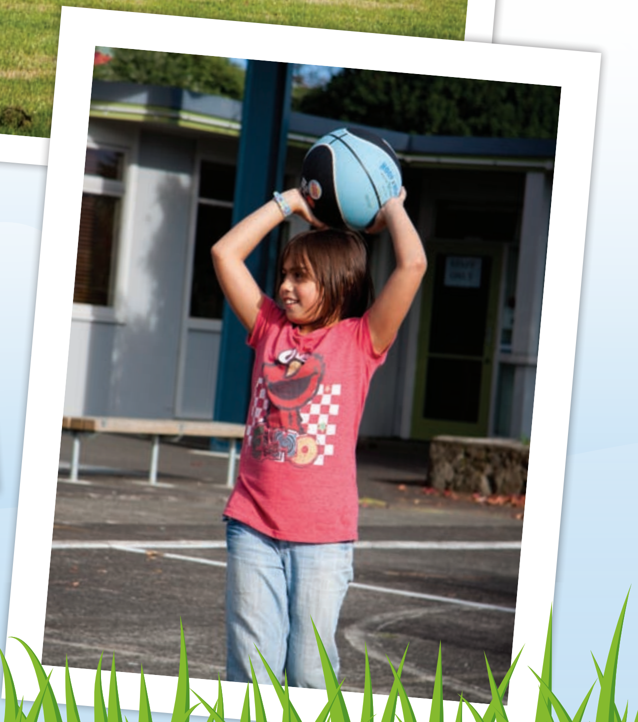

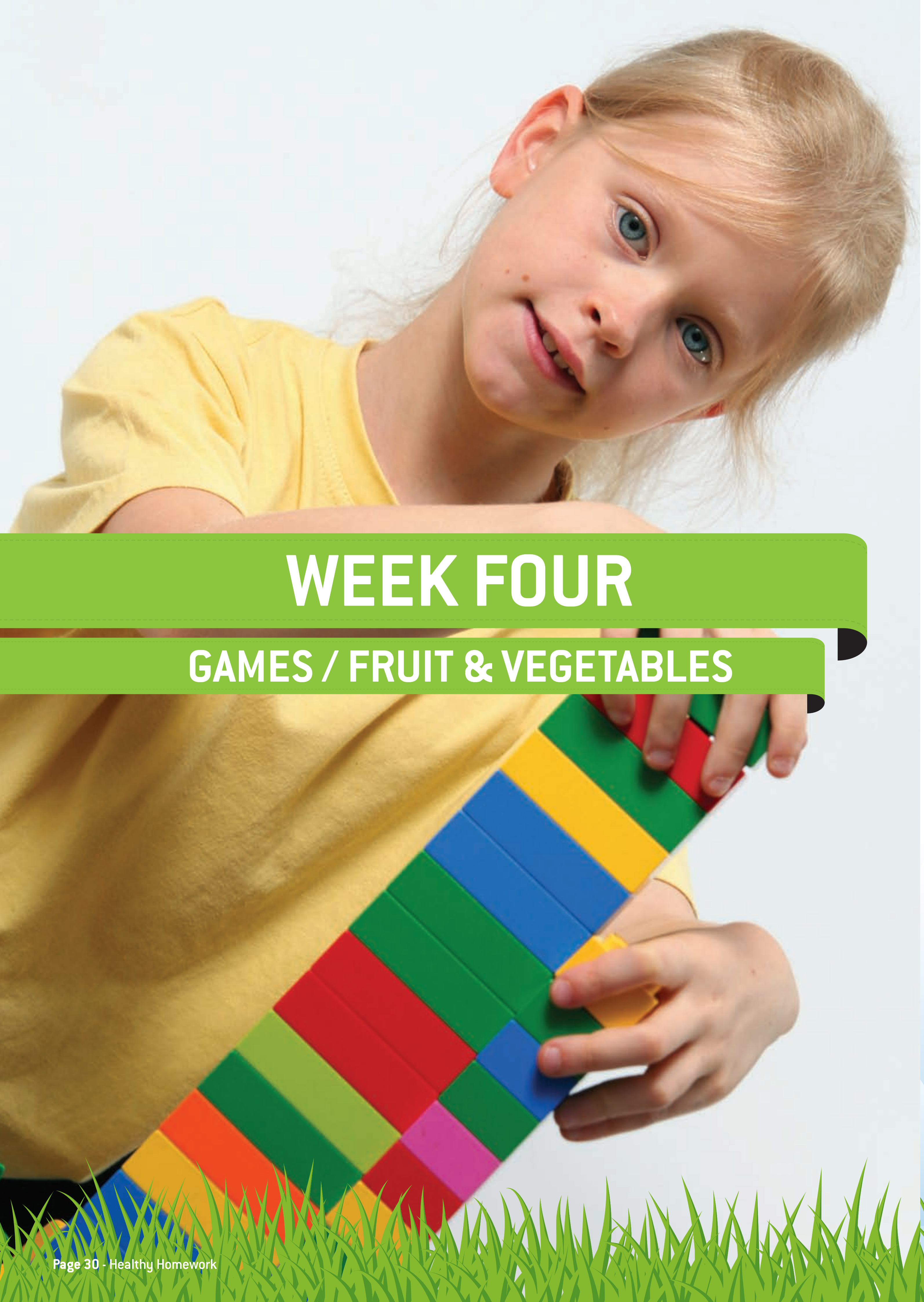

WEEK FOUR

GAMES / FRUIT & VEGETABLES

WEEK FOUR

SESSION 1: HOMEWORK REVIEW

SPORTS

Go through all three homework options:

Option A: Discuss with the class how it felt to practice skills for ki o rahi or your chosen sport? Was it difficult? Ask them to imagine coaching someone else in these skills, what would be the important things to emphasise? Ask three children to coach the class in the skill they chose.

Option B: In groups of 3-4, get the children to report back to each other on the different clubs they went to. Did anyone in the group find out some information on joining a club or do they already belong to a club? What are the advantages of belonging to a sports club?

OR Using a world map, ask children to place the name of the sport they researched on its country of origin. In groups children share what they learned about their sport and the culture it came from. Do they know if this game is played widely in New Zealand? Why might it be or why not?

Option C: Discuss the different types of sports observed. What differences did they see in how adults on the sports field/court interact with each other to how children interact while playing sport? Do the children aim to continue playing sport as adults, why or why not?

GENERAL NUTRITION

Go through all three homework options:

Option A: Ask children to give an example of one item they found at home that they listed under each traffic light colour.

The following games aim to reinforce children's understanding of categorising foods under the traffic light system.

TRAFFIC LIGHTS

Version 1: Area: marked court area. Children stand in the middle of the court. Indicate which direction over the court line, represents RED, AMBER and GREEN. Teacher calls out a food and children run fast in the direction of the category that the named food comes from. E.g. if teacher calls pumpkin, children race to cross the line in the GREEN direction.

Version 2: Area: marked court area. Teacher calls out a traffic light colour and the children respond accordingly, e.g. RED – Freeze, AMBER – walking, and GREEN – fast running.

- Replace colours with different foods. Ask children to freeze, walk or run according to which category they think the food belongs.
- Replace walking and running with other modes of movement.

Option B: What food group did they have the most listed in? Why do they think this is? What makes a balanced diet?

Option C: Can children name the links between what they like to eat and how it makes them feel or how it influences their wellbeing?

SESSION 2: GAMES

CURRICULUM LINKS

Achievement objective	Learning outcomes<br>We are learning to:	Success criteria<br>We will be successful when:
B2<br>Positive Attitudes and Challenge	Develop throwing and catching skills that give us confidence to make up and play games.	We can throw and catch a range of objects in a variety of ways.<br>We use our skills in a team game situation.
C1<br>Identity Sensitivity and Respect	Be aware of people's differences and make sure anyone can join in and enjoy playing our games.	Make up a game and teach it to other people.<br>We can change a game so everyone can participate and enjoy playing.
D1<br>Societal Attitudes and Beliefs	Identify how and why games change over time and differ between cultures.	We can learn and play a game from a different time or culture.

RESOURCES

UFO's: Frisbees and access to other equipment

TEACHER NOTES

All children love to play games. Fun activities that require children to run, chase, throw, jump, dodge, and play not only extend their physical capabilities but also help to develop linguistic, cognitive, and social skills. Making up games helps children to utilise their imagination, make sense of their world, and to have fun experiences with friends. Invented games also allow children to be physically active, improve coordination, and have fun without relying on others to organise them.

Cooperative games encourage teamwork, communication, and creative thinking. However, it is also important for children to be able to take responsibility by learning to play by themselves. The activities in this session will focus on developing both types of games using a novel piece of equipment: the soft frisbee.

CLASSROOM ACTIVITIES

1. UFO'S

- In pairs children explore throwing and catching the frisbee.
- Challenge children to set goals, e.g. to successfully make 20 basic passes and catches. Once they have been successful, challenge them to explore other creative throwing and catching styles (e.g. under the leg, behind the back, etc.).
- Ask children to join another pair to share and practice the different throwing and catching styles they came up with.
- In a mixed group of four (consider gender and ability), children make up a game using the frisbee that can later be shared with other groups.

Groups are to indicate that they have considered the following:

- That everyone is involved and included
- Safety
- Fun

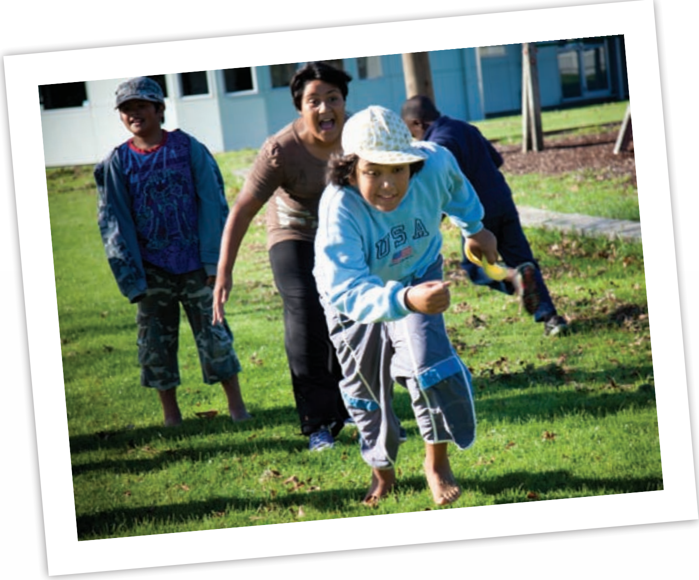

Things to include:

- Number of players (and number of sides)
- Rules (including any scoring) and instructions
- Time limits
- Equipment
- Boundaries
- The name of the game

Prompting questions may include:

- Will people who play this game find it challenging?
- Is the game inclusive? Are children of different ability levels able to participate? (Can you suggest adaptations to make it possible for children with a disability to take part in this game?)
- Can people get better at this game with practice?
- What aspects of the game make best use of the frisbee, i.e. couldn't be done with another piece of equipment?
- What sort of movement skills does this game help with?
- How could teamwork make this game more fun?
- What factors influenced how you created your game?

They may want to test out their game with another group before the end of the session. If you have any spare time during the subsequent Healthy Homework sessions, it could be fun to have children teach their game to the class. Or alternatively, have children record their games with all the information on to card and students can select a game to try out at interval or lunchtime.

SESSION 3: FRUIT AND VEGETABLES

CURRICULUM LINKS

Achievement Objectives	Learning Outcomes:<br>We are learning to:	Success Criteria<br>We will know we are successful when we:
D1<br>Societal Attitudes and Beliefs	Talk about food with others to create an environment that supports healthy food choices.	Have participated and reflected on questions discussed with our family.
A1<br>Personal Growth and Development	Ask questions about how much and what kinds of fruit and vegetables help make us healthy.	Can talk about why fruit and vegetables are good for us.<br><br>We can say why we need different coloured fruit and vegetables in our meals.<br><br>What portion sizes and how often we need to eat fruit and vegetables.

RESOURCES

Table Topics: Cardboard to make table topic cards

TEACHER NOTES

Fruit and Vegetables provide the following nutrients: carbohydrate (for energy), fibre (for a healthy gut), vitamin A, vitamin C, folate, and potassium. Fresh, frozen, canned and dried fruit and vegetables provide nutritional value.	One serving of vegetables is ½ cup cooked vegetables (broccoli, peas, beans) or 1 cup of raw salad. Frozen vegetables are just as good as fresh and often you can get 3 or more different colours and varieties in one packet.
	One serving of fruit is equal to the amount that can fit into the palm of your own hand, e.g. 1 medium sized apple, orange, or pear; 2 small plums or kiwifruit; 1 cup of fruit salad; 2 tablespoons of raisins; 4 dried apricots.

LEARNING NOTES

- 5+ a day: 5 is the number of fruit and vegetable servings that we need to consume each day (two servings of fruit; three servings of vegetables), the plus sign refers to the fact that more is better.
- Fruit and vegetables are highly nutritious.
- The key with choosing vegetables is to select as many different colours as possible to receive a variety of different nutrients. E.g. beta-carotene is found in carrots and pumpkin. Lycopene is found in tomatoes and ruby red grapefruit, lutein occurs in green leafy vegetables such as spinach and silver beet.
- Frozen vegetables are just as healthy as fresh vegetables. They are processed at their peak in terms of freshness and nutrition; therefore they are just as nutritionally good as fresh varieties. In fact they can often be healthier than imported fresh ones sold in the supermarket out of season.

CLASSROOM ACTIVITIES

1. FRUIT AND VEGETABLES

Start the session by explaining to the class why fruit and vegetables are so important for health. The 5+ a day website has some excellent resources to support the following activities: www.5aday.co.nz

1. Explain to the children why eating different colours of vegetables is so important. Complete the vegetable activity below:
2. Divide the class into four groups. Ask each group to:
   - Write down as many fruit and vegetables they can think of beginning with each letter of the alphabet. Ask them to total these.
   - Write down the colour of each of these fruit or vegetables and count up how many types are in each colour.
   - Highlight any fruit or vegetable that all members of the group have never tasted.

Discuss with the group how they can get as much colour into their day as possible. Talk about how frozen vegetables are good as they often come in a variety of colours.

3. Discuss with the class that the requirement of 5+ a day is for everyone, young and old. In pairs, ask the children to devise a fruit and vegetable shopping list for a week (seven days), based on the number of members in their family. E.g. if the family consists of mum, dad, and three children, with each of needing 5+ a day (two fruits, three vegetables), this will amount to 70 pieces of fruit and 105 serves of vegetables.
4. In groups ask the children to discuss and write down some fruit and vegetables that they eat at home that are special to their own culture or country. There may be some varieties that children have not seen before. Ask groups to report back to the class and if appropriate and possible to bring some of these fruit and vegetables into the class.

2. TABLE TOPICS

Brainstorm with the class questions related to fruit and vegetables, e.g. "What is a root vegetable and how many can you think of?" "What fruit or vegetables grow on a vine?", "How do purple fruits or vegetables benefit our health?", "What vegetables are in season now?", "What fruit are in season now?" From the questions listed as a class, children select six questions and create 'table topic' cards to use for homework option A.

This activity relates to Homework Option A

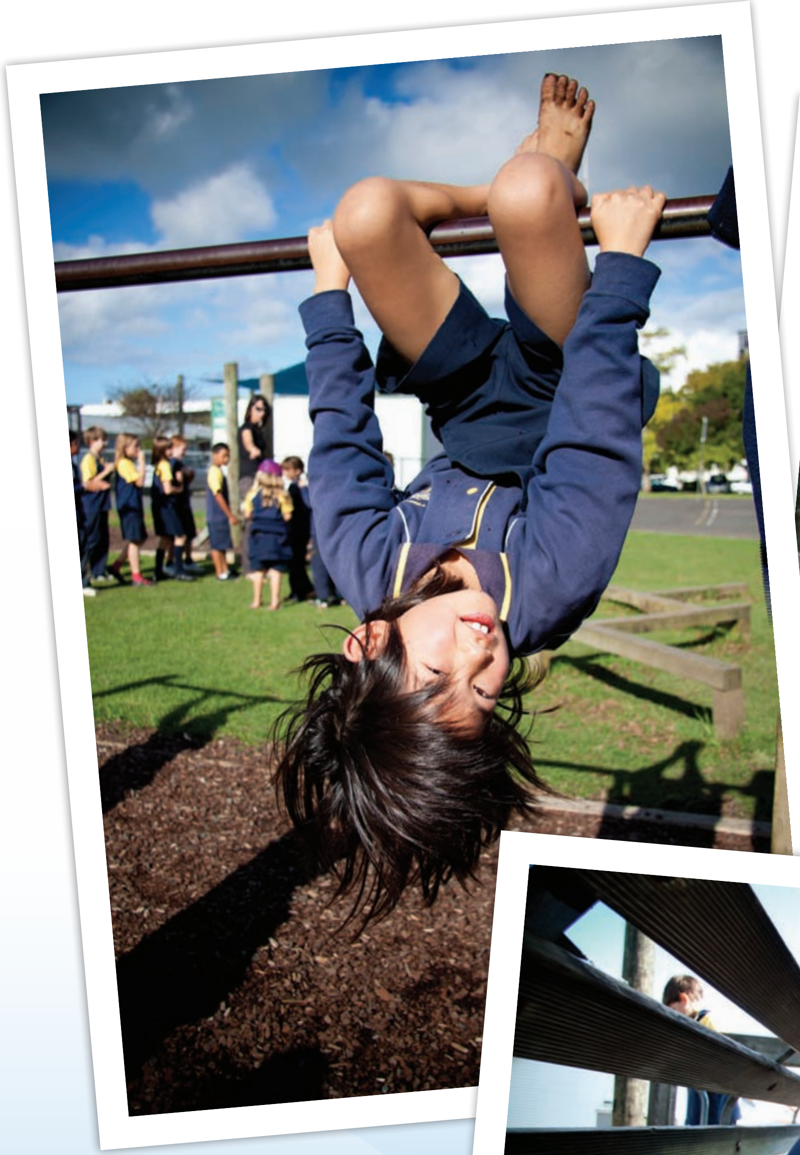

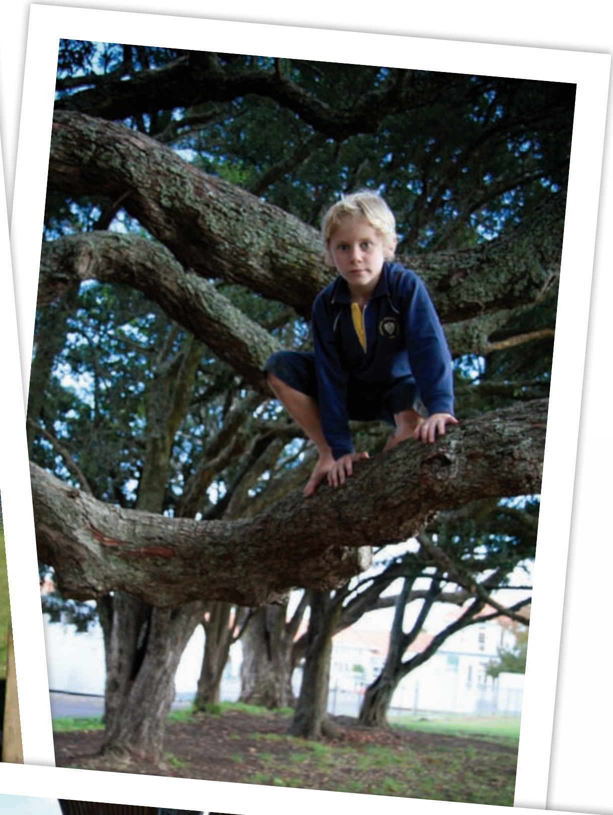

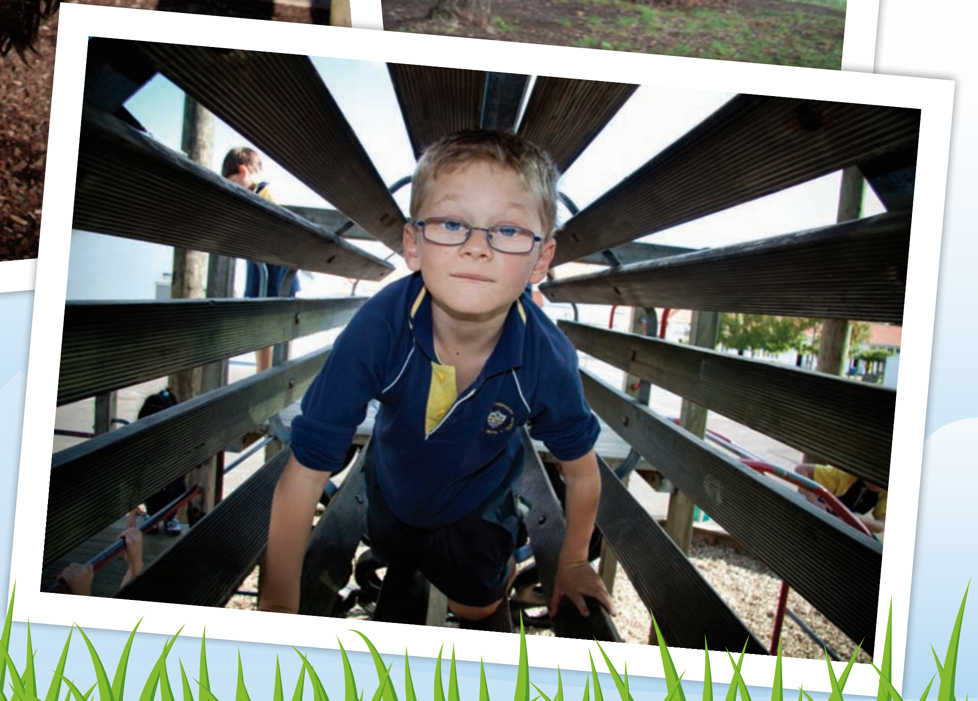

WEEK FIVE

FITNESS / DRINKS

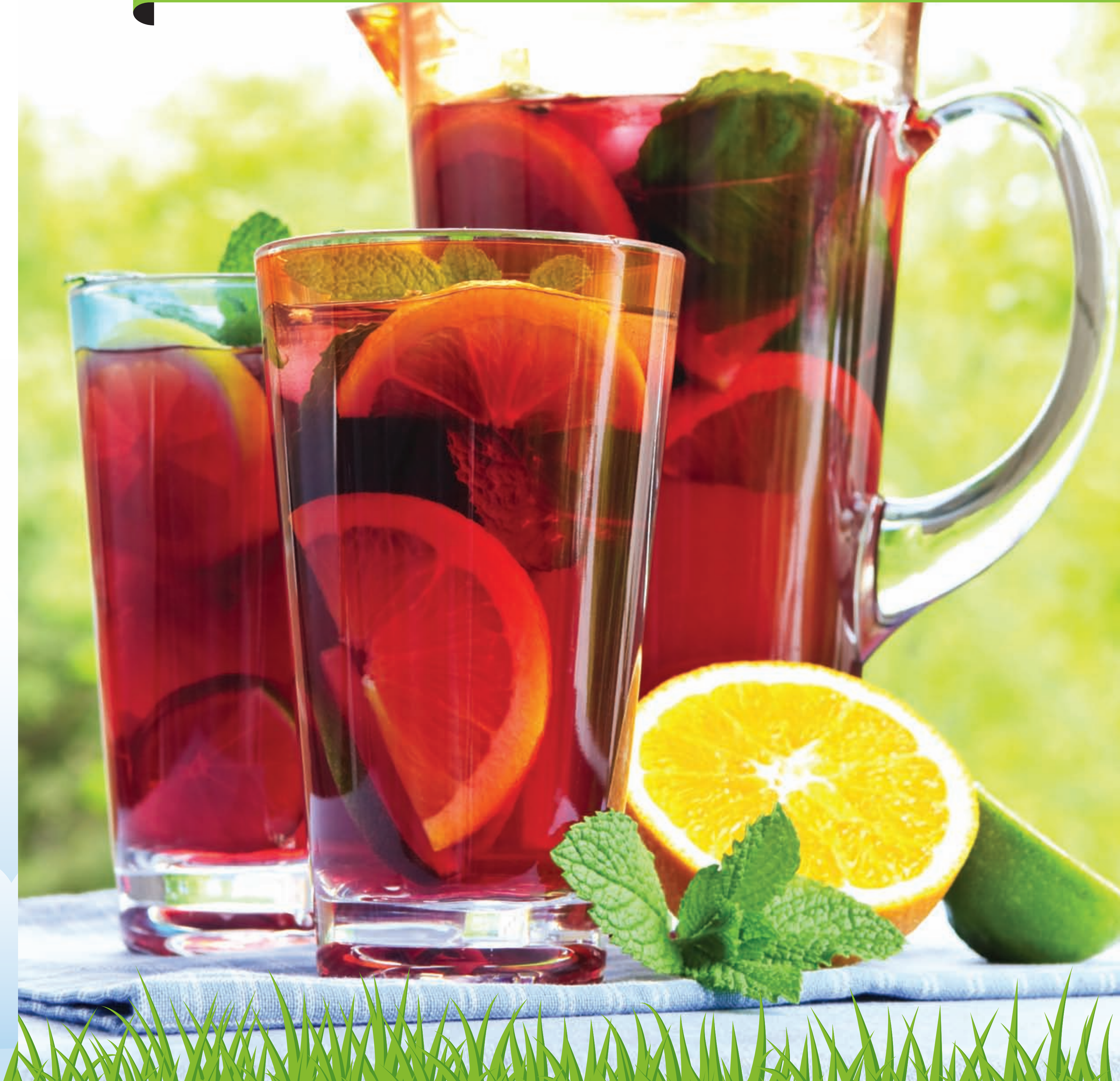

WEEK FIVE

SESSION 1: HOMEWORK REVIEW

GAMES

Go through all three homework options:

Option A: In groups children share the games they created for homework. What were the rules? Did they play their game with their family or friends? Discuss how playing games can affect relationships with other people.

Option B: Ask some of the children to describe the game they played and if possible demonstrate the game to other members of their group. Ask them if there was anything else they learned about this game, e.g. who it was played by, when, or how they learned the game? Is this game unique to a particular community, time, or cultural group and in what circumstances and environments would the game have been played?

Option C: Which skills were used to play the individual game successfully? How did they make sure the game was interesting or challenging? What modifications did they make to the game while they were playing? How did playing a game on their own differ from playing in a group? Which did they prefer and why? Children could teach a partner their game.

FRUIT AND VEGETABLES

Go through all three homework options:

Option A: In groups compare and contrast the answers to the table topic questions. What did children enjoy about this activity? What did they learn? Can children think of any other fruit and vegetable questions that they could take home and discuss over dinner?

Option B: Ask the children if this activity was easy or difficult, and if it was difficult, what some of the barriers were to eating the required servings.

Stand up if you ... this week?

Class sits in a circle facing inward. The teacher asks questions (Have you eaten E this week?) and if the answer to the question is 'yes' children stand up and shout, "I have!"

Example questions:

Have you eaten ... this week, e.g. a potato; jam; peas; raisins; an apple that had a bruise; vegetable soup; a vegetable you didn't like; a new fruit or vegetable; etc.

The teacher could ask some children to explain their experience further or change the action to running around the circle, doing a star-jump, a funny wiggly dance movement, etc.

Option C: Discuss with the class the reasons why we need to include a variety of coloured fruit and vegetables in our diet. Talk to them again about the different vitamins in the coloured vegetables.

SESSION 2: FITNESS

CURRICULUM LINKS

Achievement objectives	Learning Outcomes<br>We are learning to:	Success Criteria<br>We will have succeeded when we can:
A2<br>Regular Physical Activity	Understand how fitness supports our health and our involvement in games and activities.	Complete activities that show we understand the different aspects of fitness and how they relate to wellbeing and to specific physical activities.<br><br>Plan and practice an activity to improve our fitness.
B3<br>Science and Technology	Describe our experiences in different physical activities.	Describe how our body reacts to different activities.

RESOURCES

Move It: Couple Tag instruction card
Tag It: Group Worksheet

TEACHER NOTES

Learning about fitness should involve developing the children's knowledge and understanding of:

- The relationship between intentional exercise and health
- Specific skill-related fitness

"Note that, while fitness is in itself desirable, there are aspects of the fitness culture that may not be." (Ministry of Education 2007. p. 30)
An individual's overall fitness is made up of a number of different components (see chart below).

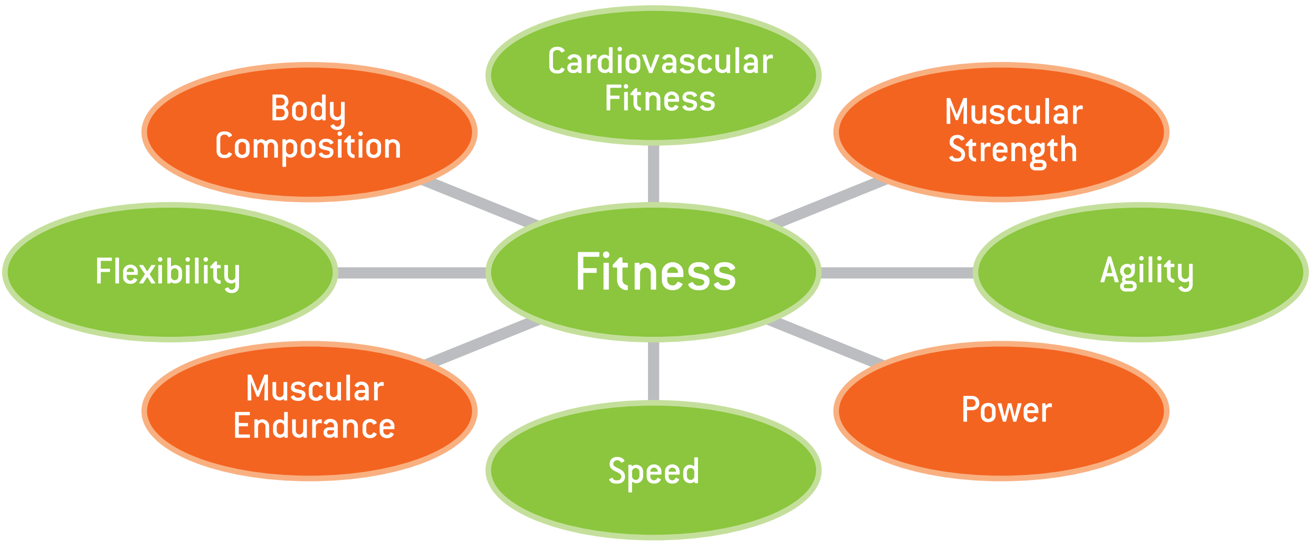

Children require a range of learning opportunities in fitness. These include opportunities to:

- Increase physical literacy, i.e. to develop an understanding of the components of fitness. (In developing this kind of physical awareness or core skills, this may then be transferred to a range of physical activities.)
- Develop an understanding that a person with adequate fitness is able to sustain physical activity or perform physical tasks efficiently without fatigue or injury.
- Increase awareness of influences that shape attitudes to fitness.
- Notice how their body responds as it performs different movements and understand how these responses may change with continued practice. E.g. when an individual begins participating in cardiovascular (huff and puff) activities, they will notice that their heart rate increases, breathing becomes heavier, they may start to sweat and their muscles will begin to fatigue. As a person's fitness increases, they will find that their heart rate will not increase as rapidly and that they will be able to exercise for longer at a given pace before their breathing gets very heavy and their muscles get tired.

Finding appealing ways for children to increase fitness can be challenging. A focus on challenge and enjoyment while participating in a range of physical activities will support motivation.

CLASSROOM ACTIVITIES

1. MOVE IT

Take children outside and lead the following three activities. In between each activity, ask the students to think about how they were feeling towards the activity and how their bodies were responding, e.g. sweat, heartbeat, red cheeks, sore muscles, tightness, etc. Children can be encouraged to feel their pulse. Compare and contrast activities, thinking about the similarities and differences they felt.

1. Play the tag game suggested below or chose another one the class enjoys.
   Couple Tag (Refer Kiwidex Manual. p. 140 SPARC 2007)
   Within a defined area, nominate three couples with inside hands joined as the taggers.
   Instructions: Taggers keep hands joined and tag the free players.
   If tagged, the free player replaces the one who tagged him.
   Continue for several minutes.
2. Guide children through a fast walk, a medium jog, and a fast run.
3. Using the adventure playground, encourage students to spend ten non-stop minutes playing: hanging, swinging, balancing, crawling, climbing, etc.

2. TAG IT

- Explore children's understanding of fitness. What do they know? What have they heard, seen, experienced, etc.? What is the purpose of fitness? Fit for what?

  (Refer to teachers notes ensuring a balance of health related and skill-related fitness ideas.)
- Introduce the components of fitness and have children think of games or activities that would require each component. E.g. netball where agility and speed are needed, as there are many rapid changes of direction.
- Make up eight sets of four cards (there are some provided in the resource box). On each group of four cards write a physical activity, e.g. 4 x tag game, 4 x walk, 4 x medium jog, 4 x fast run, 4 x hanging/swinging, 4 x crawling, 4 x climbing, 4 x balancing. Randomly distribute cards amongst the class. Without talking children act out their physical activity and find their other group members.
- Give a copy of the following worksheet to the group. Ask children to write the name of their activity next to each aspect of fitness that they think their activity uses. Can they think of the purpose? When in everyday life (outside of games and sports) might we use these aspects of fitness? Use the chart below as a guide. Join with another group and share findings.

Aspect of Fitness	Definition	Activity	Purpose: how does this activity use this aspect of fitness?	When in everyday life might these aspects be helpful?
Agility	Rapid changes of direction.	Tag game	To dodge other players and the tagger.	Moving around the house.
Speed	To move the body as quickly as possible.	Tag game	To get away from tagger or to chase and tag others.	To move away from danger.
Flexibility	Stretching muscles.			
Cardiovascular Fitness	Being able to sustain exercise over a longer period of time.	Tag Game	To keep playing for a long time without getting too tired.	To enjoy participation in a range of leisure activities, e.g. running around on the beach, cycling with friends, walking up a big hill.
Balance	Control body movement through body tension.			

SESSION 3: DRINKS

CURRICULUM LINKS

Achievement Objectives	Intended Learning Outcomes<br>We are learning to:	Success Criteria<br>We will know we have been successful when we can
A2<br>Personal Growth and Development	Understand why we need to drink regularly throughout the day.	Describe how fluid helps our body to function and what signs tell us that we need to drink more.<br><br>Name factors that influence how much we need to drink.
	Understand different kinds of drinks and to make healthy choices.	Experiment to find out the sugar content in different drinks and make decisions on how often and on what occasion's different drinks might be chosen.
D4<br>People and the Environment	Plan and take action to promote the drinking of water by children at school.	Explore the barriers and enablers to drinking water at school and take action to create improvements or promote water drinking.

RESOURCES

Sweet As: Sugar, containers, teaspoons
Sample drink bottles, cans, labels, etc.
Traffic light drink worksheet

LEARNING NOTES

- Water makes up approximately 60-70% of your body weight. We lose approximately two litres of water throughout the day via the skin, lungs, bladder, and intestines.
- It is important to ensure that we keep drinking to remain well hydrated. Water is the body's most important component and it cannot go without it for more than about three days.
- We need to drink regularly throughout the day.

TEACHER NOTE:

The exact amount of fluid that we require has been a contentious issue over the years and is subject to much debate. Some researchers say that eight glasses of water per day is not justified by science and that fluid requirements vary between individuals, depending on numerous factors; body size and sweat rates being two of these factors. It is therefore suggested that healthy individuals require a baseline of approximately eight glasses of total fluid per day, rather than water alone. Naturally, more fluid is needed when physical activity is incorporated into the day. Total fluid does not mean only water, but also includes the fluids listed in the table below.

TYPE OF BEVERAGE	DESCRIPTION OF BEVERAGE	DRINKING GUIDELINE
Sports drinks	Contains carbohydrate in the form of sugar (4-8%), contains sodium and potassium. E.g.: Powerade, Replace	Useful to consume during or after long and intense exercise sessions (lasting more than 1 ½ hours). For most children sports drinks are not necessary and due to the high sugar are not recommended.
Caffeine-containing drinks	Contains carbohydrate in the form of sugar. Contains caffeine. E.g.: Red Bull, cola	Caffeine can contribute to dehydration, can be addictive and can cause heart rhythm irregularities in some people. Caffeine containing drinks are not recommended for children.
Carbonated beverages (soft drinks)	Carbonated drinks are often high in sugar and additives. E.g.: Sprite, ginger beer.	Should be kept to a minimum. Diet options are a good option when choosing soft drinks but should also only be consumed "occasionally."
Sports water / vitamin drinks	Contains carbohydrate in the form of sugar (2.5%), may contain some vitamins or minerals. E.g.: Mizone	Good for people who do not like water but who do not want to consume as many calories as in a sports drink or juice. Extra vitamins or minerals often present in these drinks do not provide any extra benefit if your diet is well-balanced.
Fruit juice / cordial	Contains carbohydrate in the form of sugar (8-12%), contains potassium.	These drinks are very high in sugar and should only be used sparingly. They can be used as a treat or in special cases (to add energy if a lot of exercise is being carried out throughout the day).
Milk-based drinks	Contains carbohydrate, protein, calcium. E.g.: Flavoured milk, Up & Go	Use during the day to obtain healthy nutrients as well as fluid. Choose low fat varieties. Restrict servings of flavoured milk.
Water	Contains no carbohydrate (CHO), vitamins, or minerals.	The healthiest drink. Use throughout the day to maintain hydration levels.
Non-fat milk	Contains carbohydrate, protein, minimal fat, vitamins and minerals (particularly calcium).	Use throughout the day for a drink or on cereal.

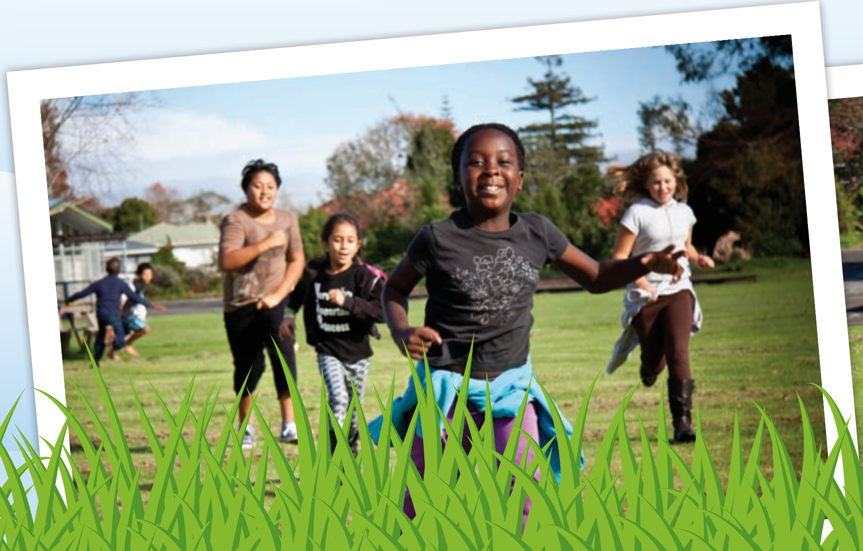

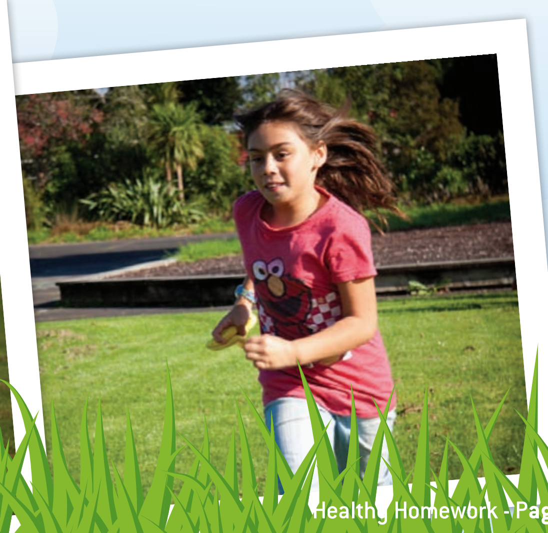

➲ CLASSROOM ACTIVITIES

1. THE DRINKS ARE ON ME

In pairs, groups, or as a class children consider the following questions:

1. Why is it important to drink enough fluid?

   Regulates body temperature, lubricates joints and moistens tissues; helps prevent constipation and is involved in many metabolic reactions.
2. What are the signs of dehydration?

   Thirsty, decreased urination, highly concentrated urine, dry skin, lips, irritability.
3. What factors influence the need for increasing the amount of fluid we drink?

   High temperatures, vomiting, diarrhoea, excessive sweating, physical activity.
4. What factors might need to be considered before working out how much someone should drink each day?

   Size, age, activity level, medical history.
5. Why might it be better to drink regularly through the day rather than drinking a whole lot at one time?

   Fluid is better absorbed into the body, too much too fast often results in many trips to the bathroom.

It is suggested that children drink 6-8 glasses of fluid a day.

6. How much do they drink in a day?
7. Do they have a drink at lunchtime?
8. What kinds of fluids do they drink?

2. THIRSTY WORK

Ask the class what makes it easy or difficult to drink an adequate amount each day? E.g. time, availability, taste preference, something to drink out of, climate influences, physical activity levels, etc.

In groups children walk around school investigating and recording the number, position, and state of repair of the drinking fountains.

Draw conclusions from your findings:

- Adequate number versus pupil numbers.
- Are they positioned in suitable and inviting places around the school (near playgrounds, fields, fairly distributed between junior/senior schools?
- About the condition (cleanliness, function).

If groups feel improvements are needed, children could make suggestions to the School Council or Principal.

Can the children think of other ways to promote water drinking in the school? E.g. children could develop key messages and create a poster on ways of taking health-promoting action. Post a drinks communication blog on the Healthy Homework website.

3. SWEET AS

Divide the class into groups. Assign a drink label to each group, e.g. coke, V, cordial, juice, etc. (an assortment is provided).

1. Ask the group to calculate how many teaspoons of sugar are in the drink they have been given.

   1 teaspoon = 4.2 g sugar

   If possible get them to measure out the different quantities of sugar in each drink.
2. Why do they think drinks contain so much sugar? How would they describe the taste of sugar to someone? What effect do they think sugar has on the body, where does it fit in the food groups and for what reasons?
3. Discuss the pros and cons of playing sports and drinking high sugar drinks.

Provide each group with an outline of the beverage traffic light system (master copy in resource box). Ensure that the children understand the vocabulary.

1. Have children record examples of different drinks in the 'description' column.
2. In the drinking 'guideline' column, groups record their initial thoughts on who might drink this beverage, how often, and for what purpose.
3. Read out, show pictures, or display a variety of different drink bottles. Ask the children to write the name of each drink under the traffic light colour where they think it best fits. Share outcomes and discuss reasons for the placement of different drinks.

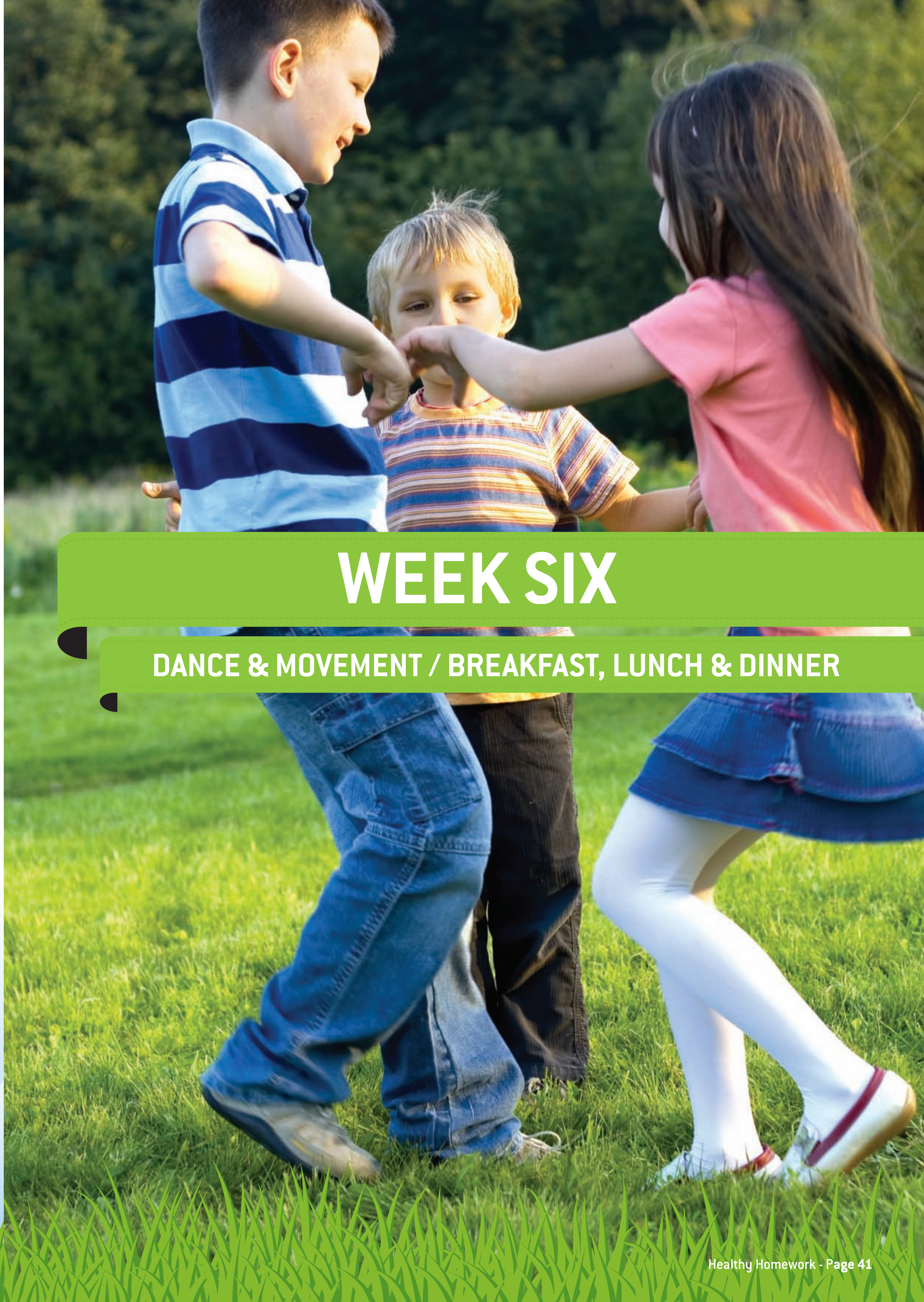

WEEK SIX

SESSION 1: HOMEWORK REVIEW

FITNESS

Go through all three homework options:

Option A: Ask children to share their activity with a partner and explain its purpose. Schedule time for children to blog their activity on the website. Briefly include the following: the sport, skill, activity they created, answers to homework questions.

Option B: Have the children share their thoughts and feelings about this activity and discuss the contribution of fitness to wellbeing.

Option C: Discuss what the children liked about the circuits they created. Ask some of them to demonstrate the exercises that they incorporated into their circuit to members in their group. Have them compare the circuit to running – how was it the same or different? Discuss why they think it is good to try different fitness activities, i.e. a variety which works different muscle groups. What are the links between how we feel during exercise and how this feeling affects our decision or motivation to exercise again?

DRINKS

Go through all three homework options:

Option A: Discuss how much water they drank most days and ask children why they need extra fluid when they do physical activity.

Option B: Are drinks considered food? Discuss the importance of nutrients provided by milk (protein, carbohydrate, calcium) and how as a result milk-based beverages can be considered food.

Ask children to share findings and thoughts on sport and fizzy drinks. Can we consider them as food? Why or why not?

Fizz Pop (modified Bump, Kiwidex. p. 157. Sparc 2007)

Formation: Children stand in freely spaced pairs, side by side with elbows loosely linked. Two children stand free.

Instructions: One freestanding child is the tagger. The other child is the runner. Tagger chases the runner, if runner is caught and tagged they immediately reverse the roles and the runner chases the tagger. The runner can at any time avoid being caught by joining onto a group of two, linking elbows with an end person. When this happens, the child on the other end of the trio is bumped off and chased by the tagger.

To make the game relevant to the 'drinks' topic, explain to children that sugar gives us short bursts of energy but then we often feel exhausted after a short time. Tagger and runner are sugar loaded. When the runner links elbows with another pair, the trio jumps up and down (to imitate a fizzy drink being shaken). The player at the end then pops off similar to what happens to the top of a fizzy bottle after the drink has been shaken.

Option C: What factors influence the choice of drinks of your family members? Are these similar or different to the factors that influence your own choice of drinks throughout the week?

SESSION 2: DANCE AND MOVEMENT

CURRICULUM LINKS

Achievement Objectives	Learning outcomes<br>We are learning to:	Success Criteria<br>We will know we have been successful when we can:
3A2<br>Regular Physical Activity	Participate in dance activities, understanding how it contributes to our hauora/wellbeing.	Think of and write how dance might relate to our physical, social, mental/emotional, and spiritual wellbeing.<br><br>Participate in planned and unplanned dancing and say what was enjoyable about each.
3B2<br>Positive Attitudes and Challenge	Put a range of dance movements together and show others what we have learned.	We can confidently practice specific dance moves and make a dance sequence.<br><br>Share with other people some unfamiliar dance movements from other cultures.

RESOURCES

Boogie Down: Dance CD
Hauora worksheet

LEARNING NOTES

- Dance encourages movement, expression, and creativity. Movement through dance can be a fun and enjoyable way to increase physical activity, motor skill development, as well as personal and social development. Some of the physical benefits of dancing include improved strength, balance, and coordination.
- Dancing incorporates a number of individual moves that are linked together in a sequence. Movement in dance includes jumping, bouncing, hopping, sidestepping, squatting, stomping, arm swings, twirls, and turns.
- In different cultural and ethnic groups, dance is used for entertaining, reflecting on spirituality, telling stories, and for enjoyment.
- Today, different forms of street dancing are very popular. Breakdancing, hip-hop, and krumping are examples of popular forms of street dancing and have common elements including their "street" origins.

Hip Hop	Breakdancing	Krumping
Hip hop is a generic term and can refer to different styles such as breakdance, popping, locking, funk, high energy jazz, and street dance.<br>Popular in pop culture and can be seen in many music videos.	One of the significant dances of hip-hop culture, mainly performed to hip hop music or other genres, often remixed.<br>Contains different techniques, including standing moves, moves on the floor, aerobatic moves, or freezes.<br>While some breakdancing moves rely on body control and balance, it also trains the ability to isolate movements.	Freestyle dancing, containing big, energetic, wild movements of the whole body.<br>Styles include wobbles, chest pops, arm swings and stomps.

CLASSROOM ACTIVITIES

BOOGIE DOWN

- What is dance? (Refer to teaching notes.)
- Children think about the different types of dancing they know of. (You can use the links on the Healthy Homework website to show them some different types of dancing.)
- Children demonstrate and brainstorm dance moves in small groups and then compile a list of the moves on the board. (It will be a good idea to keep this list on the board for the children to refer to when they are designing their own dance activity.)
- In groups of three to four, ask the children to think of and link at least six different movements together to create their own dance. (Use music provided.) Have children consider elements of body, space, time, speed, energy and relationships, and level. If time allows, children could share their dance with other groups.
- Children brainstorm the possible health benefits of dancing using the four interdependent dimensions of hauora.

Taha tinana/physical<br>E.g. coordination	Taha wh nau/social<br>E.g. dancing with friends
Taha hinengaro/mental and emotional<br>E.g. expressing my feelings through movement	Taha wairua/ spiritual<br>E.g. strengthening personal identity

Preparation for homework option B

- In groups, children select a country whose traditional dance they would be interested in learning about.

This activity relates to Homework Option B

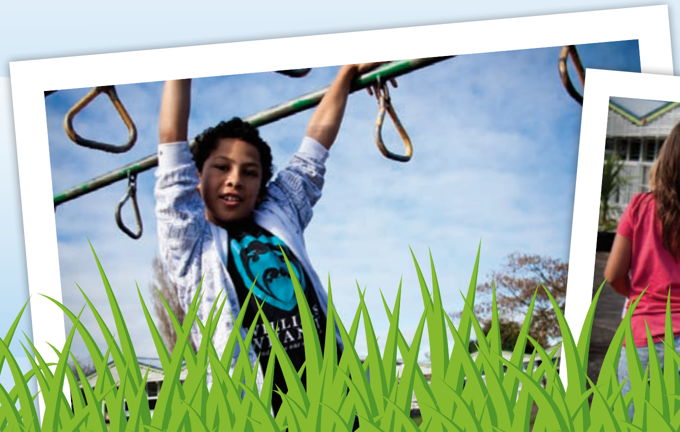

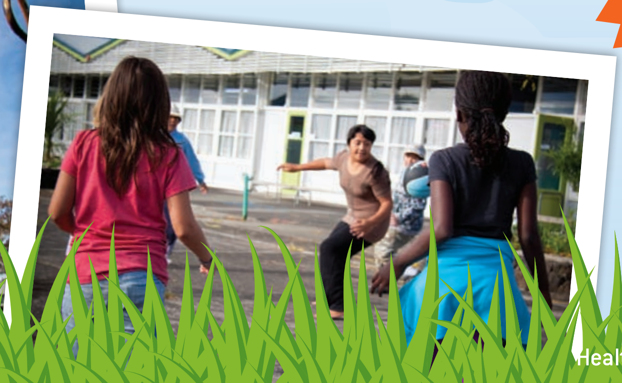

TEACHER NOTES

Children may choose their own culture, a dance culture that someone is familiar with (e.g. highland, Irish dancing, or kapahaka), or a country they know nothing about. Refer to homework option B.

SESSION 3: BREAKFAST, LUNCH AND DINNER

CURRICULUM LINKS

Achievement Objectives	Intended Learning Outcome<br>I am learning to:	Success Criteria<br>We will know we have been successful when we:
3A1<br>Personal Growth and Development	Identify reasons why it is important to eat a healthy breakfast and lunch.	Know how much and what kind of food is important to have for breakfast and why.<br><br>Can make simple suggestions to improve food choices and take action to inform others.
3D2<br>Community Resources	Help with planning and preparing meals and to describe how these activities contribute to family wellbeing.	Identify nutritional needs and what foods and quantities make up a balanced diet.<br><br>Make helpful suggestions to improve the nutritional value of different meals and mealtime experiences.<br><br>Plan and help with the preparation of dinnertime meals.

RESOURCES

Healthy Start: Fibre Cards

LEARNING NOTES

Breakfast

Breakfast is reported to be the most important meal of the day.

- In general, children who eat breakfast tend to have more energy, eat healthier throughout the day, and tend to concentrate better in school.
- Without breakfast, children can get irritable, restless, and tired. Their mood and energy can drop by midmorning if they don't eat at least a small morning meal. In the morning, their bodies need to refuel for the day ahead after going without food for eight to 12 hours during sleep. The breakfast meal breaks the fast and increases the metabolic rate for the day ahead.
- The importance of breakfast is not only about eating something, but is also about the quality of the breakfast meal. A healthy balanced breakfast can provide a quarter of the recommended daily allowance for a variety of nutrients and at least a third of daily fibre needs.

Breakfast ideas include:

- Wholegrain cereals (Weetbix/All Bran/muesli) or porridge + low fat milk or yoghurt
- Wholegrain toast + healthy spreads such as avocado/peanut butter/low fat cheese. Adding a glass of milk or yoghurt to this meal will help boost calcium intake
- Chopped up fruit with yoghurt
- Liquid breakfast (e.g. Up & Go) and fruit for those who are on the run
- Eggs

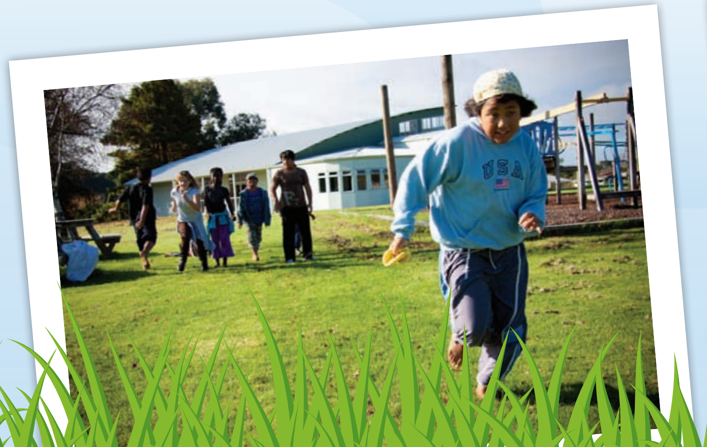

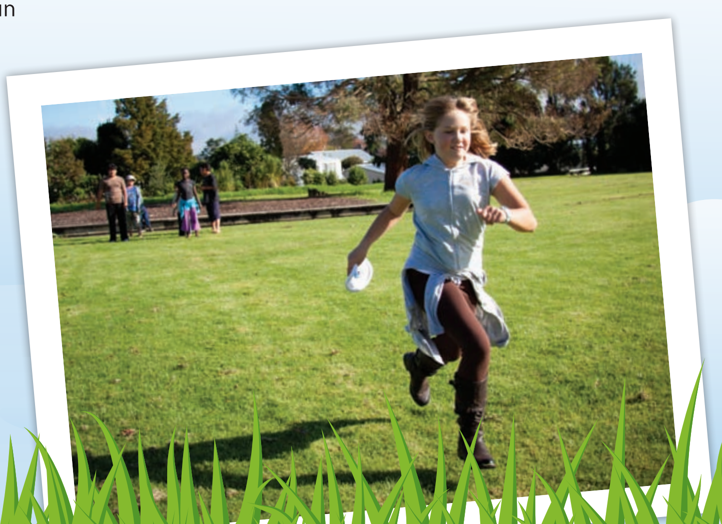

Lunch

In order to maintain the benefits of a good breakfast and support further concentration through the day, nutritious school lunches and snacks are important.

- Wholegrain sandwich/wrap/roll/pita bread, with lean meat/skinless chicken/shaved ham, and salad, PLUS fruit/yoghurt, AND water.

Dinner

Evening meals are important for many reasons. As well as re-energising the body it may be a time for families to come together and share their day.

Typically a healthy evening meal includes the following components:

- Meat, fish, dairy products (protein for muscle and bone growth)
- Potato, bread, rice, noodles (carbohydrate for energy)
- Vegetables (vitamins, minerals, and fibre)

Being involved in the selection and preparation of food helps children to learn about nutrition and healthy eating and develops skills and good eating patterns to support them through life.

Takeaways can be both a healthy and unhealthy option. The following options are considered healthy takeaways:

- Sushi
- Thai food (without coconut cream sauces or battered items)
- Wraps (without creamy sauces)
- Thin-based pizza with minimal cheese, a mix of vegetables, and lean meat-based toppings

CLASSROOM ACTIVITIES

1. HEALTHY START

Introduce the concept of consuming a healthy breakfast meal each day as well as the concept of fibre, explaining its benefits.
Using a variety of breakfast cereals, e.g. Weetbix, rice bubbles, cornflakes, porridge, Nutri-grain, Coco-pops, ask the children to fill a breakfast bowl with the average serving of each cereal. (Use scales to measure).
Children can calculate how much fibre they need in a day. To work this out, they can take their age in years and add 5. (E.g. a 10-year-old will need 15 grams of fibre daily).

Transferring the chart below onto the board, find out the following:

Cereal	Average serving size	Fibre content of serving	Children's daily fibre need divided by 3.	Is my breakfast meeting my fibre needs?
Weetbix				
Cornflakes				
Coco-pops				

Using the cards provided, have children create two types of breakfasts (one that is high in fibre and one that is low in fibre). Ask them to total the amount of fibre in each breakfast. Then get them work out what the percentage is (of each breakfast) of their daily fibre requirement.

2. LET'S EAT LUNCH TOGETHER

Plan a shared healthy lunch day.

Children brainstorm what comprises a balanced healthy lunch. Refer to list above. Divide the class into groups and each group selects a component from this list, e.g. fruit, drink, sandwich, snack, yoghurt, etc. (If this component is difficult for any student to bring, they could swap with another.) Choose a day for the shared lunch on which the children bring food to share and place it on the table for viewing. Discuss the variety and health benefits of different foods. Children celebrate diversity and enjoy sharing the food and eating together.

3. EATING TOGETHER

How might sitting down to a healthy meal with family members support our wellbeing?

As a class children identify any not so good things about dinner times.

What would help children's 'not so good' experiences become more positive? Have children think of possible solutions.

Draw three columns as shown:

No so good	Possible solution	Positive
Hate eating peas	Negotiate the amount I need to eat	Less arguments at the table
Dad wants to watch the news and tells us to be quiet	Suggest we eat dinner before the news	Allowed to talk at the table.

Discuss any other factors that influence our experiences at dinnertime.

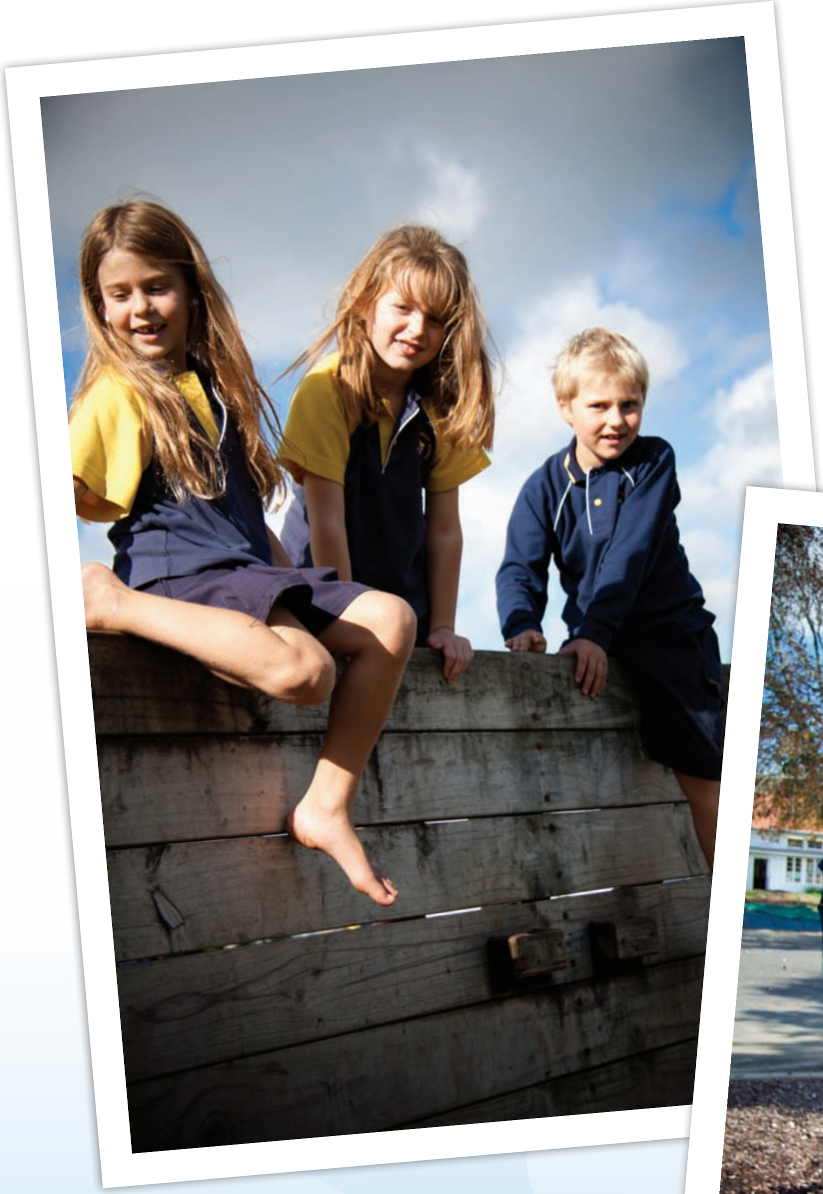

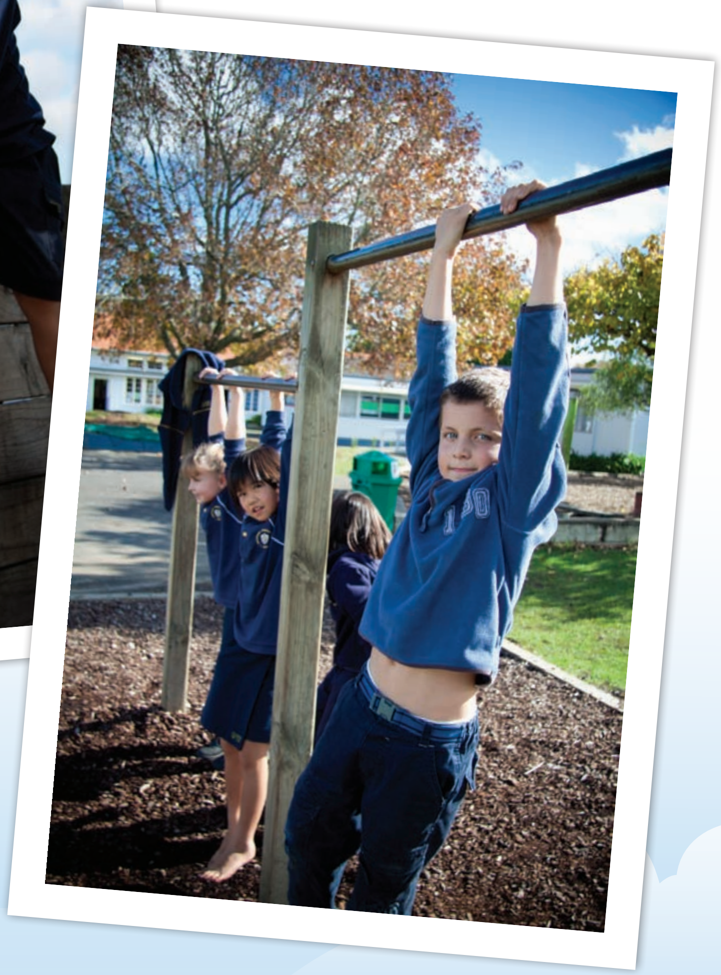

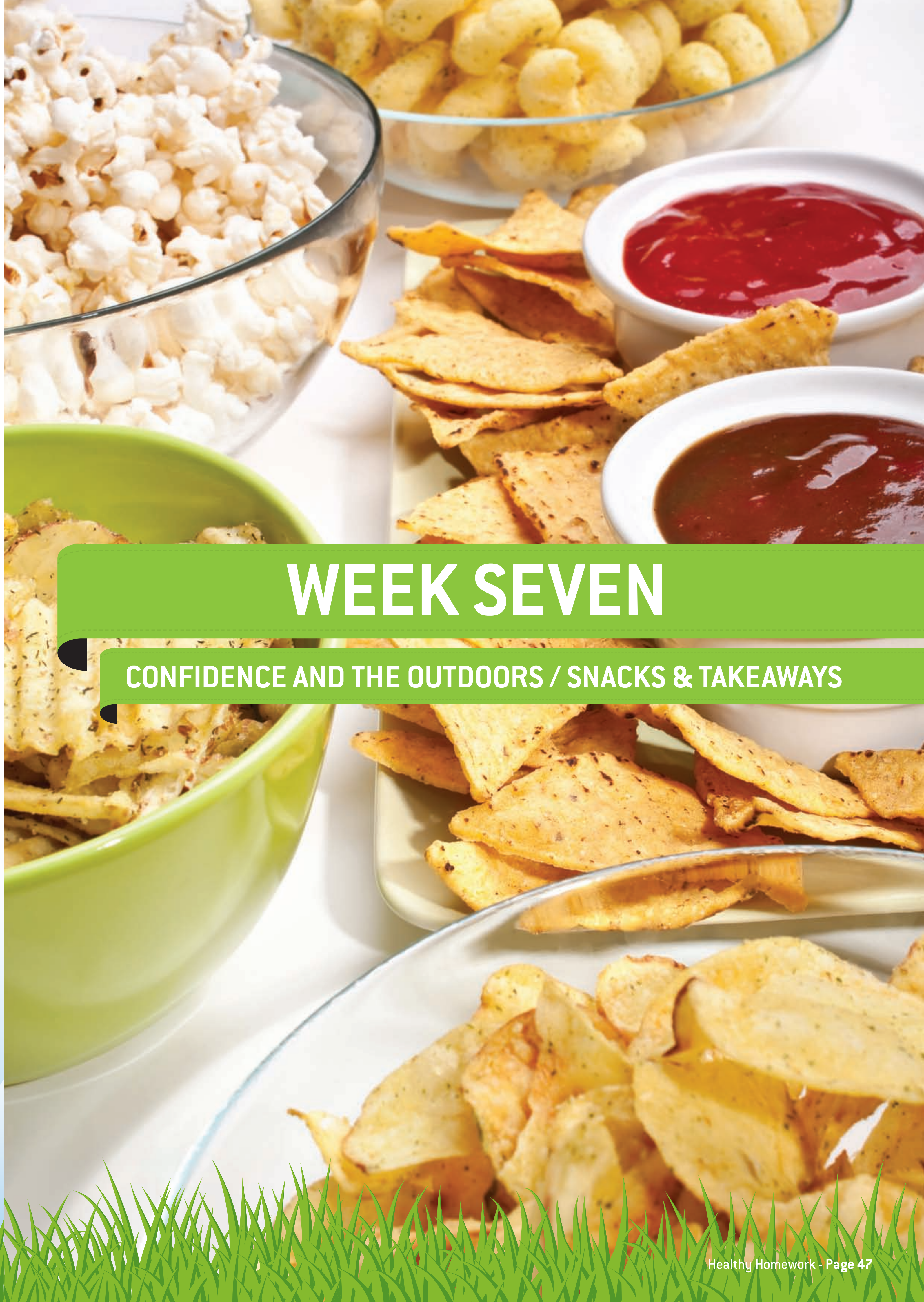

WEEK SEVEN

CONFIDENCE AND THE OUTDOORS / SNACKS & TAKEAWAYS

WEEK SEVEN

SESSION 1: HOMEWORK REVIEW

DANCE AND MOVEMENT

Go through all three homework options:

Option A: In small groups, children can share their dance moves with each other. In their groups they can discuss the challenges they faced when creating the dance moves. How did they overcome these challenges? Did they enjoy performing their dances to their families? Why or why not?

Option B: Ask the children to share what they learned about the cultural dances they researched. What do they think the purpose of the dance is, i.e. it to tell a story, a spiritual ritual, a war dance, or entertainment? There may be some children that want to perform their dance to the class.

Option C: Discuss how it felt to dance freestyle. What were the differences children experienced when free dancing as compared to structured dancing? Put some popular music on and let the children show their pop star moves.

BREAKFAST, LUNCH AND DINNER

Go through all the homework options:

Option A: In groups, ask children to discuss the foods they selected for each lunch scenario. There may be restraints to selecting healthy options, so ask them to explain why they made specific selections and why or why not they may have chosen some foods. Ask children to talk about the types of foods which were appropriate for different family members and which foods could be included in most of the lunch scenarios.

Option B: What suggestions did the children come up with to create even healthier breakfasts? Has anyone tried making these changes?

Option C: Ask volunteers in the class to select one meal they helped prepare and present what it was to the rest of the class. Discuss with the class the benefits of helping to prepare meals with other family members, e.g. spending time with mum or saving dad some time by helping him in the kitchen. Ask volunteers in the class to describe the new meal that they were involved in cooking. Ask whether or not they enjoyed the new tastes and whether or not they would want to try more new recipes.

SESSION 2: CONFIDENCE AND THE OUTDOORS

CURRICULUM LINKS

Achievement objective	Learning outcome<br>We are learning to:	Success Criteria<br>We will know we are successful when we
3D1<br>Societal Attitudes and Beliefs	Find out ways our local environment provides opportunities for outdoor physical activity.	Explore the local environment and identify specific features or places where we can be physically active.
3B2<br>Positive Attitudes and Challenge	Be confident and cooperative in challenging adventure activities.	Work with other team members to effectively solve problems.<br><br>Successfully and safely guide our friend or family member around an obstacle course.

RESOURCES

In the Neighbourhood: Map of local area
T.E.A.M: Problem solving activity cards

TEACHER NOTES

Learning in the outdoors "provides children the opportunity to develop personal and social skills, to become active, safe, and skilled in the outdoors and to protect and care for the environment" (p.46. 1999, Ministry of Education).

The outdoors is not only a great place to build confidence and skills, but it is also great place to be active. In New Zealand we are fortunate to have access to many local, regional and national parks, reserves and beaches.

Suggested teaching points:

- Overcoming challenges
- Assessing risk
- Improving confidence when in the outdoors

CLASSROOM ACTIVITIES

1. IN THE NEIGHBOURHOOD

Begin the session by facilitating a discussion on being active in the outdoors.
Ask the children to identify areas where they can be active in the outdoors. Have children Google their local area around their home.

1. On the map mark places that the children frequent, e.g. school, supermarket, sports field, local park, friend's house, church, doctors, etc.
2. From their own house have children draw a line to indicate how far they are allowed to roam unsupervised by themselves.
   - What do they think are the reasons for this?
   - What are the benefits of playing in the neighbourhood?
3. Mark a second line to indicate where they can explore with a friend but still unsupervised.
4. Thirdly, mark the area they would be able to roam with an adult.
5. Children can investigate well-known tracks and outdoor activities in the local region and plot them on their map. Indicate which one they would most like to participate in.
6. Next investigate those outdoor activities available further afield.

2. T.E.A.M. (TOGETHER EVERYONE ACHIEVES MORE)

Group activities

- **A to B**

  The aim is for a group of three to get from point A to point B without talking.

  In groups of three, the children stand one behind the other. The person at the back of the line is the driver. Without talking to other group members, the driver decides where Point B is. Using a non-verbal code (decided prior by the team, e.g. using shoulder taps etc.), the driver steers the group to the destination. Change drivers.

  Skills practiced: leading, following, trust, confidence, and communication.

- **Knot for Long**

  The aim is to untangle the human knot.

  In groups of 7-10, children stand in a circle with their shoulders touching. Have everyone put their left hand in the middle and hold hands with someone in the circle, not directly next to them. Repeat with the right hand and be sure to hold hands with a different person, who is not directly next to them. The aim is to use teamwork to untangle the knot into a circle again without becoming disconnected! It may take awhile to begin with. Students should be encouraged to contribute their ideas on how to untangle the knot.

  Skills practiced: decision making, problem solving, and communication.

- **Magic Carpet**

  Ask a group of 7-10 to stand on a 2.4 m x 2.4 m (approx.) "magic carpet" (tarp, groundsheet). The entire group must be on the tarp completely. Once everyone is settled, advise the group that they are going on a magic carpet ride. Tell them that they have raised 30 metres in the air and are ready to go.

  Unfortunately, the instructions on how to steer and land the carpet are on the other side of the carpet. So, they must flip the carpet over while standing on it.

  Variation: Once the group successfully completes the above task and they are safely back on land, advise them that you've received a report that another group needs half of their magic carpet to keep their feet warm. Ask them to get off, fold the blanket in half, and fit all group members back on the "carpet" again.

  Repeat the above process as many times as possible. Each time you fold the blanket, the group will need to be more creative (and cosy) to successfully stand on the blanket.

  Skills practiced: decision making, problem solving and communication.

- **Find and Fetch**

  The aim is to use map reading skills and work as a team to place and gather objects from around the school.

  Give each group (of five) a map of the school and five objects of the same colour (cones, discs, laminated cards, etc.) Set a timeframe (e.g. 10 min). Each group runs out into the school grounds with a map and their five objects. They position (not hide) the objects in places of their choice and mark the position of each object on the map. They return the map to the teacher. The teacher distributes each map to a different group. Give students a few minutes to work out a strategy to run, locate, and gather the five coloured objects as quickly as possible.

  (Each map should indicate the colour of object the group is looking for.)

  When each team has returned to the room, discuss team strategies for success. If time allows, each group could be given another different map and a set of cones. Reading the map, the group work out and position the objects in the correct place for another group to locate.

  Skills practiced: communication, problem solving, teamwork, using tactics, map reading.

SESSION 3: SNACKS AND TAKEAWAYS

CURRICULUM LINKS

Achievement Objective	Intended Learning Outcome<br>We are learning to:	Success Criteria<br>We will know we are successful when we can:
A1<br>Personal Growth and Development	Be aware of different feelings in our bodies and make healthy choices to meet our physical needs.	Identify what signals hunger and fullness.<br><br>Read the nutrition labels of different snacks and takeaways and make healthy choices.

RESOURCES

Snack Attack: Food labels (muesli bars, lollies, chips, etc.)
How to read a label information sheet

TEACHER NOTES

Hunger – one of our body's strongest and most beneficial stimuli – helps ensure that we consume enough calories for our needs. It is logical that the only way to end hunger is to eat. Eating provides satiety – the sensation of feeling full – with some foods being able to provide a greater level of satiety than others. Generally foods that contain protein and fat tend to make us feel fuller than carbohydrate-based foods. A serving of baked beans for example are more likely to "fill you up" much better than a serving of lollies that has the same number of kilojoules. Satiety can have an impact on how we feel after a meal and influence snacking habits later on. In general, the more satisfying a food feels, the more effective it proves to be as a deterrent to nibbling later on.

LEARNING NOTES

- Children need healthy snacks in between breakfast, lunch, and dinner.
- Children's stomachs cope better with smaller meals more often.
- It is good to be aware of what, when, and why we are snacking to maintain good health.
- Nutrient-rich snacks are good, and we need to be thoughtful about snacking on energy-dense, nutrient-poor snacks.
- Snacks should be viewed as small mini meals, to bridge the gap between main meals.

Examples of good snack foods
The most convenient lunch box snacks are often pre-packed, whether naturally (i.e. fruit skins) or by a food manufacturer. See below for some examples of nutrient rich snack foods:

- Whole fruit: apple, pear, banana, etc.
- Small plastic container of dried fruit, nuts or seeds, or a mix of these
- Box of raisins
- Liquid meals in tetrapaks (high energy)
- Pot of fruit salad (buy shelf-stable pots)
- Rice cakes
- Pottles of yoghurt

Good quality snacks add to main meals to provide our bodies with a well balanced, nutrient-rich diet. Such a diet will ensure optimal energy levels for learning and physical activity and for wellbeing.

Often people confuse snacks with treats. Typical treat foods include foods that are high in fat, sugar, and salt and examples include the following:

- Chocolate/confectionary (lollies/sweets)
- Pastry/cakes
- Biscuits
- Chips
- Ice-cream
- Some muesli bars (chocolate coated)
- Fast foods (KFC, McDonalds, and Burger King combo meals)
- Fizzy drinks

If you are unsure as to whether a food is classed as a snack or a treat, the best thing to do is to read the food label and then decide. These foods should not be consumed on a daily basis, but rather on a special occasion.

Snack Sorter
The following table will help to identify healthy snack items. Snacks are very important for growing children and young people. Foods chosen for snacks should come from the four food groups; they should be nutritious and low in fat, salt, and sugar.

E = EVERDAY **S = SOMETIMES** **O = OCCASIONAL**

FOOD PRODUCTS	E	S	O	COMMENTS
Milk-based snacks:				Milk-based snacks contribute to children's intakes of protein and calcium.<br>Choose reduced- or low-fat products.<br>Try serving these foods with fresh, frozen, or canned fruit.<br>Try freezing yoghurt or custard for a summer snack.
yoghurt	*	*		
soy yoghurt	*	*		
custards		*		
dairy desserts		*	*	
creamed rice		*		
Sweet snacks:				Sweet snacks are often high in saturated fat and kilojoules (energy).<br>Choose carefully from this food group, and keep package and canteen serving sizes to a minimum.<br>Some sweet snacks contain added confectionery (e.g. chocolate coating), which contributes to excess kilojoules (energy).<br>Choose biscuits and cereal/muesli bars with fruit or wholegrain cereals to increase fibre intake.
biscuits			*	
breakfast bars		*		
cereal bars		*		
baked fruit bars		*		
popcorn		*		
Savoury snacks:				Some savoury snacks are high in saturated fat and salt. Choose carefully from this food group.<br>Wholegrain and other high-fibre and low-fat crackers are better choices.<br>Top crackers with food products such as vegetables, cheeses and meats from the everyday category.
water crackers		*		
corn thins		*		
rye crackers		*		
rice crackers		*		
crispbread		*		
potato chips			*	
corn chips			*	
vege chips			*	
rice cake/wheel		*		
cracker and cheese packs		*		

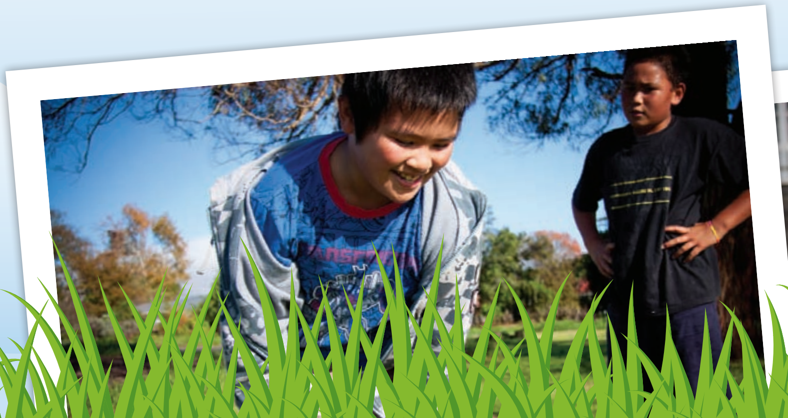

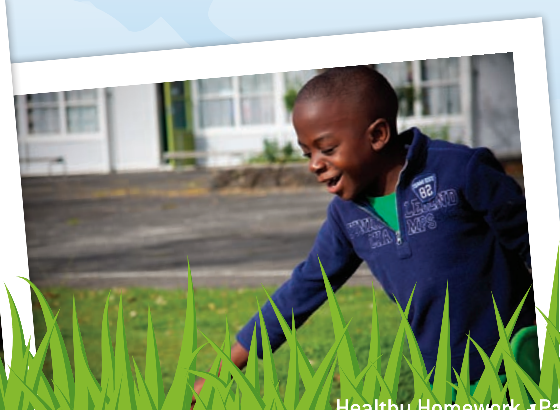

CLASSROOM ACTIVITIES

1. HUNGER PANGS

1. With a partner ask the children to share some cues their body gives when they are feeling hungry or full.
    a. Hunger: discomfort, weakness, headaches, dizzy, low energy, gurgling, empty, irritable, grumpy.
    b. Full: stomach extends, no longer want to eat, either comfortable or if eaten too much uncomfortable and sick.
2. Ask children to stand on a continuum depending on how hungry or full they feel, 1 being completely ravenous and 10 being overfull.
3. What factors affect our feeling of hunger or fullness? E.g. time between meals, type of food last eaten, and physical activity.
4. Ask the children to think about the last meal they had and how long ago they had it (food and fluid.)
5. Ask children to consider the amount of physical activity they have completed since this meal.
6. Ask children to give reasons for standing where they are on the continuum.

2. SNACK ATTACK

Divide the class into groups. Give each group the same set of pre-packaged snack foods and takeaway wrappers or containers (ensure food labels are displayed, include yoghurt, muesli bars, lollies, chips, biscuits, fruit wrap, burgers, fried chips, etc.).
Ask children to select their favourite food from those provided and say why.

Ensure children know how to read the labels.

This indicates the number of servings per package. It is simply a guide given by the food manufacturer.	For consistency compare products per 100g.	This is the amount of kilojoules (energy) a food or drink provides.

Nutrition Information<br>Servings per package: 3<br>Serving size: 150g		
	Quantity per serving	Quantity per 100g
Energy	677kJ	405kJ
Protein	6.1g	4.2g
Fat, total<br>saturated	7.4g<br>4.0g	4.9g<br>2.5g
Carbohydrate<br>sugars	18.8g<br>8.8g	12.6g<br>5.9g
Fibre	1.2g	0.8g
Sodium	450mg	300mg

Give each group a focus, e.g. sugar, fibre, fat, sodium (salt), protein. Ask the groups to place the snack food wrappers onto a continuum indicating the lowest levels to the highest levels of their focus area.

3. WALK, TALK, AND GAWK.

Write the following questions on the board. Children then move around the class viewing the continuum outcomes from the other groups and answering the following questions:

- What did they notice about the continuums?
- How did other continuums compare with your own?
- What conclusions can be drawn about specific snacks?
- Which foods would you consider being a healthy snack and which would be considered a treat. Why?
- What have you found out about your favourite snack?

Thinking about the traffic light system, which category would they give each of these snack foods?
What other healthy snack food and takeaway options can children come up with?

4. JOSTLING FOR POSITION

In groups, children list on separate pieces of paper as many types of takeaway meals that they can think of.
Without looking at any labels, the group try to come to a consensus on the meal they think has the highest content of: salt, fat, carbohydrate, protein, minerals/nutrients, calcium.

Shuffle pieces of paper into an order guessing, which is the healthiest takeaway meal, to that which the group thinks is not such a healthy option. Be prepared to explain why you have positioned each takeaway in its place on the continuum. Were there different points of view amongst your team? How did you come to a final decision? What factors do you think influenced people's thoughts and where you finally placed the takeaway meals?

Have each team member decide which takeaway is their favourite and why. Where on the continuum does it sit? If you applied the traffic lights to this choice, which light would your takeaway most align with? Does this match how often you have this takeaway?

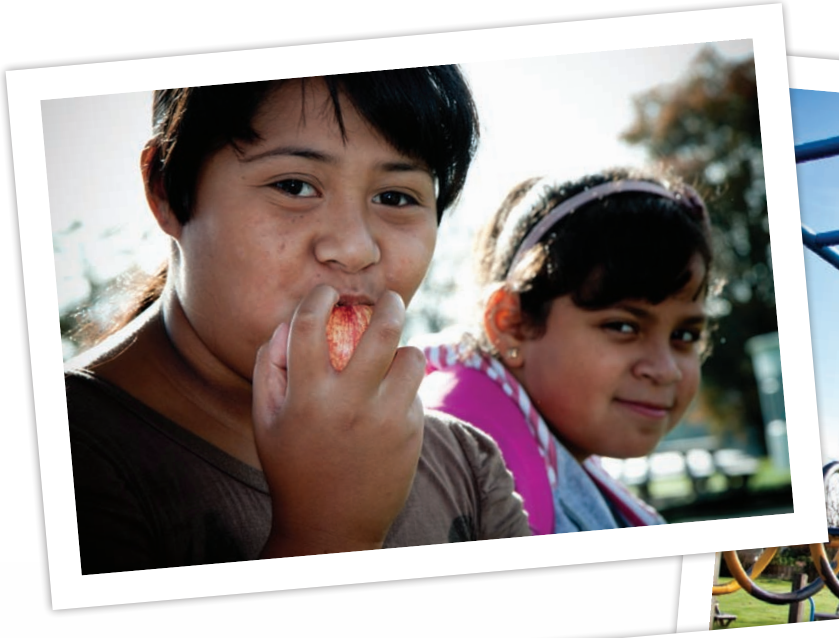

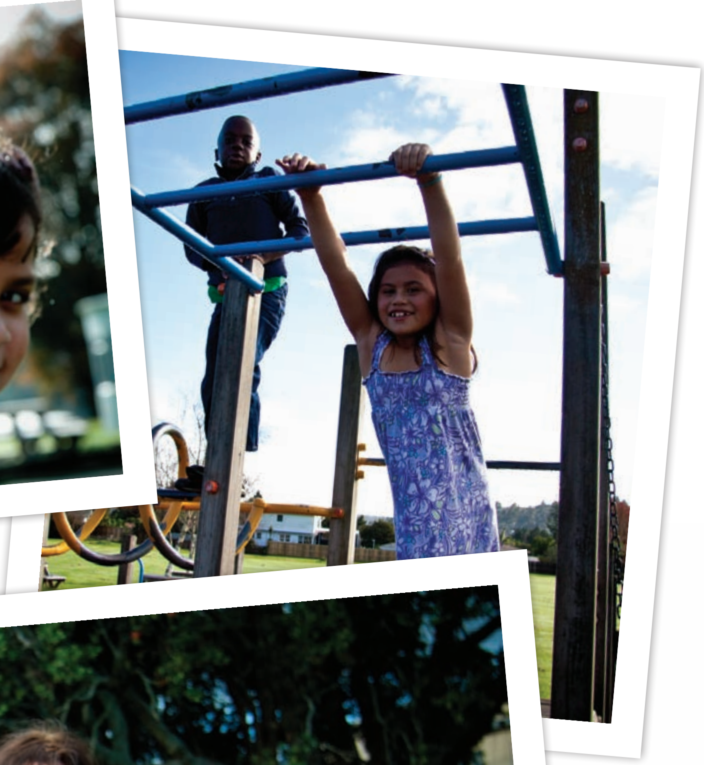

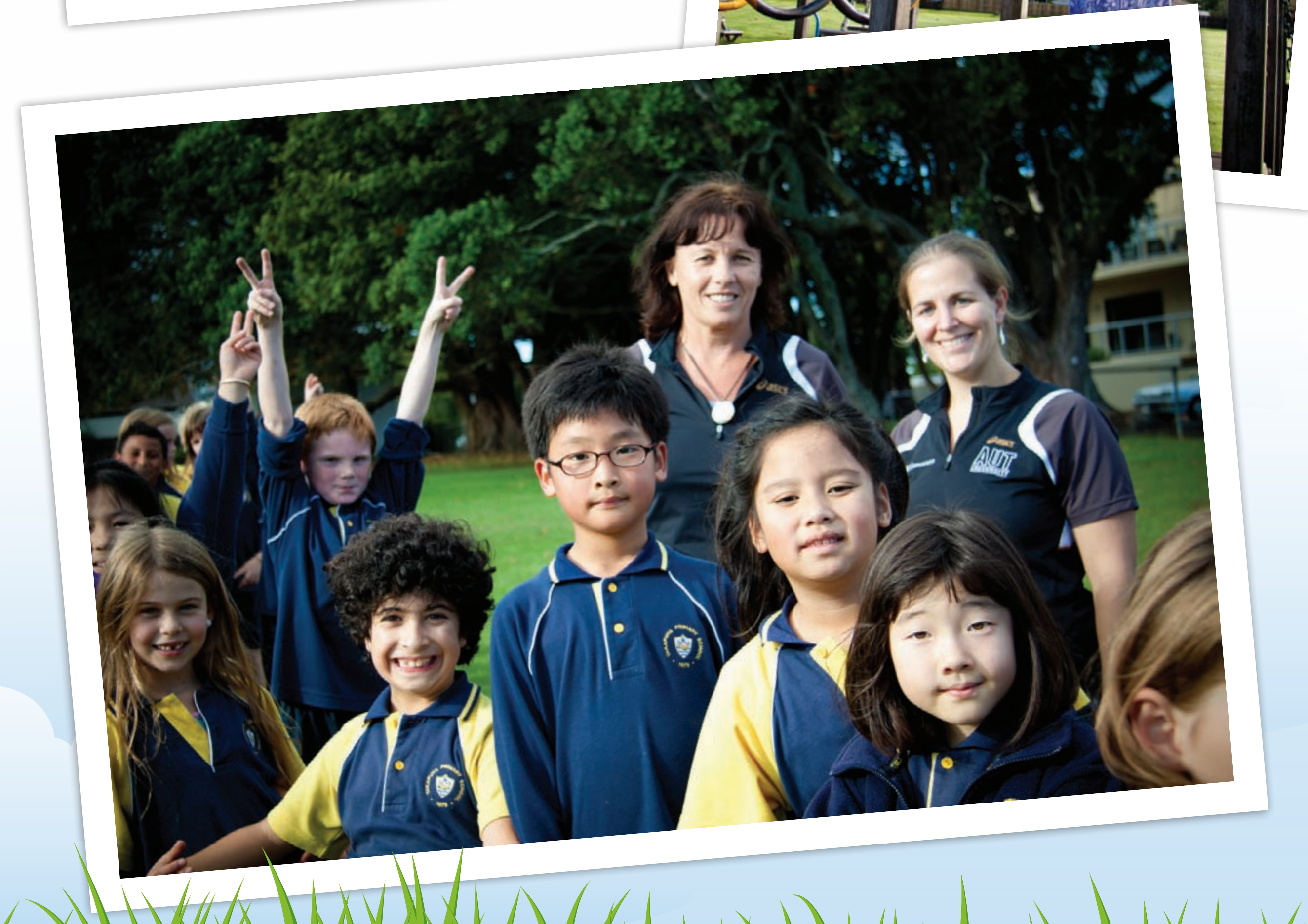

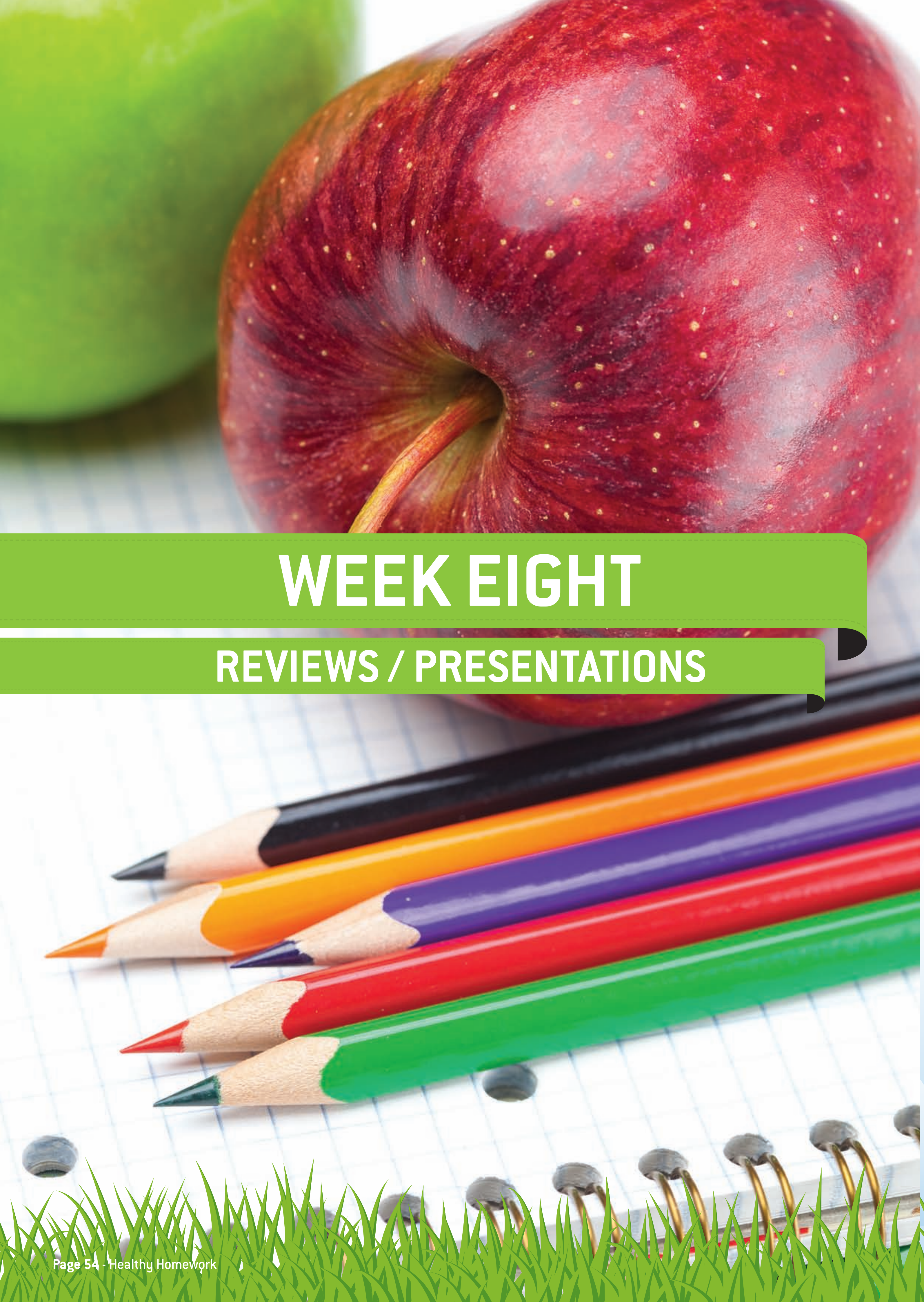

WEEK EIGHT

REVIEWS / PRESENTATIONS

WEEK EIGHT

SESSION 1: HOMEWORK REVIEW

CONFIDENCE AND THE OUTDOORS

Go through all three homework options:

Option A: Facilitate a discussion about the adventure that the children chose to go on with their families. What did they like or dislike about it? Did any of them go through the bush? What did they notice or learn about their local environment? Post or display photos

Option B: In groups discuss the "Treasure Hunt" and if possible share photos. Did they complete all the activities? Were any of the activities difficult to complete? If so, why were they difficult?

Option C: Discuss the reasons why good leadership and communication is important in the outdoors. Discuss what it was like leading someone around an obstacle course when you could touch their shoulders. Was it harder when you could only give verbal directions? What do you need to think about when giving directions to people?

SNACKS AND TAKEAWAYS

Go through all three homework options:

Option A: Ask the children to present their findings. Some children may have the same snacks but have placed them in different categories. Discuss these differences.

Option B: Ask some children to talk to the class about the food they designed. What is it called and where in the supermarket would you find it etc. Ask children to show the diagrams they have prepared of their "invented" product.

Option C: Children create an advertisement to promote their healthy takeaway modification.

HEALTHY HOMEWORK PRESENTATIONS

Children prepare presentations and share learning with the class.
Refer to the beginning of the guide for suggested focus and process.

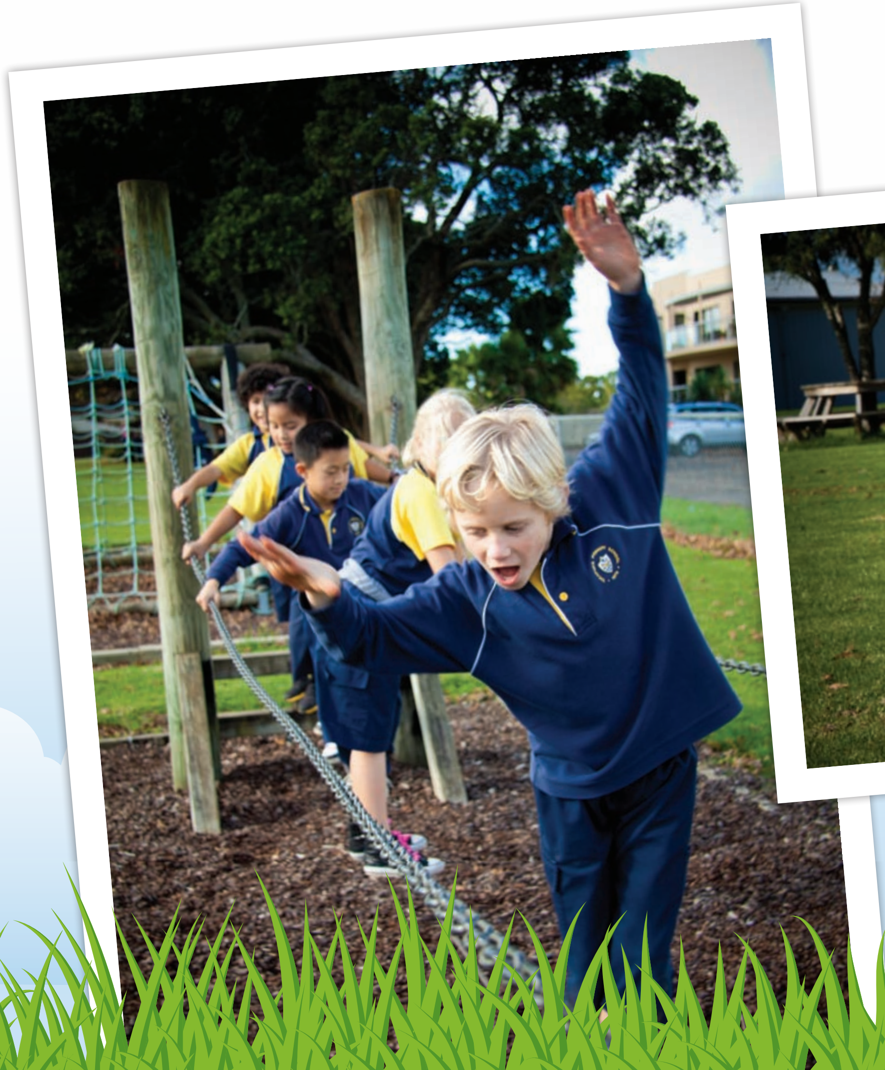

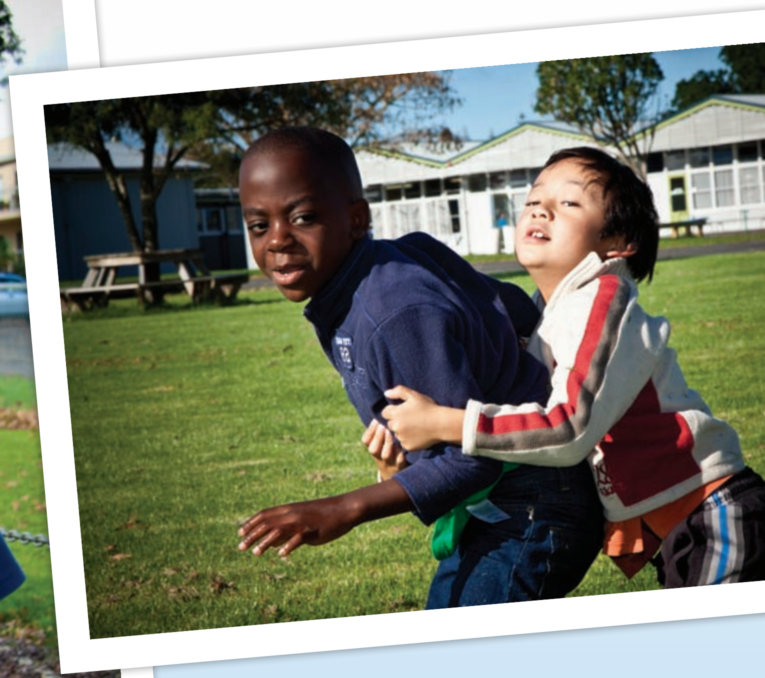

Proudly supported by AUT UNIVERSITY
