# Supplementary material for: Efficacy of a compulsory homework programme for increasing physical activity and improving nutrition in children: a cluster randomised controlled trial
Source: Int J Behav Nutr Phys Act. 2019 Sep 5;16:80. doi: 10.1186/s12966-019-0840-3 (PMC6729097; doi:10.1186/s12966-019-0840-3)
Supplement: Supplementary file 3 — Students Manual. (PDF 10554 kb) [file 12966_2019_840_MOESM3_ESM.pdf]

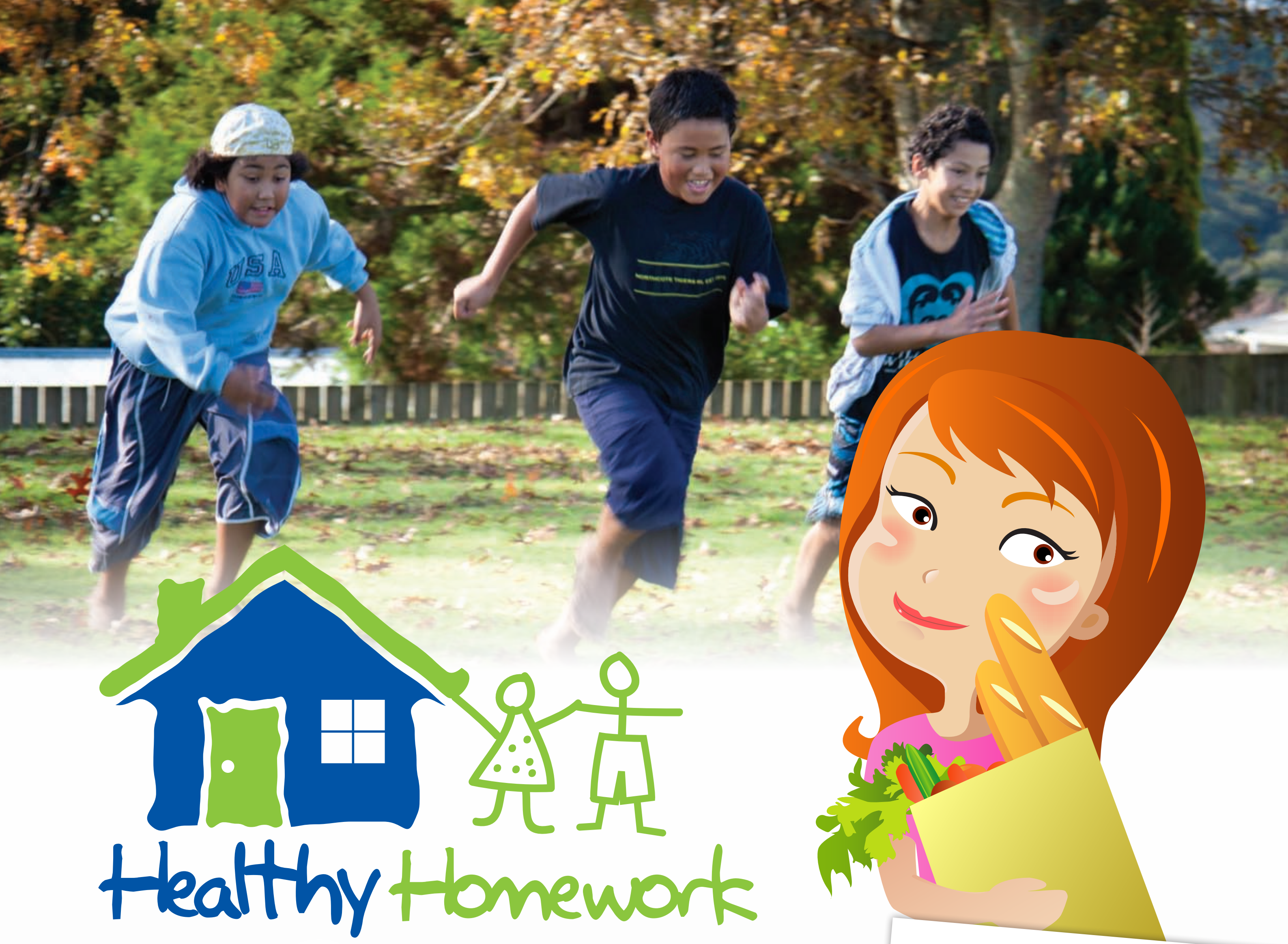

Healthy Homework

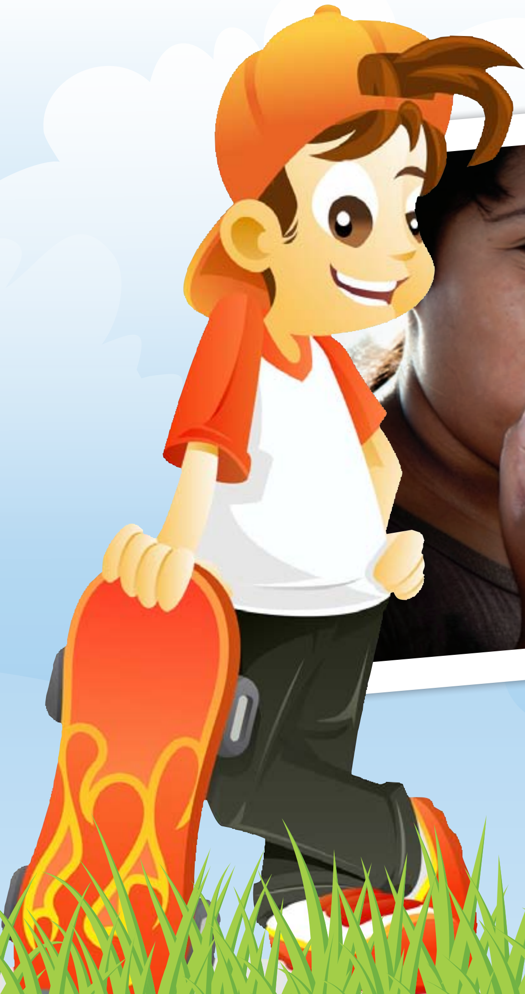

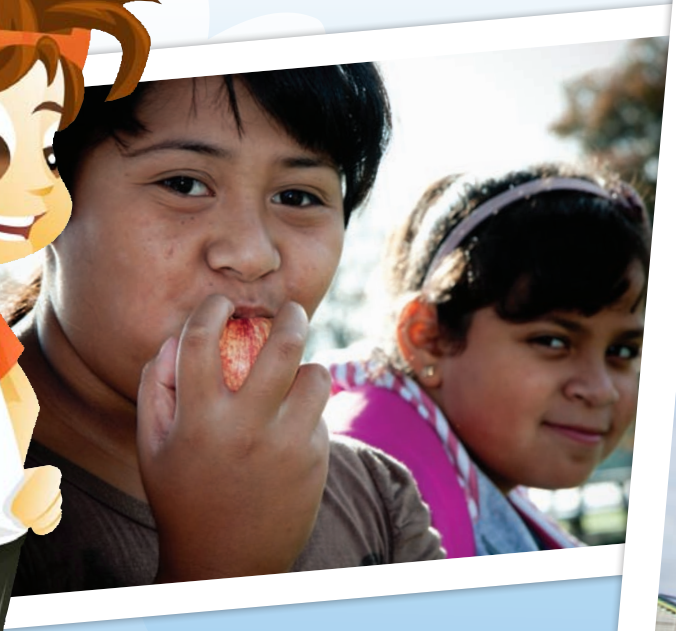

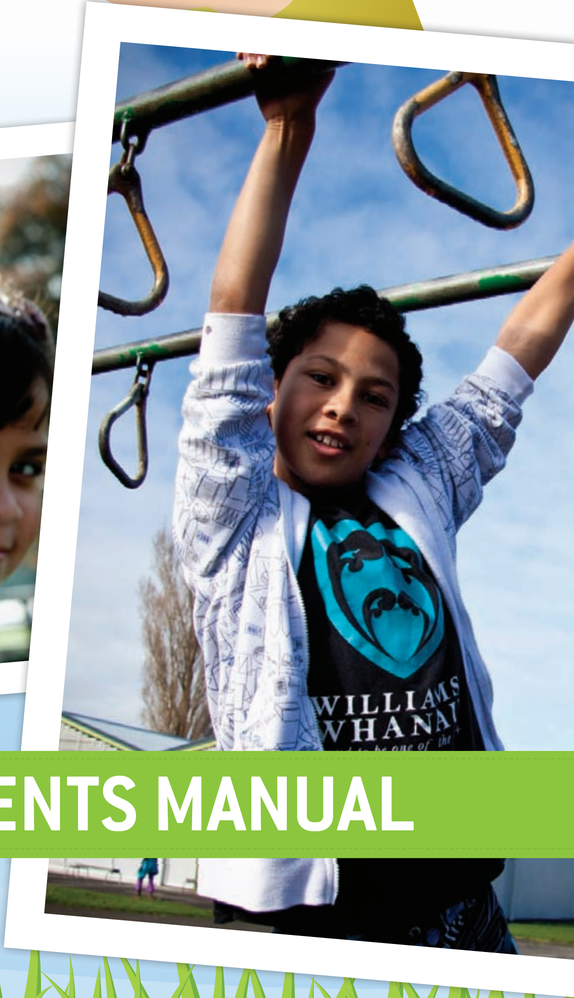

STUDENTS MANUAL

Proudly supported by AUT UNIVERSITY

CONTENTS

WEEK ONE **3**
- Walking 4
- Foods We Eat 6

WEEK TWO **8**
- Screen Time 9
- Energy Foods 10

WEEK THREE **13**
- Sports 14
- General Nutrition 15

WEEK FOUR **18**
- Games 19
- Fruit and Vegetables 20

WEEK FIVE **21**
- Fitness 22
- Drinks 23

WEEK SIX **24**
- Dance & Movement 25
- Breakfast, Lunch and Dinner 26

WEEK SEVEN **28**
- Confidence and the Outdoors 29
- Snacks and Takeaways 30

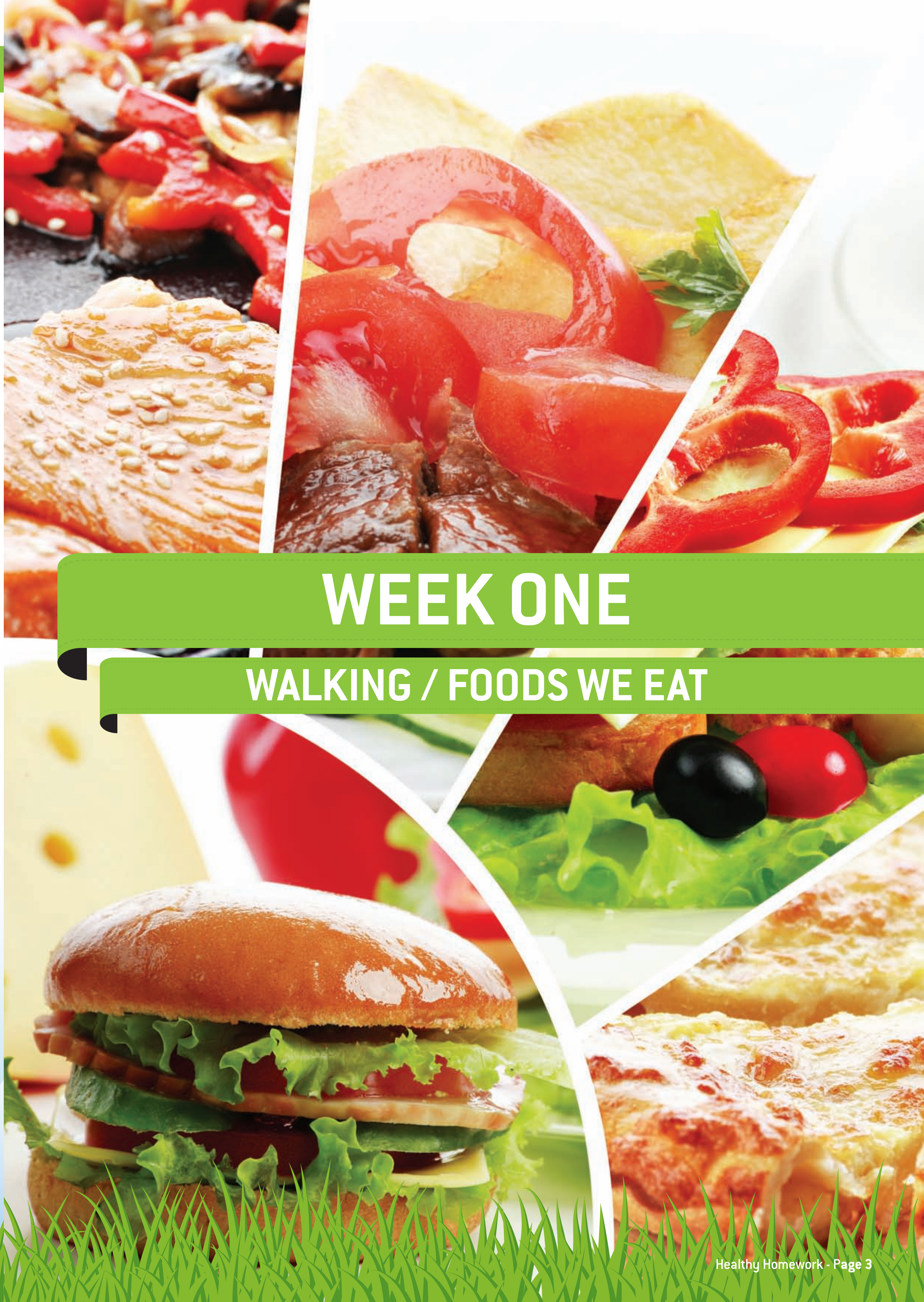

WEEK ONE

WALKING / FOODS WE EAT

WEEK ONE

WALKING

OPTION A

COMPLETED

Task: Go for at least TWO family walks this week, including at least one on the weekend. Even if it is just around the block, anytime you get outside with your family will help you to be healthy and have fun. Perhaps you can walk and talk with a parent, friend, or family member, or take your dog or the neighbour's dog for a walk, or you can try and get all of your family to come with you for a group walk.

Challenge by choice: Try timing how long it takes you to walk a certain route and then beat that time each time you walk it.

Question: How could regular family walks affect your relationship with your family members?

OPTION B

COMPLETED

Task: Talk to an elder within your family or community. Find out about when and where they walked when they were young. Questions could include: How did they get to school? How did they get to the shops? How did they get to places in order to play with their friends? List questions asked and record the person's answers. In the third column answer the questions yourself. What similarities or differences do you notice? Discuss with the older person and record factors that influenced how they got around.

Questions	Older person	Me
How did they get to school?		
How did they get to the shops?		
How did they get to places to play with their friends?		

Question: What are some of the benefits and disadvantages of walking and not walking?

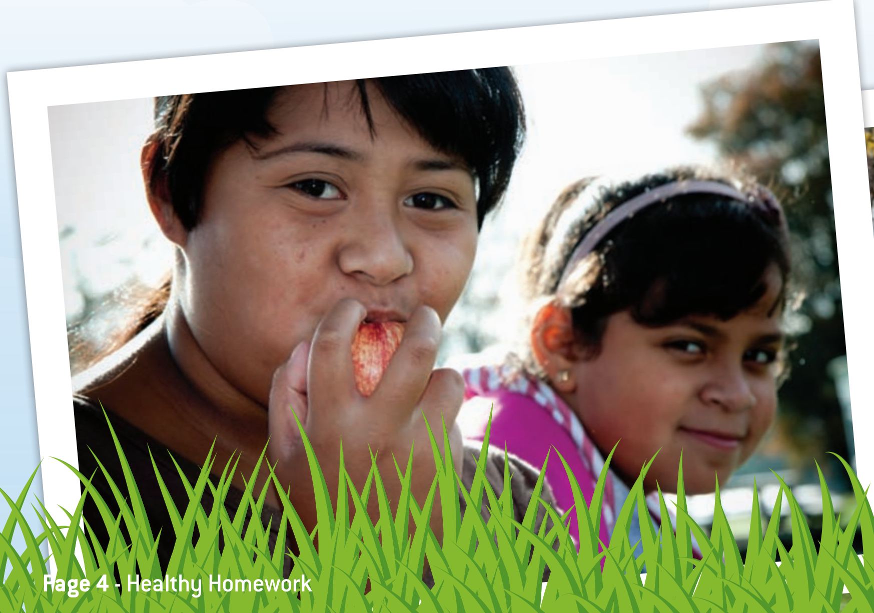

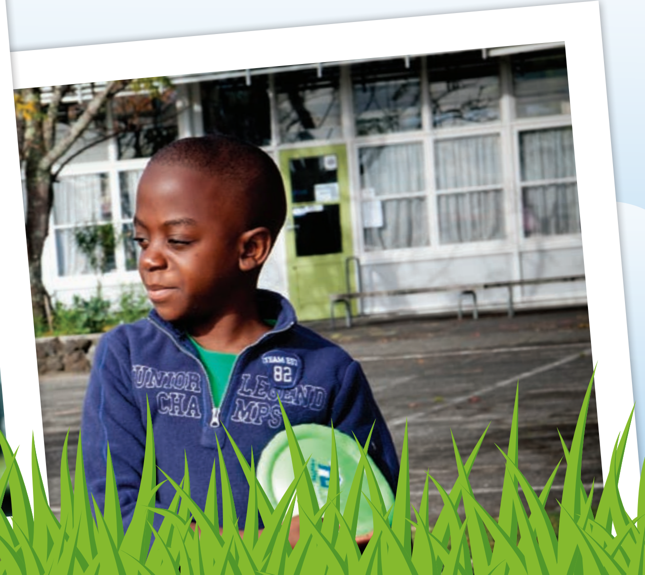

	Benefits	Disadvantages
Walking		
Not Walking		

OPTION C

COMPLETED

Select from one of the following:

Task 1: Walk to or from school at least THREE days this week. If you live too far away to walk, ask the person who looks after you to drop you three or four blocks away from school and walk from there. They could even pick you up from the same place after school. You could organise this with your friends so that you have someone to walk with each day. If you already walk to school, you could create an extra challenge for yourself and run to school. Ask your parents if they could drive the distance for you and tell you how far it is that you walk each day.

Question: What safety issues do you need to be aware of when walking to or from school?

OR if you go to school on a bus, try the following option:

Task 2: Not including getting to or from school, choose at least TWO trips this week that you and your family would normally take the car (e.g. shops, friends' or relatives' house, park, markets) and organise to walk instead. Include at least one on the weekend. If you live too far away from these places to walk all the way, ask the person who looks after you to park three or four blocks away from your destination and walk from there. Ask your parents to measure the distance with the car and record how far you walk each time.

Question: What factors influence your motivation or desire to walk?

I have completed (tick how many) one ◯ two ◯ three ◯ of the options above.

Your signature: ______ Parent signature: ______

REMEMBER to post your photos and blogs on the Healthy Homework website. www.healthyhomework.org.nz

FOODS WE EAT

OPTION A

COMPLETED

Task: Find and collect three recipes of a food dish, each from a different country (use the internet, magazines, old cookbooks, newspaper, etc.).

- List the ingredients of the recipe/food dish.
- Put a mark by the ingredients you are familiar with.
- Which recipe/food dish would you most like to try and why? (If possible help to prepare and serve this dish to your family.)

Question: Which foods were common to more than one recipe/food dish? Make a list.

OPTION B

COMPLETED

Task: Find out which foods are popular and unpopular in your household. Write the person's initials and record answers.

	Food I like	Reasons	Food I dislike	Reasons
MH	Peanut butter	Tastes good on my toast.		

Question: Are they able to say why they like or dislike particular foods? How were their answers similar or different from your own?

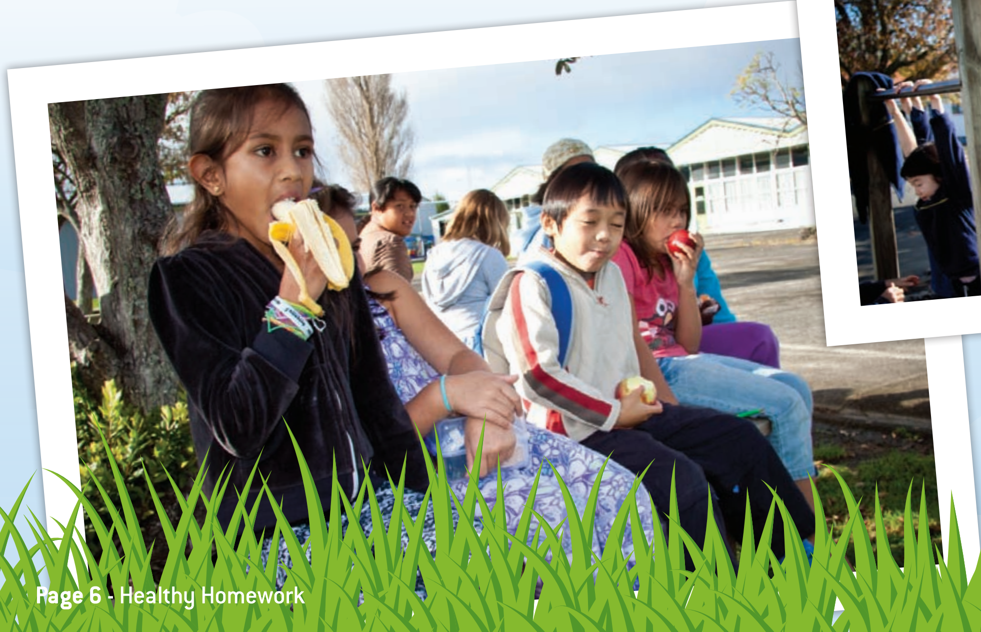

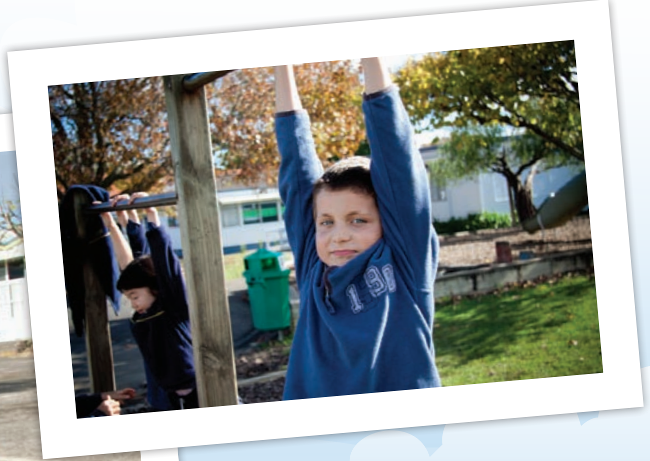

OPTION C

COMPLETED

Task: On the plate outline provided, talk with family members, look in books, or use the Internet to identify 4-5 foods that you can write into each section of the plate.

Find out which nutrients would most likely be found in which food groups.

What food groups were included in your dinner last night?

FOOD PLATE

- Milk, yoghurt, cheese (2-3 servings)
- Vegetables & fruit (At least 3 serves vegetables & 2 serves fruit)
- Food high in fat & sugar (Choose occasionally & in small amounts)
- Lean meat, fish, poultry, eggs, nuts, legumes (1-2 serves)
- Bread, cereals, rice, pasta, noodles (At least 6 serves)

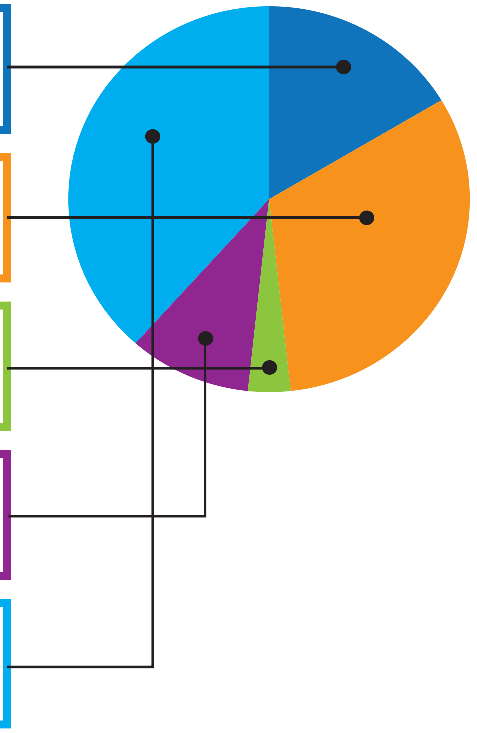

Question: Why do they think the plate has different sized portions for each food group?

I have completed (tick how many) one ◯ two ◯ three ◯ of the options above.

Your signature: ________ Parent signature: ________

REMEMBER to post your photos and blogs on the Healthy Homework website. www.healthyhomework.org.nz

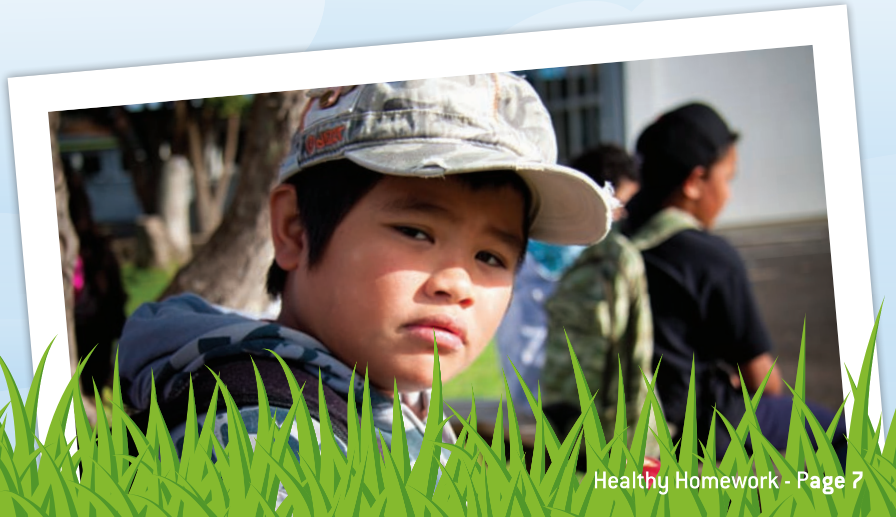

WEEK TWO

SCREEN TIME / ENERGY FOODS

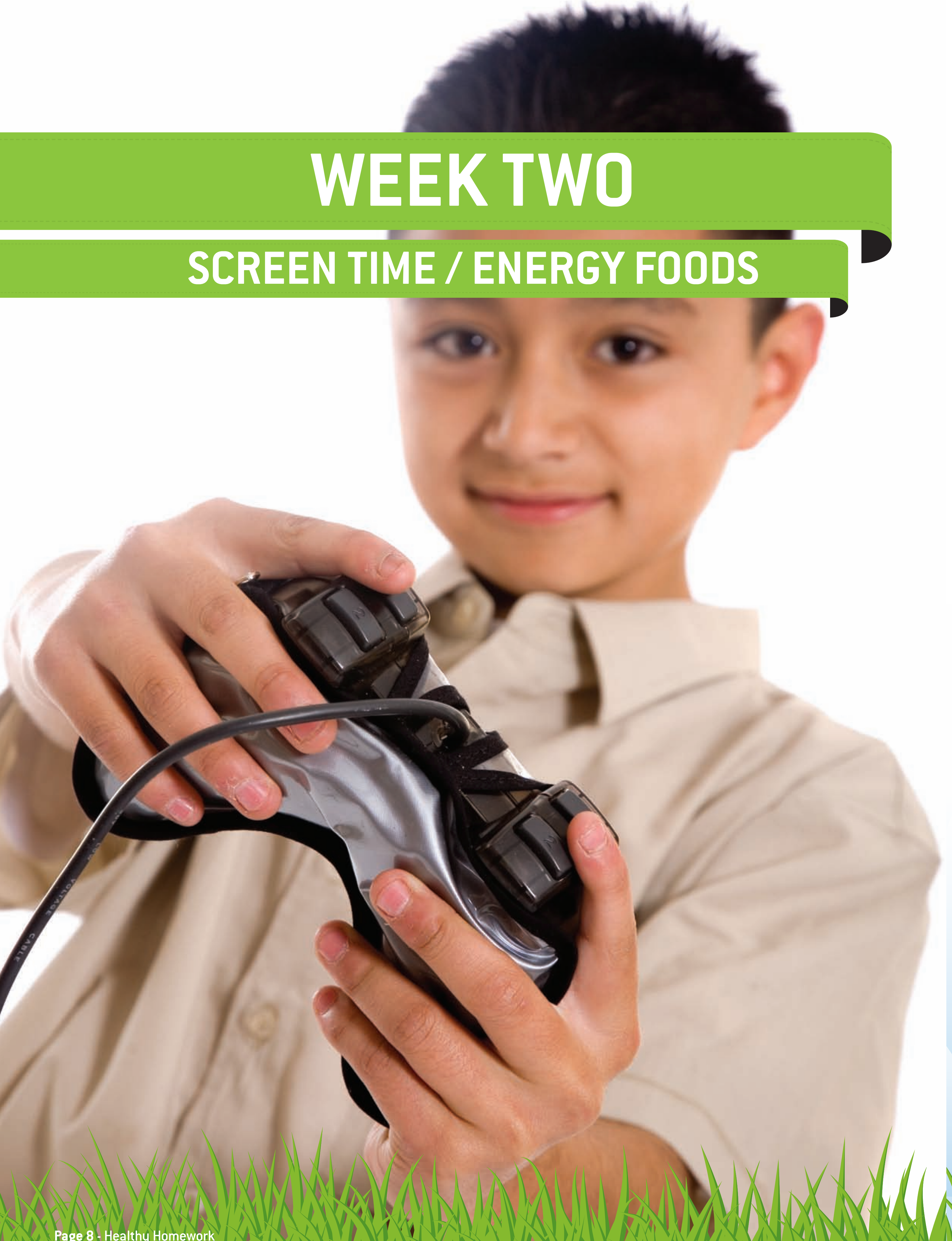

WEEK TWO

SCREEN TIME

OPTION A

COMPLETED

Task: Use ideas discussed in class to make a personal plan to minimise your 'screen time' for at least THREE days this week, (e.g. television, computer games, PlayStation, Xbox, PSP, Wii, etc). Try playing an active game, reading a book, or doing something fun with your family or friends instead. For example, if you watch television while eating your dinner, suggest to your family to turn it off and instead share experiences of the day.

(If the person who looks after you wants you to watch the news on television that's okay – you don't have to count that time).

Questions:

How successful were you?	
What thoughts and feelings did you experience?	
What was easy or difficult about this task?	
What did you try to do to make this happen?	
What did others in your family think about it?	

OPTION B

COMPLETED

Task: For at least THREE days this week, any time you are watching television and an ad break comes on, stand up and try and copy the movements of the people in the ads. If you are watching with others, you can take turns copying the movements or do it all together. If an ad comes on with no people in it, just stand, jump, hop, or walk around the house until it finishes. Make sure you don't sit down until the ad break is over!.

Question: With other family members, can you come up with other suggestions of how to include physical activity into television watching time?

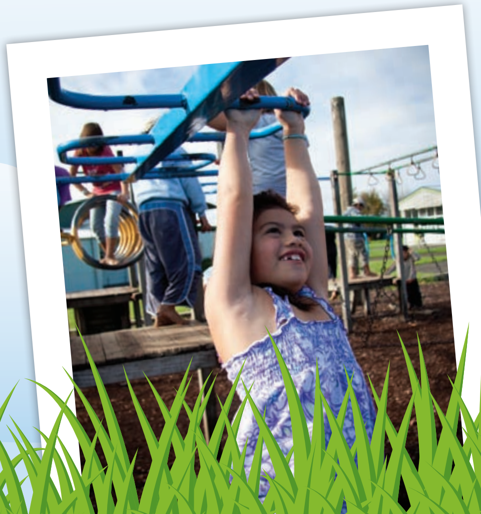

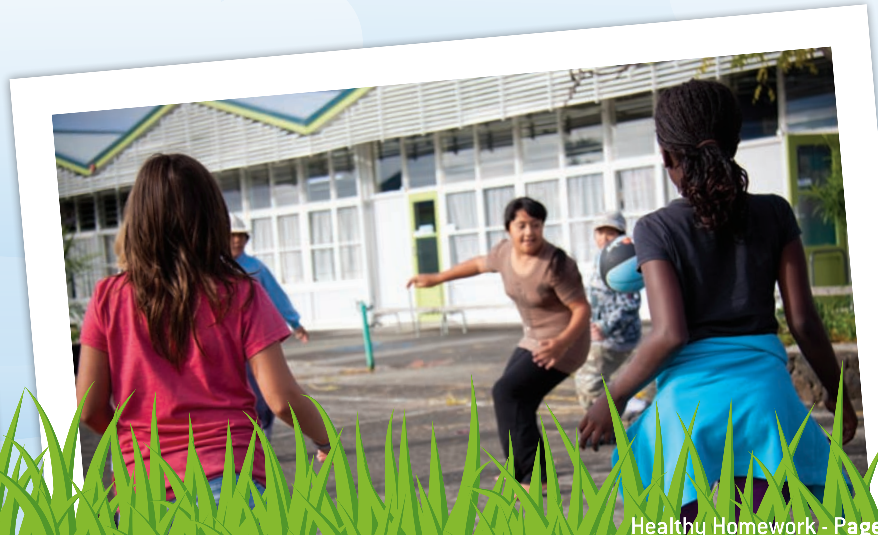

Question : Do you think getting active in the ads could be part of how you watch television in your family?

....

....

....

....

OPTION C

☐ COMPLETED

Task: Use the tally chart below to list the kinds of advertisements that are shown during two television programmes you watch during your allocated television watching time. Indicate the time of day and the programme you were watching.

Name of Programme	Time of Programme	Food	Equipment	Drink	TV Programmes	Travel	Clothes	Other

Question: What have you noticed about the kinds of advertisements shown during each programme?

....

....

....

....

I have completed (tick how many) one ◯ two ◯ three ◯ of the options above.

Your signature: ☐ Parent signature: ☐

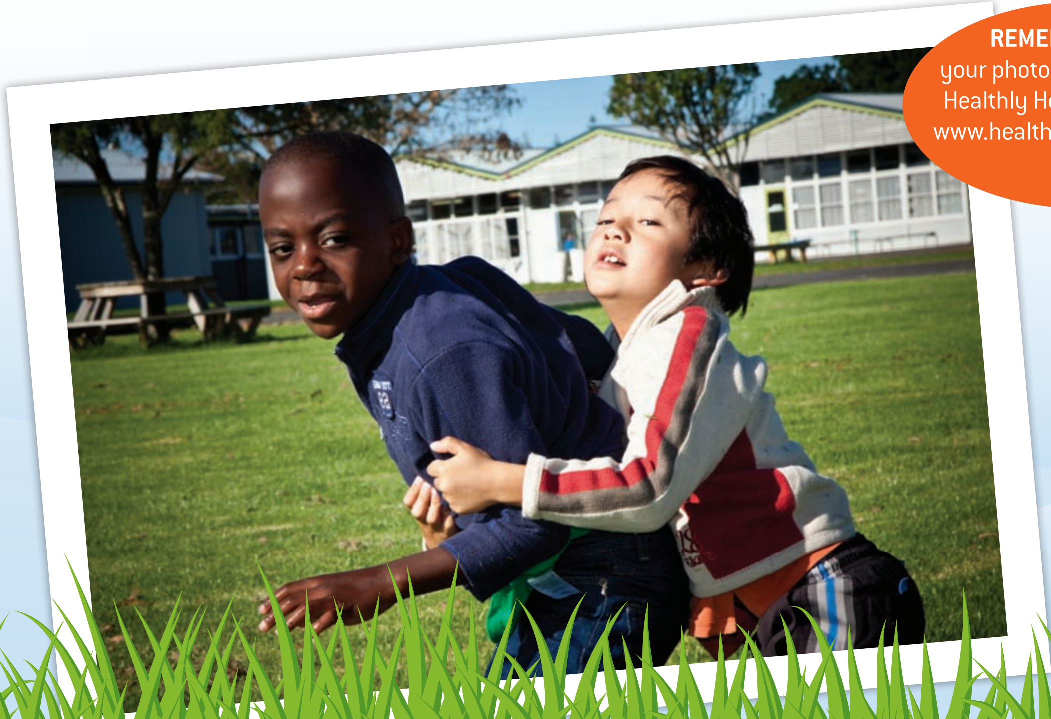

REMEMBER to post your photos and blogs on the Healthly Homework website. www.healthyhomework.org.nz

ENERGY FOODS

OPTION A

☐ COMPLETED

Task: Energy out - Choose three of the activities in the table below and do each of them for five minutes each. When you are doing the activities think about how you are feeling. Do you feel tired, are you puffing or do you have heaps of energy left after the activity?

Kilojoules (energy) used per 5 minutes of exercise	
Cycling	49 kj
Running	98 kj
Football	85 kj
Swimming	73 kj
Walking at a moderate pace	49 kj

Activity 1: ....

On the scale below, circle how much energy you felt like you were using when you did the activity.

1 2 3 4 5 6 7 8 9 10

No energy — Lots of energy

Activity 2: ....

On the scale below, circle how much energy you felt like you were using when you did the activity.

1 2 3 4 5 6 7 8 9 10

No energy — Lots of energy

Activity 3: ....

On the scale below, circle how much energy you felt like you were using when you did the activity.

1 2 3 4 5 6 7 8 9 10

No energy — Lots of energy

Question: How did the amount of energy you used to do the activity relate to the kilojoules that you were using? Were you surprised? Why or why not?

....

....

....

....

....

....

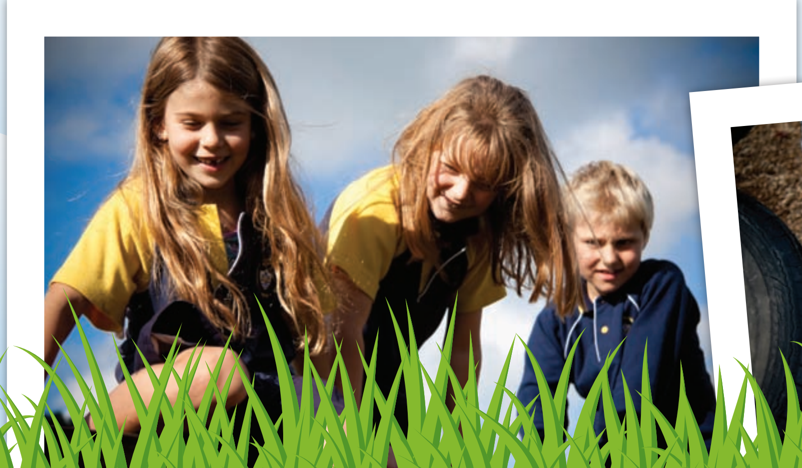

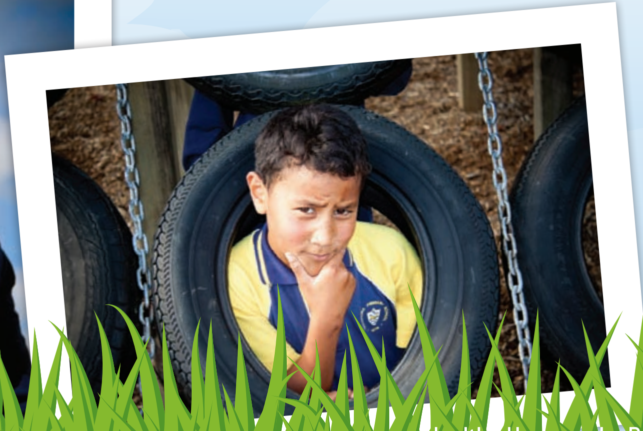

➲ OPTION B

COMPLETED

Task: Energy In - Select a healthy (green) food and an unhealthy (red) food from your fridge, freezer or pantry. Look at how many kilojoules there are in each food per serving size. Using the table in Option A, work out how long you would need to run and walk for to burn the same amount of energy that is in each of the foods.

	Healthy (Green) Food	Unhealthy (Red) Food
Number of kilojoules (kj) per serving		
Number of minutes that you would need to run for		
Number of minutes that you would need to walk for		

Question: What do you think about your results? Did they surprise you?

➲ OPTION C

COMPLETED

Task: Research three ways that people can increase their three non-exercise activity during the day (e.g. walking around when talking on the phone). Create a poster with the three ideas that you have come up with to promote non-exercise activity.

Remember to take you poster to school!

Question: Why is important to do non-exercise activity?

REMEMBER to post your photos and blogs on the Healthly Homework website. www.healthyhomework.org.nz

I have completed (tick how many) one ◯ two ◯ three ◯ of the options above.

Your signature: ______ Parent signature: ______

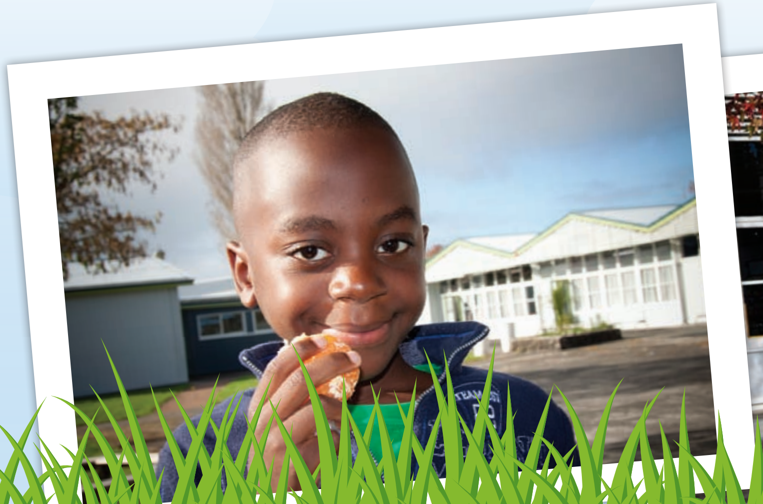

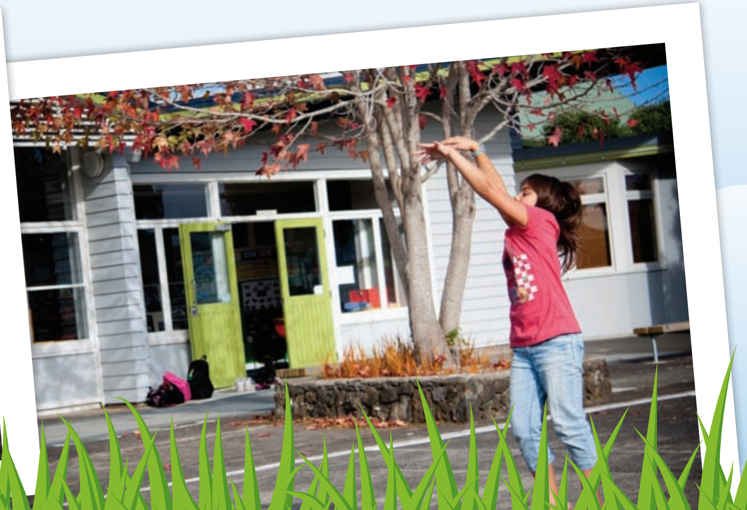

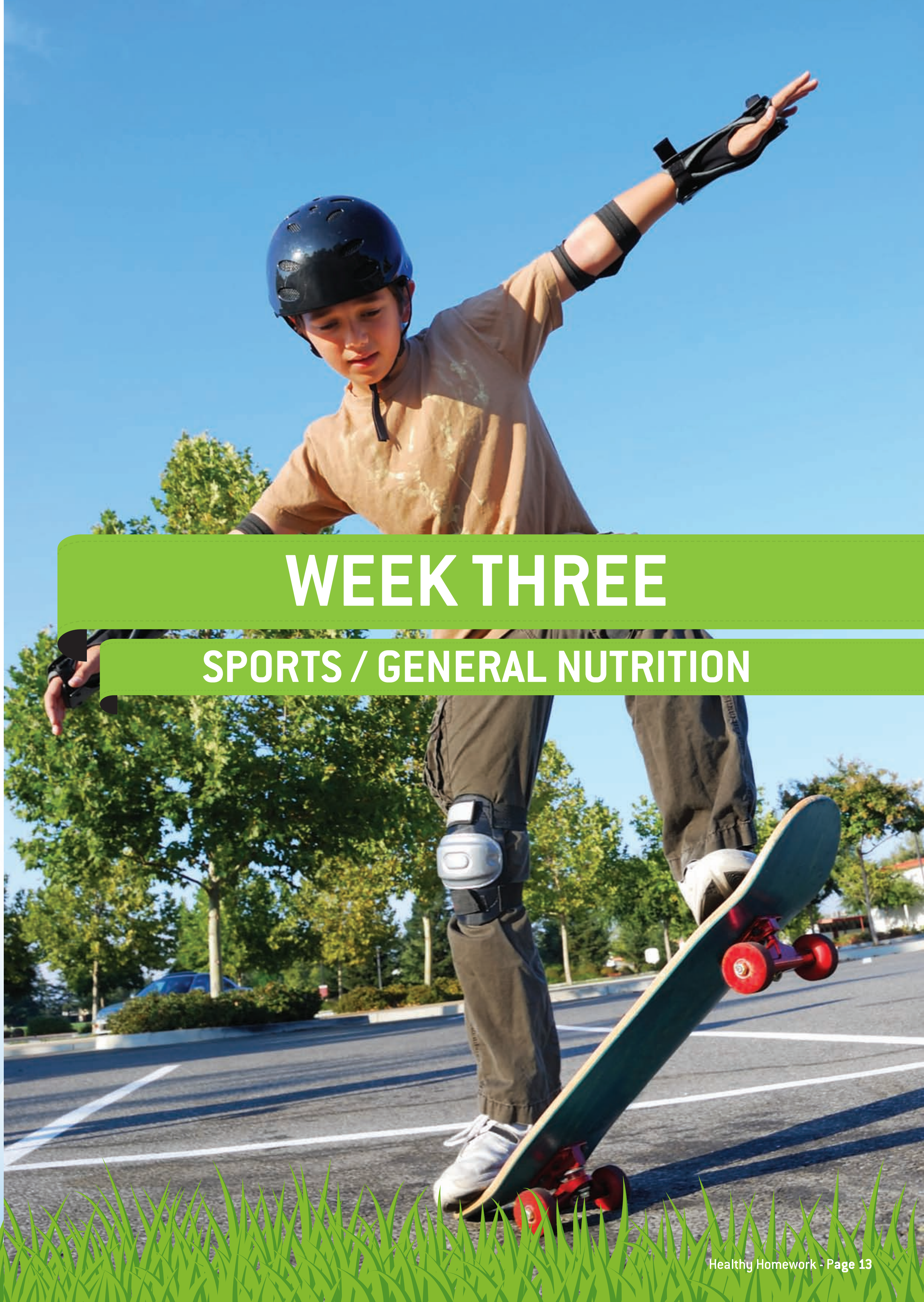

WEEK THREE

SPORTS / GENERAL NUTRITION

WEEK THREE

SPORTS

OPTION A

COMPLETED

Task: Share with your parents or caregivers the legend of ki o rahi and the game. Think about the important skills you needed for this game, go outside and practice one of the important skills. (E.g. dodging, passing to a target, running, etc.) Or practice skills of a sport you enjoy playing. If possible, teach someone a skill from a sport.

Question: Why are practicing skills important for the enjoyment of a game?

OPTION B

COMPLETED

Task 1: Research where some local sports clubs are in your area. Go down to one of your local club grounds (e.g. tennis courts, rugby fields, netball courts, soccer fields, etc.) and play around with a ball with your friends or family. If there is somebody there from the club, you may want to find out some information on joining. Record and describe how belonging to a sports club influences how someone may feel about participating in a sport.

Question: Describe how competition in sport can affect people's behaviour (consider good things and not-so-good things).

OR

COMPLETED

Task 2: Select one of the sports listed in the Culture Counts activity you completed in class or another sport that you are interested in. On your own or with a parent or caregiver, find out two interesting facts about how and/or why this sport was created.

Question: How might a country's culture and environment influence the sports that people of that country play?

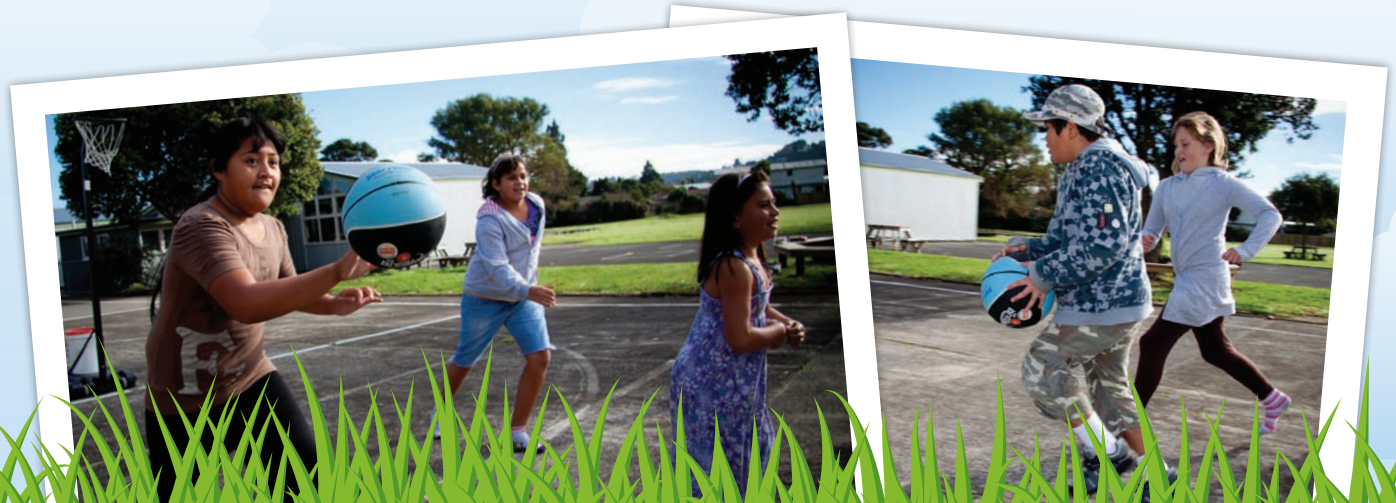

OPTION C

COMPLETED

Task: Ask the person who looks after you to take you to a men's or women's sports game or event this weekend. If this is too difficult, watch a game on television for 15-30 minutes (but don't sit down the whole time!). Spend some time watching how people interact with their teammates and the opposition.

Question: What are the pluses (good), minuses (bad), and interesting things about grown-up sport?

Plus	Minus	Interesting

I have completed (tick how many) one ◯ two ◯ three ◯ of the options above.

Your signature: ______ Parent signature: ______

GENERAL NUTRITION

OPTION A

COMPLETED

Task: Open up your fridge or pantry and select six food items. Write the names of each food/drink on the chart below. Classify each food or fluid into the appropriate traffic light colour.

	Name of item	Red	Amber	Green
Item 1				
Item 2				
Item 3				
Item 4				
Item 5				
Item 6				

Question: Why is a balanced diet important?

REMEMBER to post your photos and blogs on the Healthy Homework website. www.healthyhomework.org.nz

➲ OPTION B

☐ COMPLETED

Task: Using your food diary from the classroom activity, take the list of foods you recorded over the 24-hour period and place them on the food plate below.

FOOD PLATE

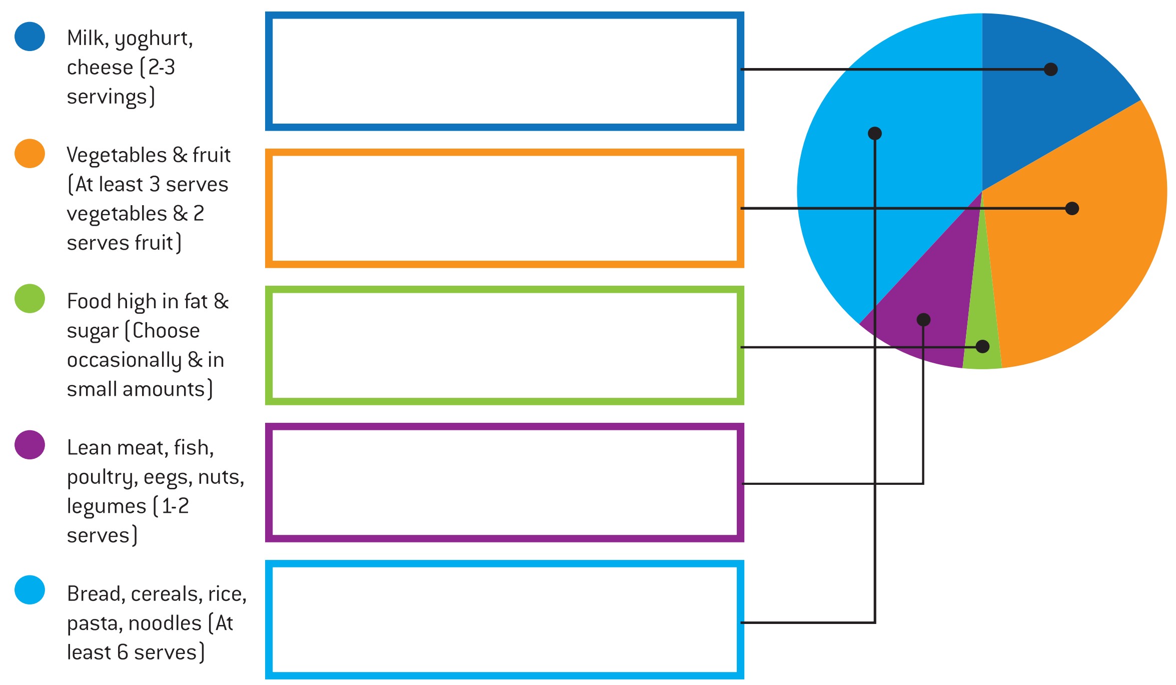

Discuss your chart with a parent /caregiver.
Choose three foods that you have listed in different groups and give a reason why you put it there.

Question: What are you learning about food?

....

....

....

....

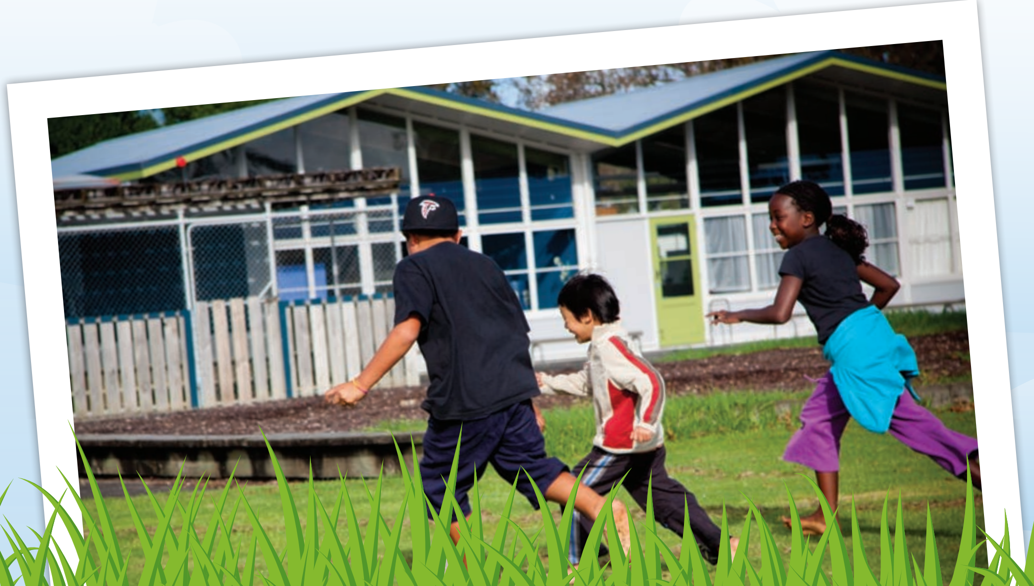

REMEMBER to post your photos and blogs on the Healthy Homework website. www.healthyhomework.org.nz

➲ OPTION C

☐ COMPLETED

Task: With a family member discuss all the different ways you can think that food impacts on your life. You may wish to use the following chart.

Taha tinana – physical<br>E.g. gives me energy to run around.	Taha wairua – spiritual<br>E.g. reminds me of special times.
Taha hinengaro – mental/emotional<br>E.g. it's yummy.	Taha whanau – social<br>E.g. enjoy eating with friends and family.

Question: "We eat food only to keep our body strong". After filling in the above chart what do you think about this statement?

....

....

....

....

I have completed (tick how many) one ◯ two ◯ three ◯ of the options above.

Your signature: ______________ Parent signature: ______________

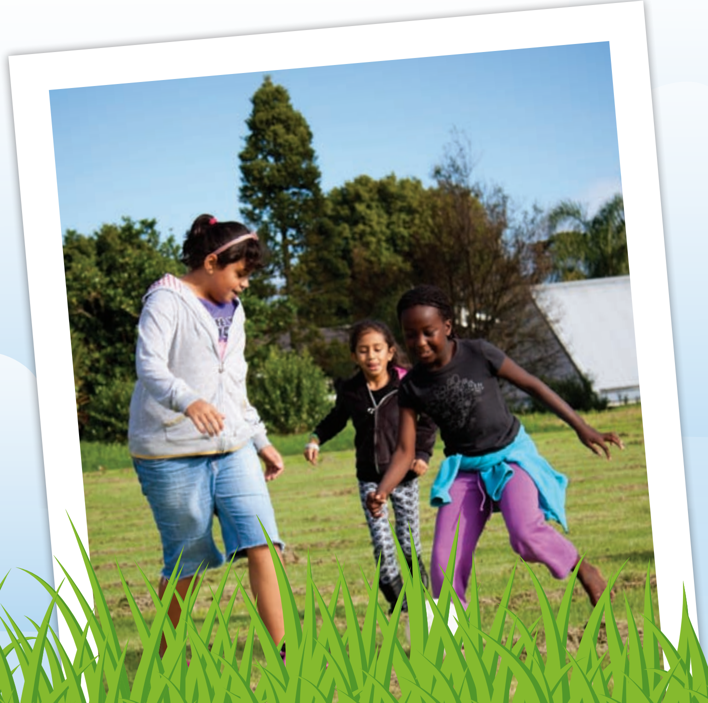

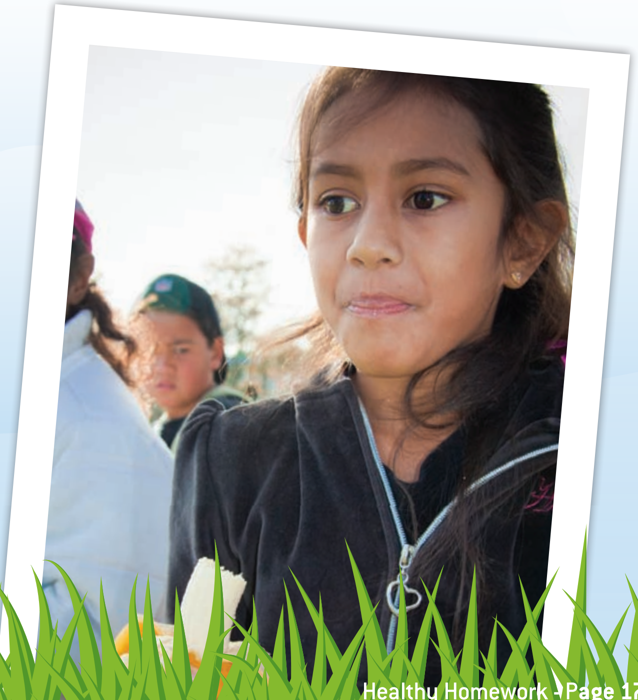

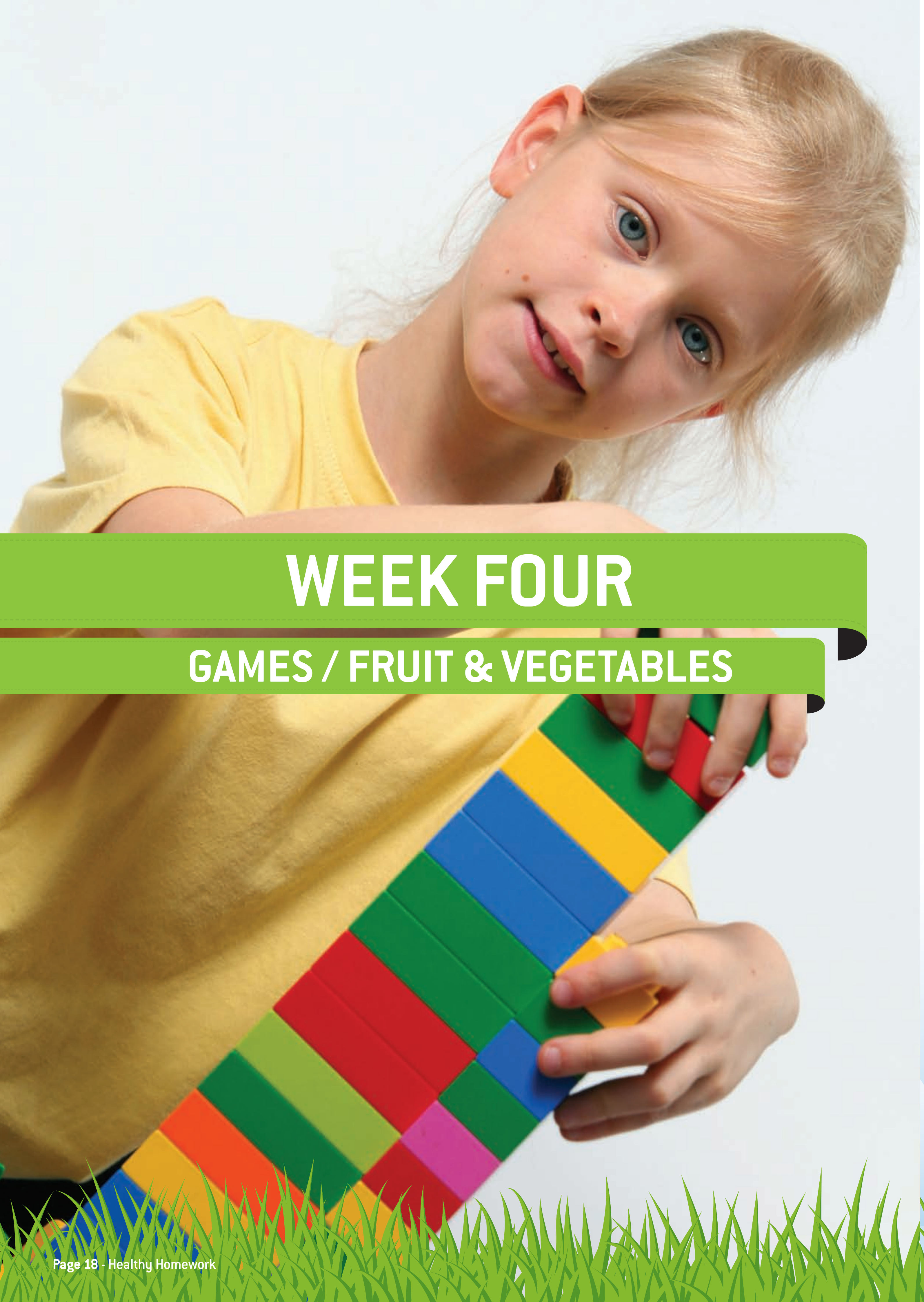

WEEK FOUR

GAMES / FRUIT & VEGETABLES

WEEK FOUR

GAMES

OPTION A

☐ COMPLETED

Task: Invent a game this week that you can play with your friends or your family. Try to use some unusual items that you may have at home, e.g. milk bottles, balls made out of newspaper, plastic containers, etc. Create a poster with the rules for your game.

Question: How does playing a team game affect your relationships with other people?

....

....

....

....

OPTION B

☐ COMPLETED

Task: At least once this week, ask an older person to teach you a game they used to play when they were children. Try playing the game with friends either at school or at home. If you don't know an older person to ask, you may use the Internet or the library to learn a game that people used to play.

Questions: Were the games from other time periods or cultures different from the ones you play today? In what ways were they the same or different?

....

....

....

....

....

....

You may be asked to share this game during the 'Review' session next week. Be prepared to bring any equipment that is not available at school that is needed to play the game.

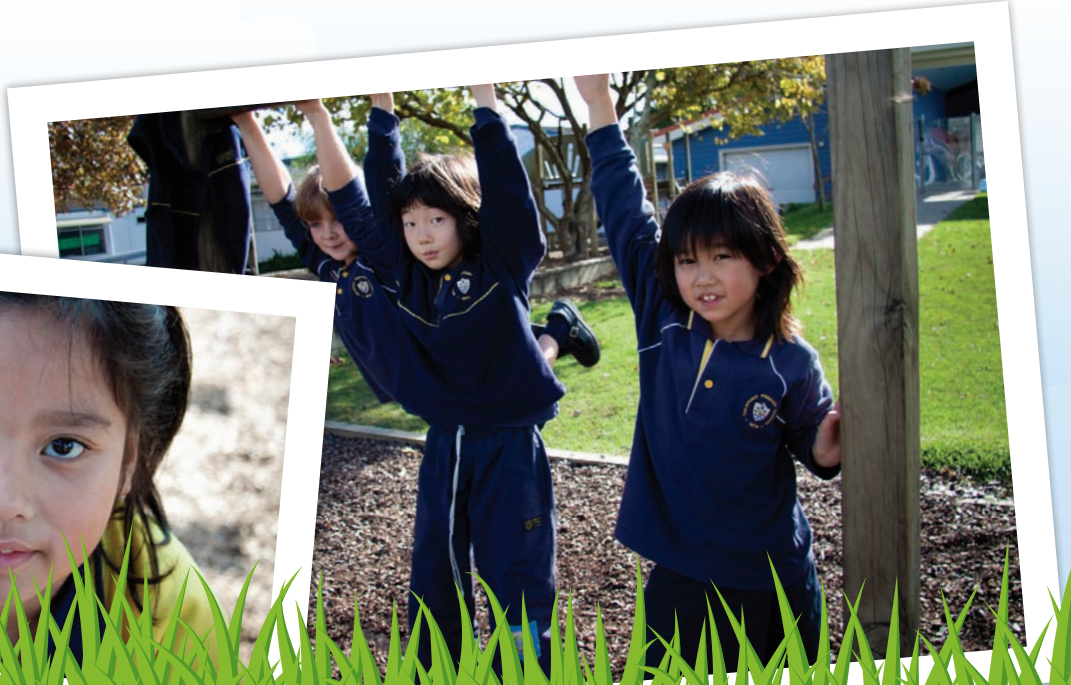

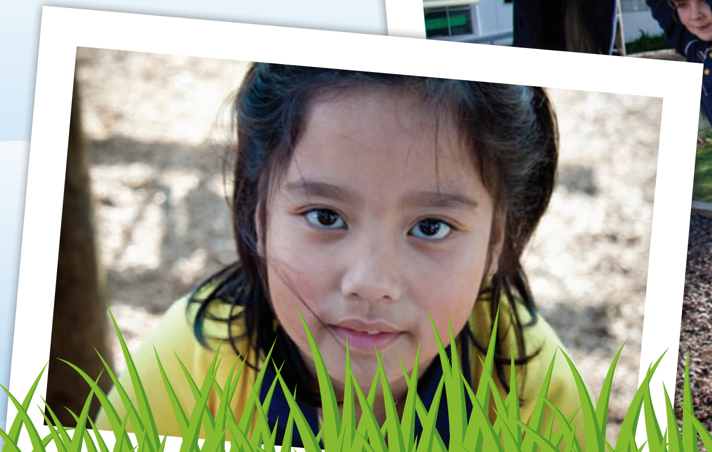

OPTION C

COMPLETED

Task: Spend some time at home inventing and playing a game (or games) that can be played by only one person. If you want you could use a ball or another piece of sports equipment. Once you have made up the rules teach it to a family member.

Question: Do you think it is good to play games by yourself sometimes? Why or why not?

....

....

....

....

....

I have completed (tick how many) one ◯ two ◯ three ◯ of the options above.

Your signature: ______________ Parent signature: ______________

FRUIT AND VEGETABLES

OPTION A

COMPLETED

Task: Discuss the table topic cards created in class over dinner with your family. Write notes of the discussion on the back of the card to bring back to class.

Front of card (to be made at school)	Back of card (to be filled out at home)
What is a root vegetable and how many can you think of?	A vegetable that grows below the ground. Its leaves show above the ground. E.g. carrot, turnip, parsnip, potato, kumara.

Question: What were the most interesting things you learned about food during the discussions with your family.

....

....

....

....

OPTION B

COMPLETED

Task: Your task is to attempt to eat your 5+ a day every day this week. Each day you need to eat at least two servings of fruit and three servings of vegetables. A serving of fruit is the size of the piece that fits into the palm of your hand (e.g. one medium apple or pear or two small kiwifruit or mandarins). A serving of vegetables is ½ cup cooked vegetables (e.g. broccoli or green beans or one cup raw salad). You will need to ask your parents or caregiver to help you count your veggie servings. For your homework, use the table below to tick the box when you meet your fruit and veggie goal (remember that you can include canned or frozen fruit and vegetables).

	Day 1	Day 2	Day 3	Day 4	Day 5	Day 6	Day 7
2 Fruit serves							
3 Veggie serves							

Question: How did you go? What made it easy or difficult? What did you manage consistently? What was it like to be so aware of what you are eating?

....

....

....

....

....

....

....

OPTION C

COMPLETED

Task: Discuss with the person who prepares meals in your house to see if you can eat a dinner meal this week that contains THREE vegetables of different colours. If possible help with preparations and if you can, take a photo of your dinner plate before you eat to post on the Healthy Homework website. You can choose from the following examples of vegetables to get your colours.

If this is difficult, draw or cut pictures from a magazine to create a picture of a meal that has three vegetables of different colours.

1. Green (broccoli / green beans / leeks / cabbage / silver beet / green capsicum / Asian greens – bok choy / lettuce / cucumber / peas)
2. Purple (eggplant / purple fancy lettuce / cabbage)
3. Orange (carrots / pumpkin / butternut / orange capsicum)
4. White (cauliflower / potato / onion)
5. Yellow (yellow capsicum / kumara / corn)
6. Red (red capsicum / tomato / radish / beetroot)

Question: How might eating a variety of different colours of vegetables support our health?

....

....

....

....

I have completed (tick how many) one ◯ two ◯ three ◯ of the options above.

Your signature: ______________ Parent signature: ______________

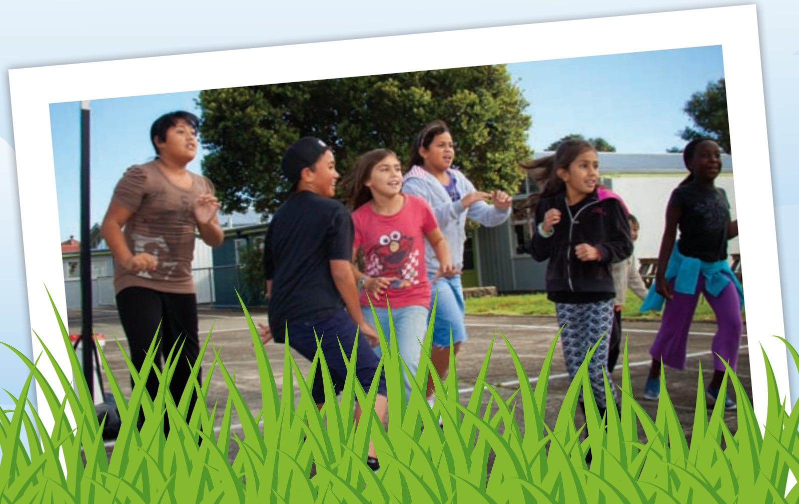

REMEMBER to post your photos and blogs on the Healthy Homework website. www.healthyhomework.org.nz

WEEK FIVE

FITNESS / DRINKS

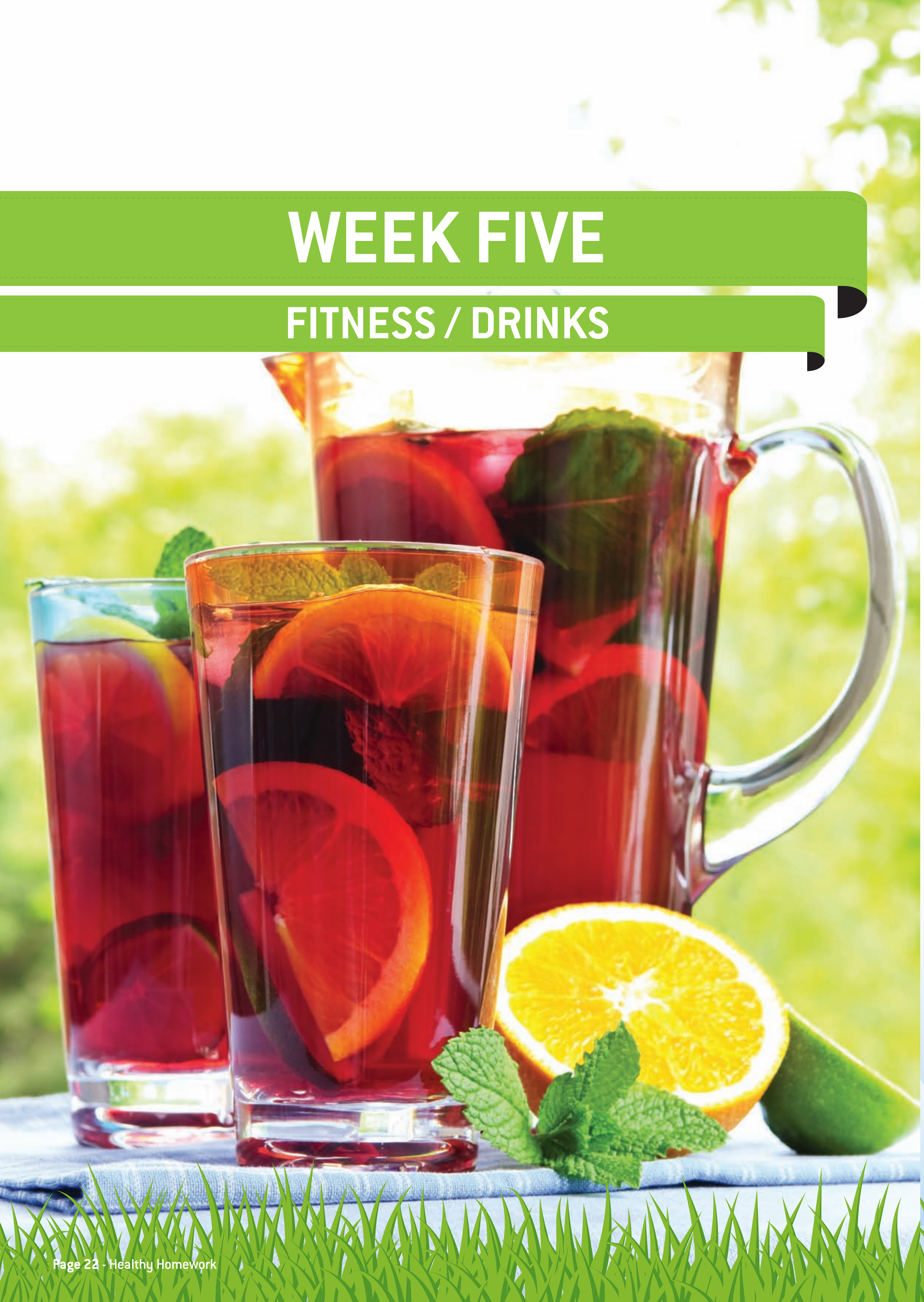

WEEK FIVE

FITNESS

➲ OPTION A

☐ COMPLETED

Task: Decide on an activity you enjoy participating in, e.g. soccer. What is something that you would like to develop, e.g. throw-in from the sideline? What aspects of fitness would help with this skill, e.g. flexibility? Create an activity that would improve this aspect. (It may be a sport you choose or it could be something else like playing on the playground, balancing on different objects, jumping over or across things.)

Question: How will you know you when you have increased your 'fitness' in this area? What will you feel, what will you be able to do?

....

....

....

....

....

....

....

....

➲ OPTION B

☐ COMPLETED

Task: Participate in a running circuit on at least TWO occasions this week, e.g. go for a 10-15 minute run around the school field or create an interesting running course. You could do this at lunchtime, before school, after school, or on the weekend. Run separately or with a friend or family member. Write down how you felt before, during, and after each run. Compare each run with the one before.

Questions: How do you imagine you might feel if you did this every day for a month? What would be the pros and cons of doing so? What would be the point of doing this for a month or more?

....

....

....

....

....

....

....

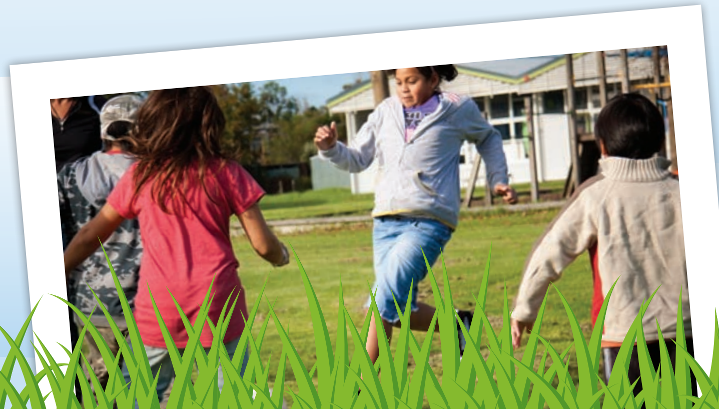

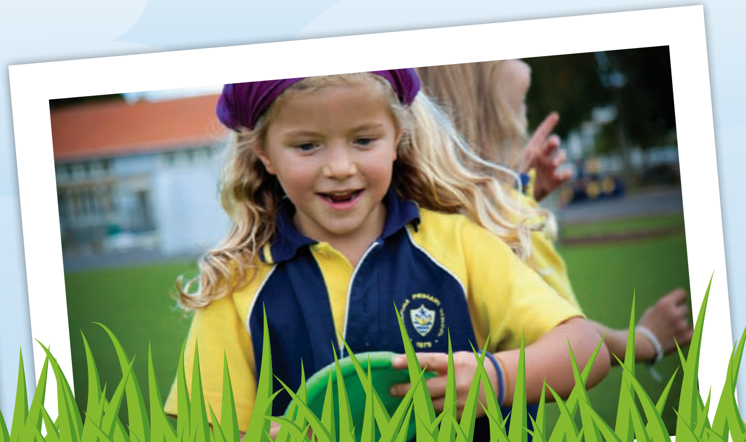

OPTION C

☐ COMPLETED

Task: Create a fitness circuit with 4-5 different exercises. E.g. skipping, hopscotch, shooting and rebounding baskets, dodging through cones or obstacles, etc. Once you have done each exercise, have a short rest and then repeat the circuit 2-3 times. Do the circuit on THREE nights this week. You can use the Healthy Homework website to look for more ideas on exercises to include in your circuit. If possible ask someone to take a photo of you while you are doing your circuit activities and post it on the Healthy Homework website.

Question: What activities were motivating and why? Why might it be useful to try different fitness activities?

I have completed (tick how many) one ◯ two ◯ three ◯ of the options above.

Your signature: ______ Parent signature: ______

DRINKS

OPTION A

☐ COMPLETED

Task: Fill up a drink bottle each morning this week and sip from it throughout the day. If you run out of water, fill it up during the day from the water fountain at school or on the weekend from the kitchen tap. Make sure you get through the whole bottle by the end of each day.

Record how much water you consumed each day?

Monday:	Tuesday:
Wednesday:	Thursday:
Friday:	Saturday:
Sunday:	

Question: What made it easy or difficult to drink consistently? Find out why it may be important not to drink too much water in a day.

OPTION B

☐ COMPLETED

Task: Research energy or sugar-based drinks, list five drinks and five facts about energy and/or fizzy drinks. For at least THREE days this week, replace all energy or fizzy drinks with healthier drinks.

Question: When are these drinks okay to have and what might be the effects if we drink too much of them?

OPTION C

☐ COMPLETED

Task: Interview family members and list all the different types of drinks they have had this week. E.g. water, beer, milk, tea, coffee.

Question: Do you think the fluids you and your family drink often should be healthier? Why or why not?'

I have completed (tick how many) one ◯ two ◯ three ◯ of the options above.

Your signature: ______ Parent signature: ______

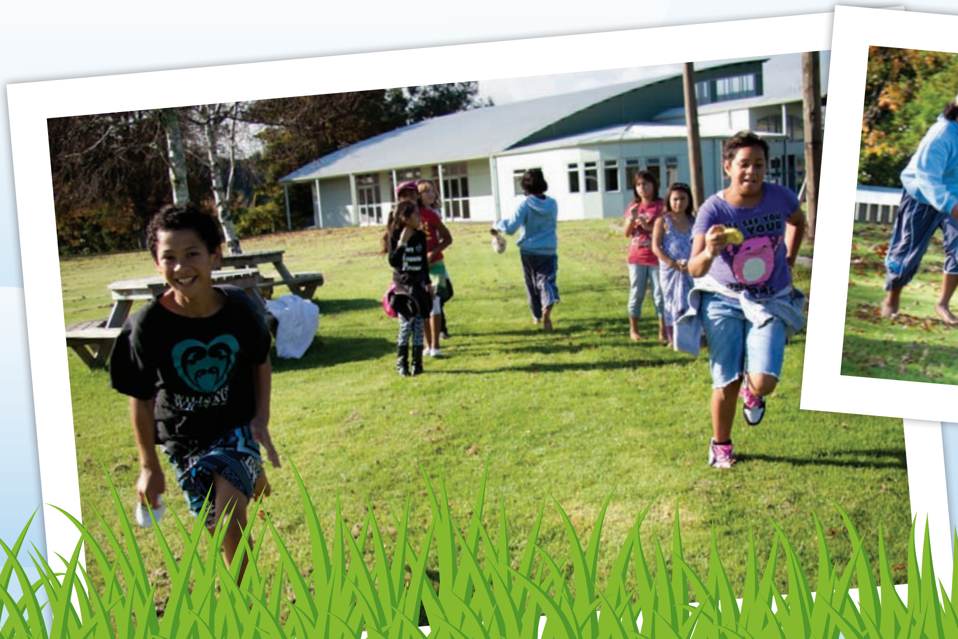

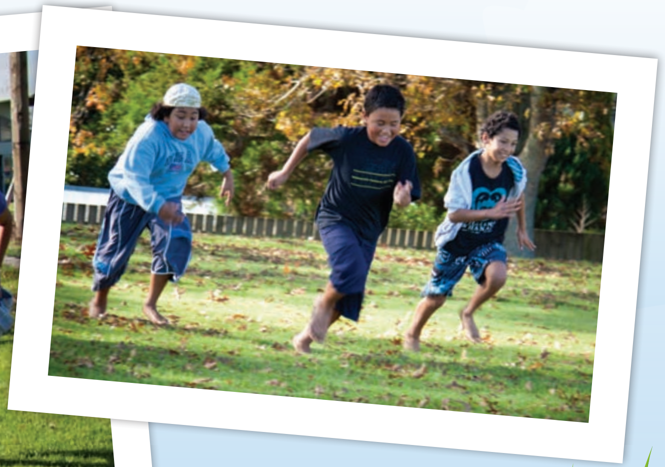

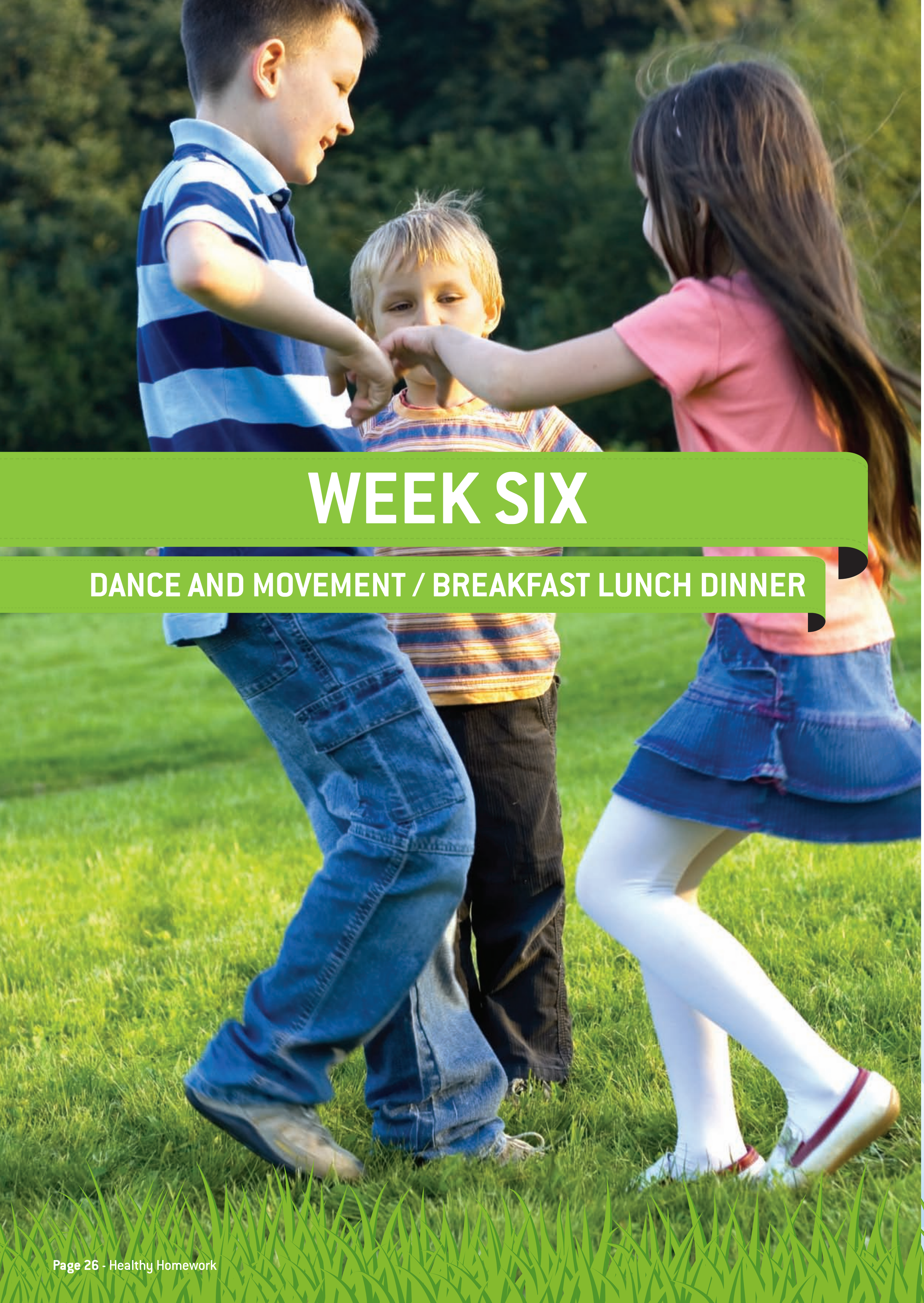

WEEK SIX

DANCE AND MOVEMENT / BREAKFAST LUNCH DINNER

WEEK SIX

DANCE AND MOVEMENT

➲ OPTION A

☐ COMPLETED

Task: Create a simple movement sequence and practice it at least THREE nights this week. Share your moves with your family. If you like, ask someone to take a photo of you while you are performing the dance and post it onto the Healthy Homework website. You can use your own music or the music provided on the Healthy Homework website. If you are stuck for ideas, try telling a story using movement, for example building a house, playing a sport, fishing, or going on an adventure like caving.

Question: What were the challenges you faced in creating and/or sharing this movement sequence? How did you overcome these challenges?

....

....

....

....

....

➲ OPTION B

☐ COMPLETED

Task: Research the traditional dance from the country you chose in class (find pictures, diagrams, or video clips). Attempt to learn at least two movements associated with this style of dance to share with your group back at school. You can use the Healthy Homework website to research the dance moves. If you have a friend or family member who does cultural dancing you can ask them to show you how to perform these dance steps. If you like, ask someone to take a photo of you doing the cultural dance and post it onto the Healthy Homework website.

Question: What country or culture does the dance originate from and what are three special features of the dance?

Country / Culture:

1. ....

2. ....

3. ....

REMEMBER to post your photos and blogs on the Healthy Homework website. www.healthyhomework.org.nz

➲ OPTION C

☐ COMPLETED

Task: On your own or with a friend or family member, flick through radio stations or your own music collection and pretend to be a music star. Use props like tennis rackets or a hairbrush. Sing and move like an artist you like might, or move anyway you like.

Question: In free dancing anything goes. What did you enjoy or not enjoy about this activity?

....

....

....

....

....

I have completed (tick how many) one ◯ two ◯ three ◯ of the options above.

Your signature: ____________________ Parent signature: ____________________

BREAKFAST, LUNCH, AND DINNER

OPTION A

COMPLETED

Task: Plan a lunch menu for the following situations:

- Packed lunch for school
- Cooked lunch at home
- Picnic lunch at the park with your family
- Bought lunch in town

Remember to consider mostly GREEN foods, with less AMBER options, and maybe just 1 RED option in each meal. Also think about who will be with you and consider foods that are appropriate for those people.

School Lunch

Cooked Lunch

Picnic Lunch

Bought Lunch in town

Question: What things did you think about when you were choosing foods for other family members and people of different age groups?

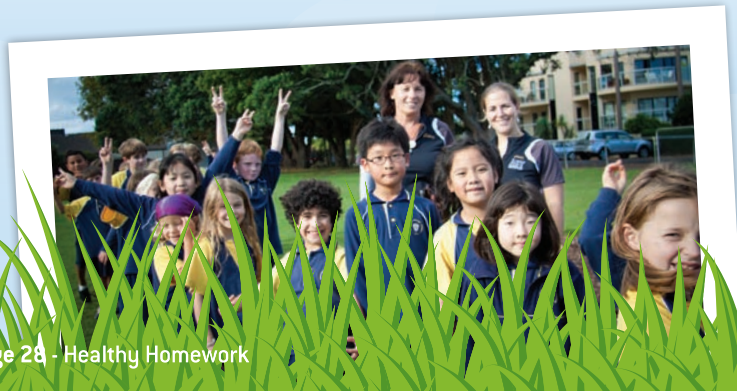

REMEMBER to post your photos and blogs on the Healthly Homework website. www.healthyhomework.org.nz

OPTION B

COMPLETED

Task: Come up with three suggestions of how people could make simple changes to create even healthier breakfasts. Talk about this with other members of your family. E.g. choosing wholegrain bread instead of white bread. Try to make at least one but preferably all three changes at some stage during the week.

Write below the changes you suggest.

	If you already eat:	Change to:
	E.g. white bread	Wholegrain varieties
1		
2		
3		

Question: Did you find that making your breakfast healthier was difficult? How do you think that making these changes all the time might benefit your health?

OPTION C

COMPLETED

Task: This week help a member of your family to cook something. If possible cook something new, i.e. something that you have not cooked before. Examples of new meals to cook can be found on the Feeding our Futures recipe website: www.feedingourfutures.org.nz/recipes.html

If you can, take a photo of your final product and post it on the Healthy Homework website. Remember you get bonus Healthy Homework points by posting blogs and photos. Use your classroom herbs as an addition to your meal.

Question: How could helping in the kitchen affect your relationship with your family members?

I have completed (tick how many) one ◯ two ◯ three ◯ of the options above.

Your signature: Parent signature:

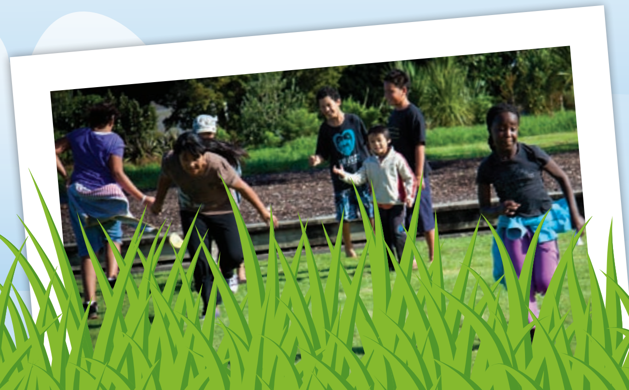

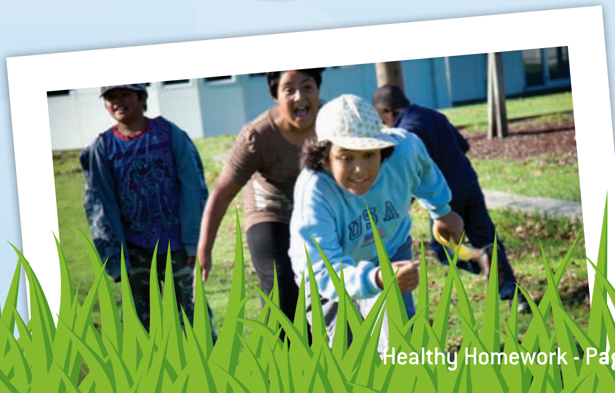

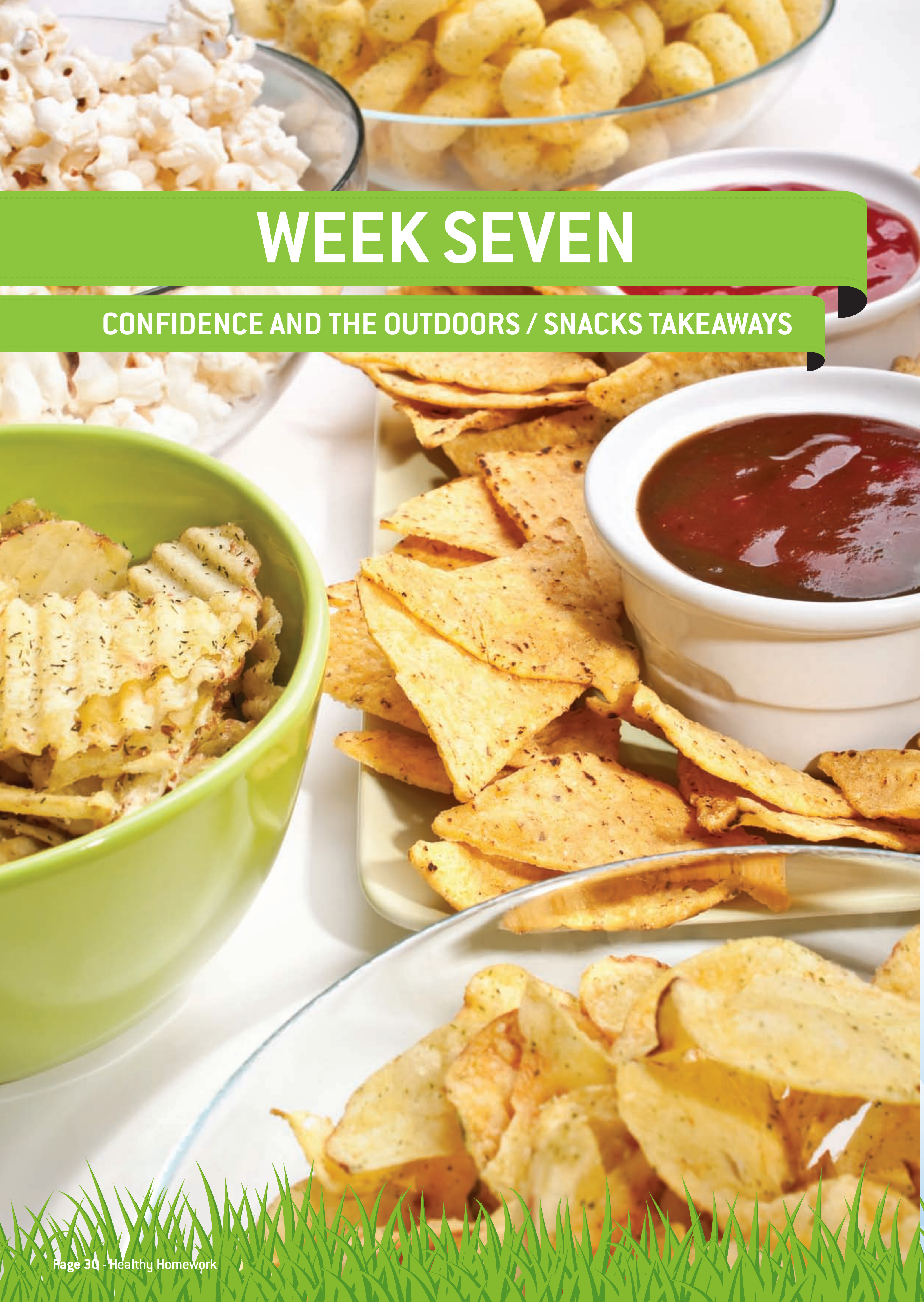

WEEK SEVEN

CONFIDENCE AND THE OUTDOORS / SNACKS TAKEAWAYS

WEEK SEVEN

CONFIDENCE AND THE OUTDOORS

OPTION A

COMPLETED

Task: Plot a tramp on a map of your local area. With a family member or friend, take a small backpack with water, a healthy snack, a warm jersey, and a camera (or phone with a camera feature). See how many of the following features you are able to locate and photograph on your walk: water, an eyesore, something creative, something purple, a reflection, a native tree, an animal, something that is unique to your community, a playground. Mark on the map where your photos are taken. If possible print your photos or sketch a copy of them.

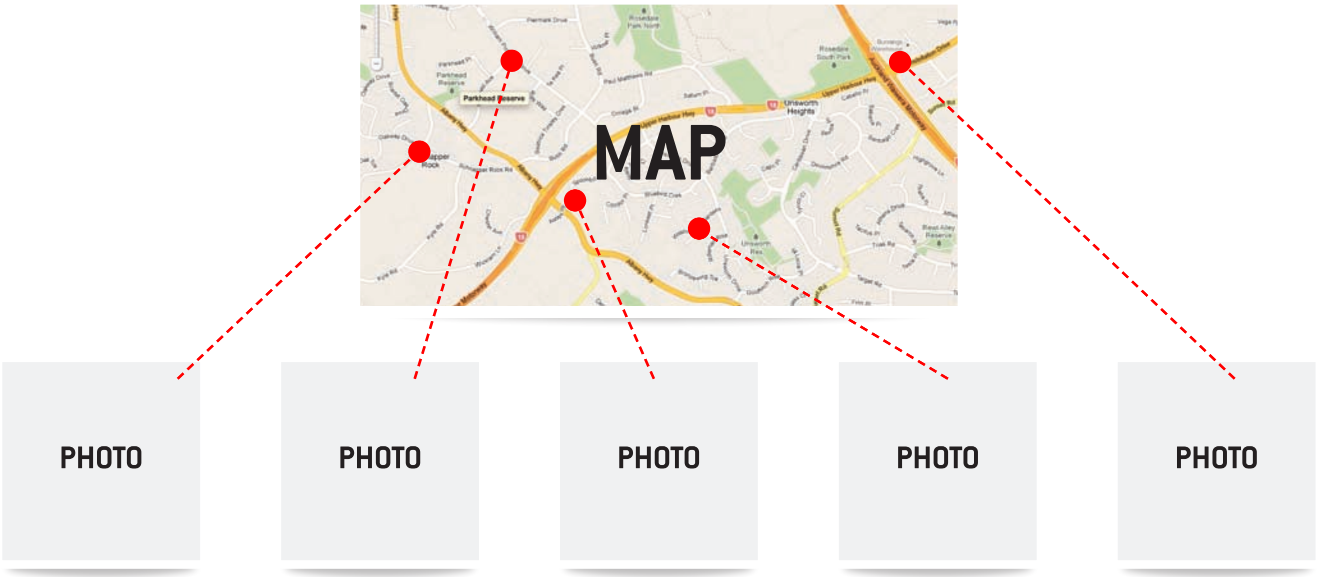

Question: What part of your local area did you feel most proud of as you went on your adventure?

....

....

....

....

OPTION B

COMPLETED

Task: Treasure hunt. Ask your parents if they can take you to a park, beach, or reserve after school one night or at the weekend and find the items listed below. Repeat the treasure hunt (preferably on a different day) and find a different object for each activity listed. You may like to ask someone to take a photo of you doing some of the activities so that you can post them online.

Find something to	Round 1: What did you find?	Round 2: What did you find?
Balance on	E.g. a rock	E.g. a tree stump
Crawl under		
Jump off		
Climb up		
Jump over		

REMEMBER to post your photos and blogs on the Healthy Homework website. www.healthyhomework.org.nz

Question: In what ways do you think that spending time outdoors might be good for you?

OPTION C

COMPLETED

Task: Design an obstacle course that you can lead a family member or friend around. Identify any risks and think about how you will ensure the person's safety. E.g. what will you say and do to keep them safe so they have an enjoyable experience? Ask them to close their eyes. It is your job to direct them around the course.

The first time you lead them around the course you are not allowed to speak but you can guide them by touching their shoulders.

The second time you lead them around the course you can speak to give them directions, but you are not allowed to touch them.

Now, ask your friend or family member to guide you around the course.

Question: Why do you think that good leadership and communication are important in the outdoors?

I have completed (tick how many) one two three of the options above.

Your signature: Parent signature:

SNACKS AND TAKEAWAYS

OPTION A

COMPLETED

Task: Ask your parent or caregiver to take you to a nearby store, supermarket, or dairy (if possible walk).

List in your notebook three snacks from each of the following sections of the supermarket and fill in the chart below.

Fruit and vegetable section	1)<br>2)<br>3)
Confectionary section	1)<br>2)<br>3)
Fridge section	1)<br>2)<br>3)
Biscuit/snack section	1)<br>2)<br>3)

Using the snack food guidelines, list or draw the foods you have chosen under the red, amber, and green foods sections.

Red	
Amber	
Green	

OPTION B

COMPLETED

Task: Fruit makes a really great snack for the lunchbox but sometimes it can get bruised or squashed in your lunchbox. You have a new job as a "fruit designer". Design a new fruit-based snack that could be packaged and sold in the supermarket. Do you have a catchy name for your new product? What is it made of? Draw a diagram of your product and take it to school to present to your class at the homework review next week. If possible take a photo and post it on the Healthy Homework website.

Question: What do you think is the difference between a "snack" and a "treat"?

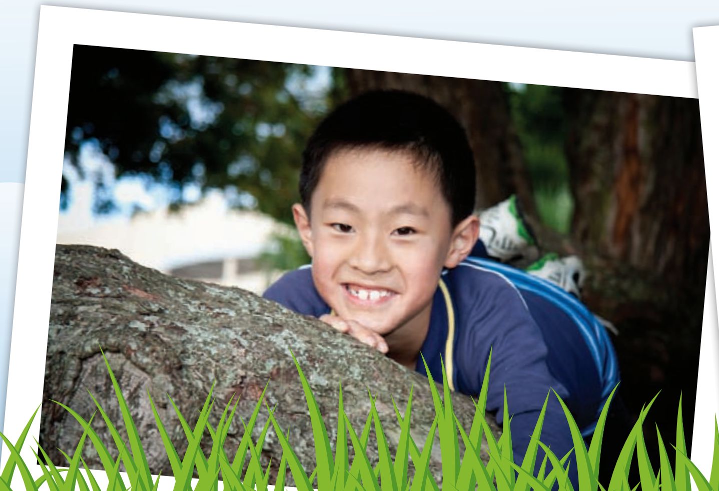

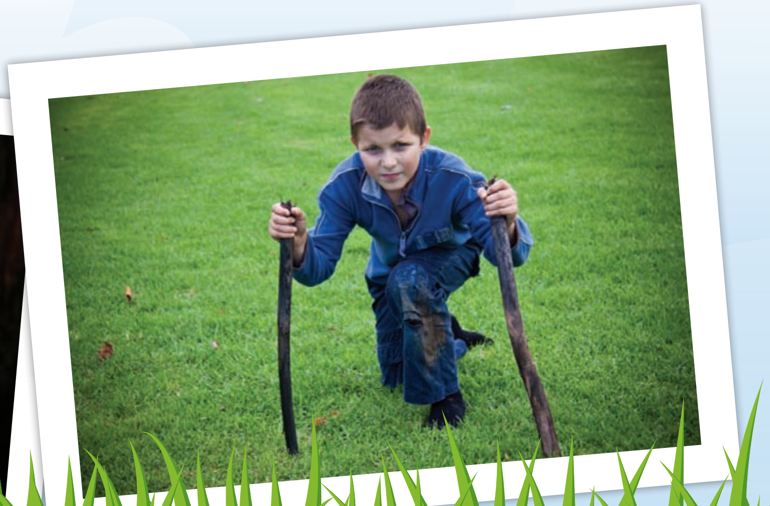

OPTION C

☐ COMPLETED

Task: Vegetables are a great addition to homemade "fast food". Make your own pizza, burger, or wrap for dinner one night this week and include at least three different vegetables. For example, you could make a burger with a slice of tomato, piece of lettuce, and some grated carrot. How about a pizza with thin slices of courgette, onion, and red capsicum? Write down what you made. At this stage your herbs should be ready and should make a tasty addition to your homemade meal.

If it is difficult to make dinner, perhaps you can either draw the meal you would like to make including the different vegetables OR find a picture of a takeaway meal and decide what things you would take out or what things you would put in to make it tasty and nutritious.

Question: What different vegetables could you use next time you make this meal?

I have completed (tick how many) one ◯ two ◯ three ◯ of the options above.

Your signature: ____________ Parent signature: ____________

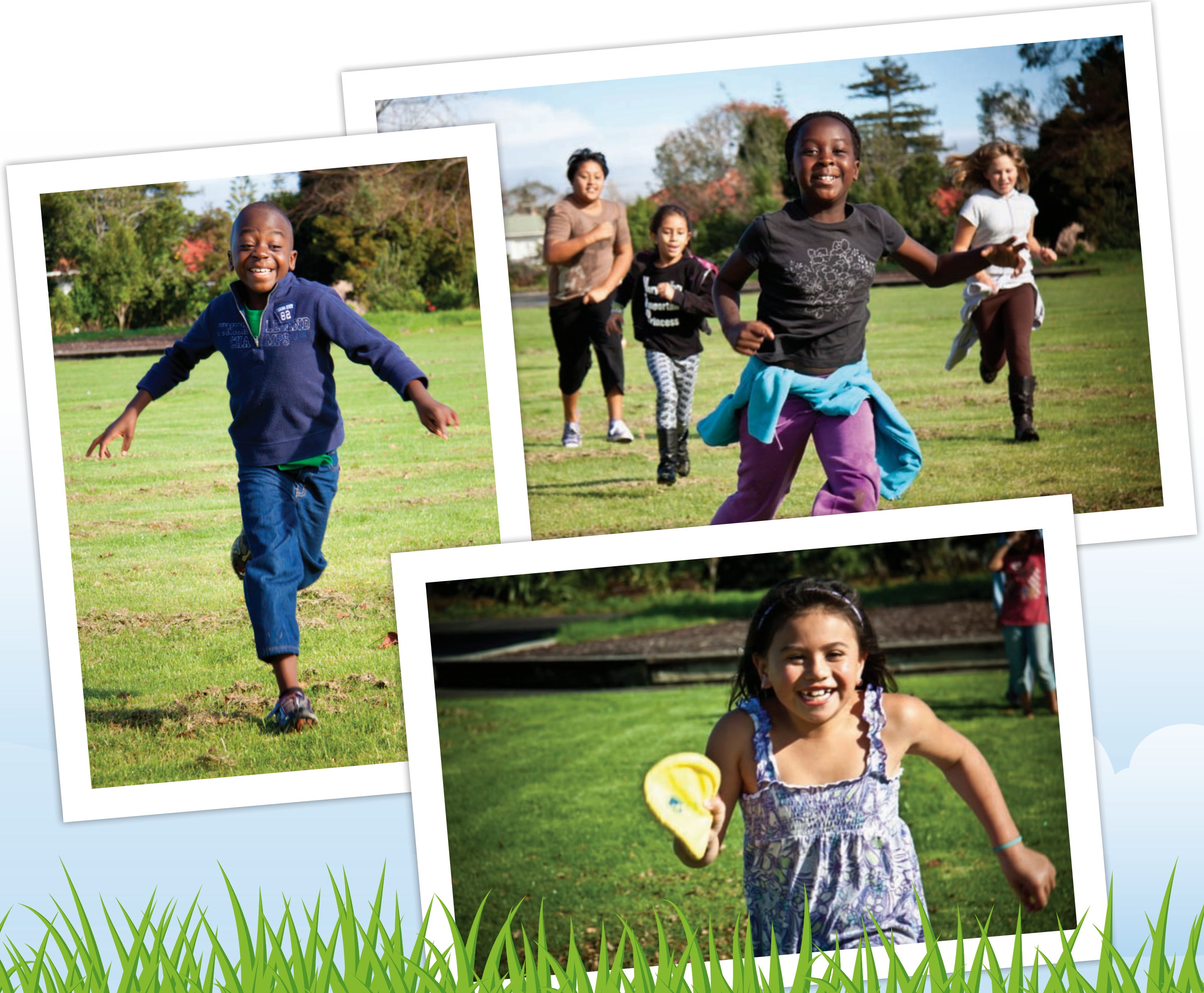

NOTES:

REMEMBER to post your photos and blogs on the Healthly Homework website. www.healthyhomework.org.nz

Healthy Homework

Proudly supported by AUT UNIVERSITY
